# Dihydronaphthofurans: synthetic strategies and applications

**DOI:** 10.1039/c9ra09987e

**Published:** 2020-02-05

**Authors:** Abolfazl Olyaei, Mahdieh Sadeghpour

**Affiliations:** Department of Chemistry, Payame Noor University (PNU) PO BOX 19395-4697 Tehran Iran; Department of Chemistry, Takestan Branch, Islamic Azad University Takestan Iran olyaei_a@pnu.ac.ir

## Abstract

Dihydronaphthofurans (DHNs) are an important class of arene ring-fused furans which are widely found in many natural and non-natural products and drug candidates with relevant biological and pharmacological activities. Furthermore, vinylidene-naphthofurans exhibit photochromic properties when exposed to UV or sun light at room temperature. For these reasons, a vast array of synthetic procedures for the preparation of dihydronaphthofurans including annulation of naphthols with various reagents, cycloaddition reactions ([3 + 2], [4 + 1] and Diels–Alder), intramolecular transannulation, Friedel–Crafts, Wittig, Claisen rearrangement, neophyl rearrangement and other reactions under various conditions have been developed over the past decades. This review aims to describe the different strategies developed so far for the synthesis of dihydronaphthofurans and their applications. After a brief introduction to the types of dihydronaphthofurans and their biological activities, the different synthetic approaches such as chemical, photochemical, and electrochemical, methods are described and organized on the basis of the catalysts and the other reagents employed in the syntheses. The subsequent section focuses on biological and pharmacological applications and photochromic properties of the target compounds.

## Abstract 2

Dihydronaphthofurans (DHNs) are an important class of arene ring-fused furans which are widely found in many natural and non-natural products and drug candidates with relevant biological and pharmacological activities.

## Graphical abstract

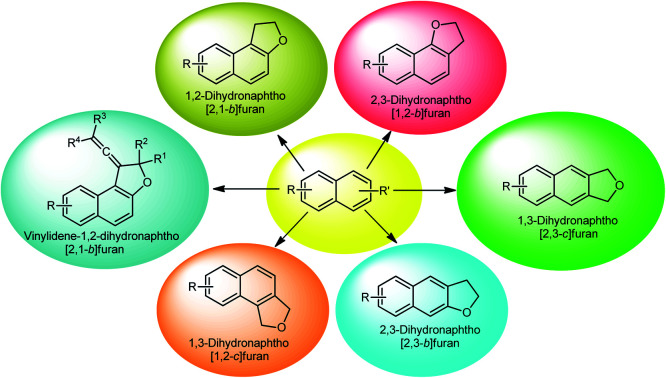

## 1. Introduction

**Fig. 1:**
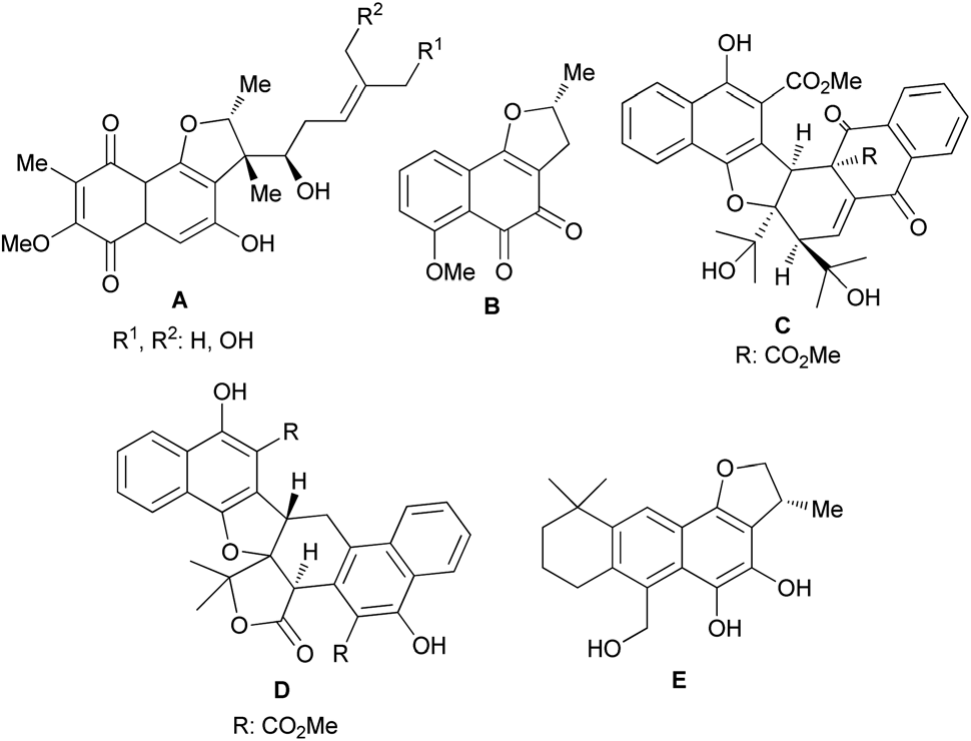
Naturally occurring molecules bearing the dihydronaphthofurans.

Furans are five-membered aromatic heterocycles containing one oxygen atom that are commonly found in many important compounds such as natural products, pharmaceuticals and polymers. Moreover, furans can be utilized as synthetic intermediates to access other useful compounds. The synthesis of this fundamental structural building block has received significant attention and a wide variety of approaches are available to the synthetic practitioner.^1^ Arene ring-fused furan derivatives such as dihydronaphthofurans (DHNs) have attracted widespread interest in view of their presence in many important natural and non-natural products. Natural products with DHN moieties have been shown to have a wide range of biological and pharmacological properties.^2–4^ For example, furaquinocins (A), consisting of highly oxygenated *p*-quinone rings, are antihypertensive, cytotoxic against HeLa S3 and B16 melanoma cells, and also inhibit platelet aggregation and coagulation.^2^ In contrast, (−)-nocardione (B) with an *o*-quinone moiety is a Cdc25B tyrosine phosphatase inhibitor with moderate antifungal and cytotoxic activity.^3^ Also, rubioncolin A (C) and rubioncolin B (D) were isolated from *Rubia oncotricha* in racemic forms.^4^ They are also found in *Rubia cordifolia*, which is used in traditional Korean medicine to treat coughs, bladder and kidney stones, joint inflammation, uterine hemorrhage, and uteritis.^5^ Interestingly, the electron rich catechol derivative aegyptinone (E) shows antibacterial and antifungal activity^6^ (Fig. 1).

**Fig. 2:**
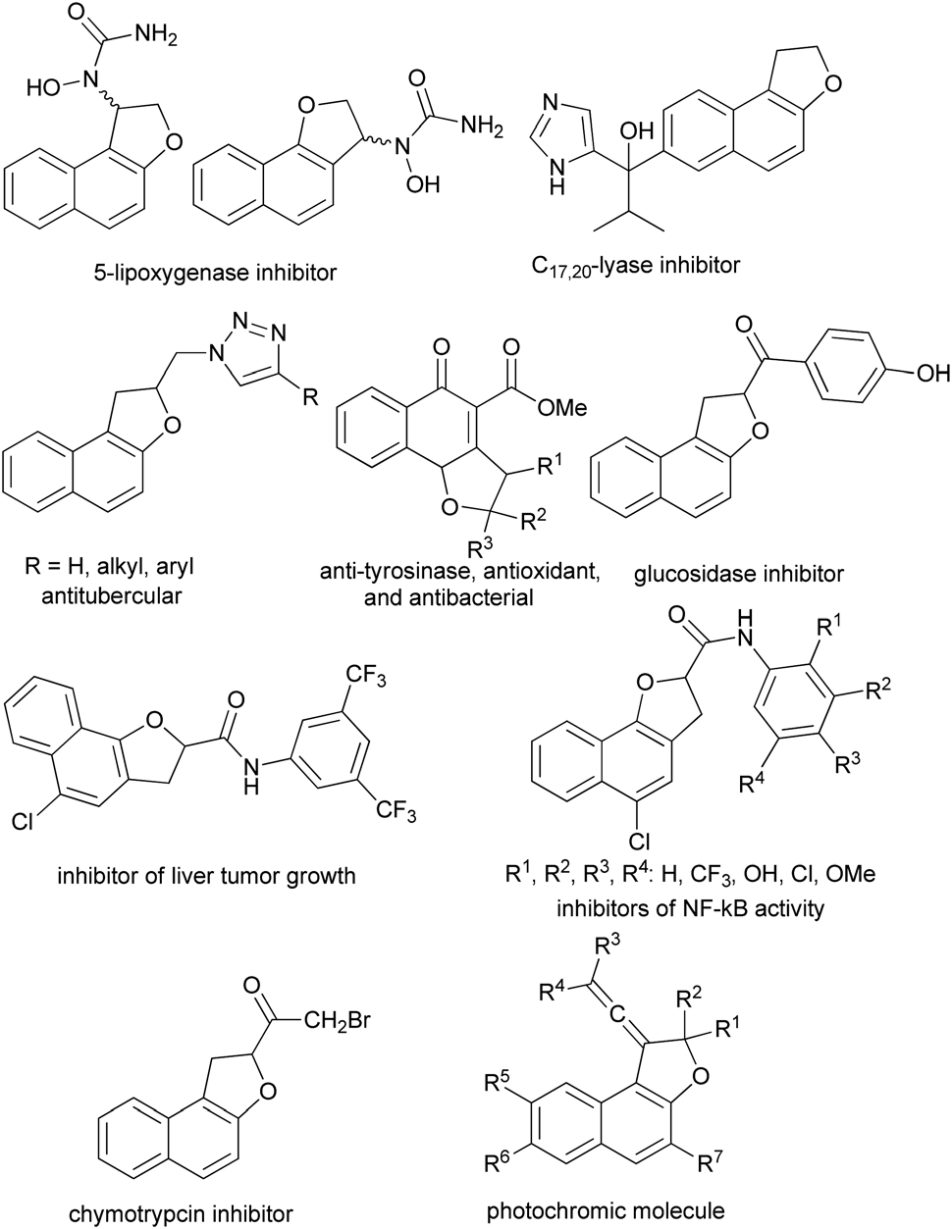
Examples of biological, pharmacological and photochromic products containing dihydronaphthofuran moiety.

Furthermore, some of the synthesized dihydronaphthofuran derivatives exhibit a variety of interesting biological and pharmacological properties including 5-lipoxygenase inhibitor,^7^ C_17,20_ lyase inhibitors,^8^ antitubercular activity against *Mycobacterium tuberculosis* H_37_Rv,^9^ anti-tyrosinase, antioxidant, and antibacterial,^10^ inhibitors of NF-_k_B activity,^11^ α-glucosidase inhibitors,^12^ inhibitor of α-chymotrypsin^13^ and anti cancer activities (liver tumor growth inhibitors).^14^ Moreover, vinylidene-naphthofurans are a new class of polycyclic compounds that exhibit photochromic properties when exposed to the UV or sunlight at room temperature and adsorbed in silica gel or dissolved in acidified alcoholic solutions (Fig. 2).^15–20^

**Fig. 3:**
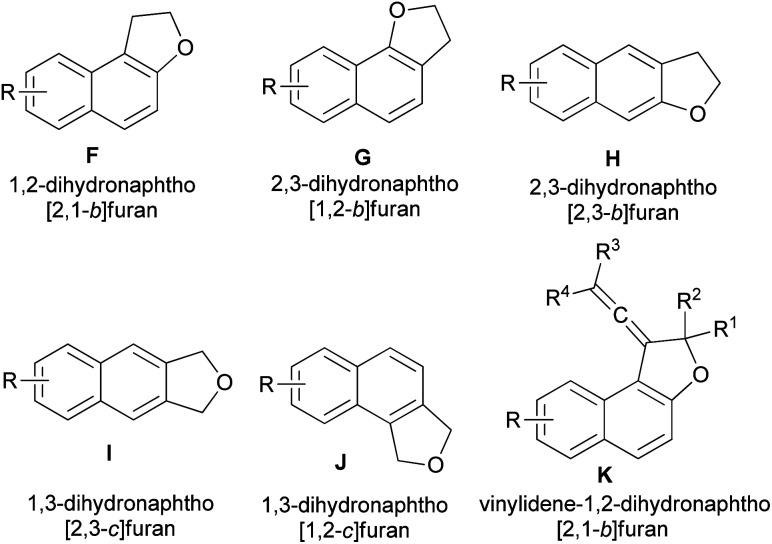
Structures of dihydronaphthofurans F–K.

This wide range of biological activities and properties has stimulated interest in the synthesis of dihydronaphthofuran derivatives. During the past decades, several synthetic approaches to 2,3-dihydronaphtho[2,1-*b*]furan, 2,3-dihydronaphtho[1,2-*b*]furan, 2,3-dihydronaphtho[2,3-*b*]furan, 1,3-dihydronaphtho[2,3-*c*]furan, 1,3-dihydronaphtho[1,2-*c*]furan and vinylidene-1,2-dihydronaphtho[2,1-*b*]furan derivatives (F–K) (Fig. 3) have been reported. The general methods used for the synthesis of these compounds include chemical, photochemical and electrochemical methods have been described and organized on the basis of the catalysts and the other reagents. To the best of our knowledge, there is no review on synthesis of dihydronaphthofuran derivatives. Hence, the main purpose of this review is to show all types of reactions for the synthesis of these heterocyclic compounds, such as annulation of naphthols with various reagents, cycloaddition reactions ([3 + 2], [4 + 1] and Diels–Alder), intramolecular transannulation, Friedel–Crafts, Wittig, Claisen rearrangement, neophyl rearrangement and the other reactions in various conditions and their biological, pharmacological and photochromic properties.

## 2. Synthesis of dihydronaphthofurans

### 2.1. Base-catalyzed synthesis

**Scheme 1:**
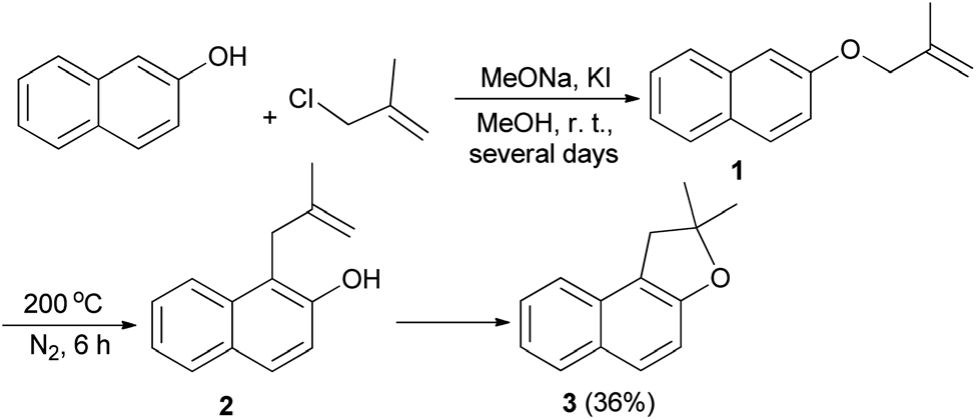
Preparation of 1,2-dihydro-2,2-dimethylnaphtho[2,1-*b*]furan (3).

The first synthesis of 1,2-dihydronaphtho[2,1-*b*]furan has been reported by Mckusick in 1948. Sodium methoxide and potassium iodide promoted condensation of 2-naphthol with β-methyl allyl chloride in methanol at room temperature for several days afforded β-methylallyl-2-naphthyl ether (1). Claisen rearrangement of 1 led to the formation of intermediate 2. The latter underwent cyclization to provide 1,2-dihydro-2,2-dimethylnaphtho[2,1-*b*]furan (3) in 36% yield (Scheme 1).^21^

**Scheme 2:**
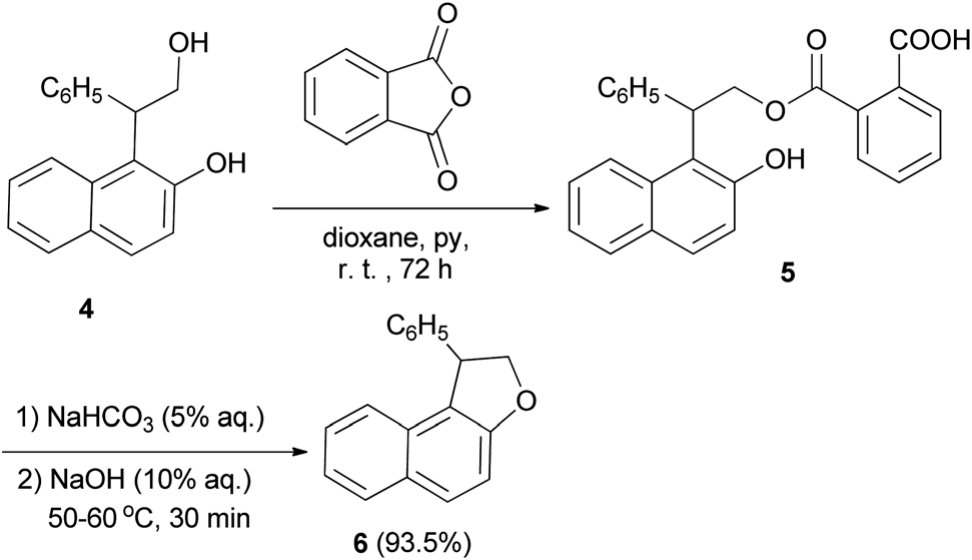
Base-catalyzed synthesis of 1-pheny1-1,2-dihydronaphtho[2,1-*b*]furan (6).

Guss and Jules reported that the reaction of 2-(2-hydroxy-1-naphthyl)-2-phenylethanol (4) with phthalic anhydride in the presence of pyridine in dioxane at room temperature for 72 h, led to the formation of acid phthalate 5. By heating in 5% sodium bicarbonate and 10% sodium hydroxide at 50–60 °C for 30 min the acid phthalate 5 was converted into the cyclized product, 1-pheny1-1,2-dihydronaphtho[2,1-*b*]furan (6) (Scheme 2).^22^

**Scheme 3:**
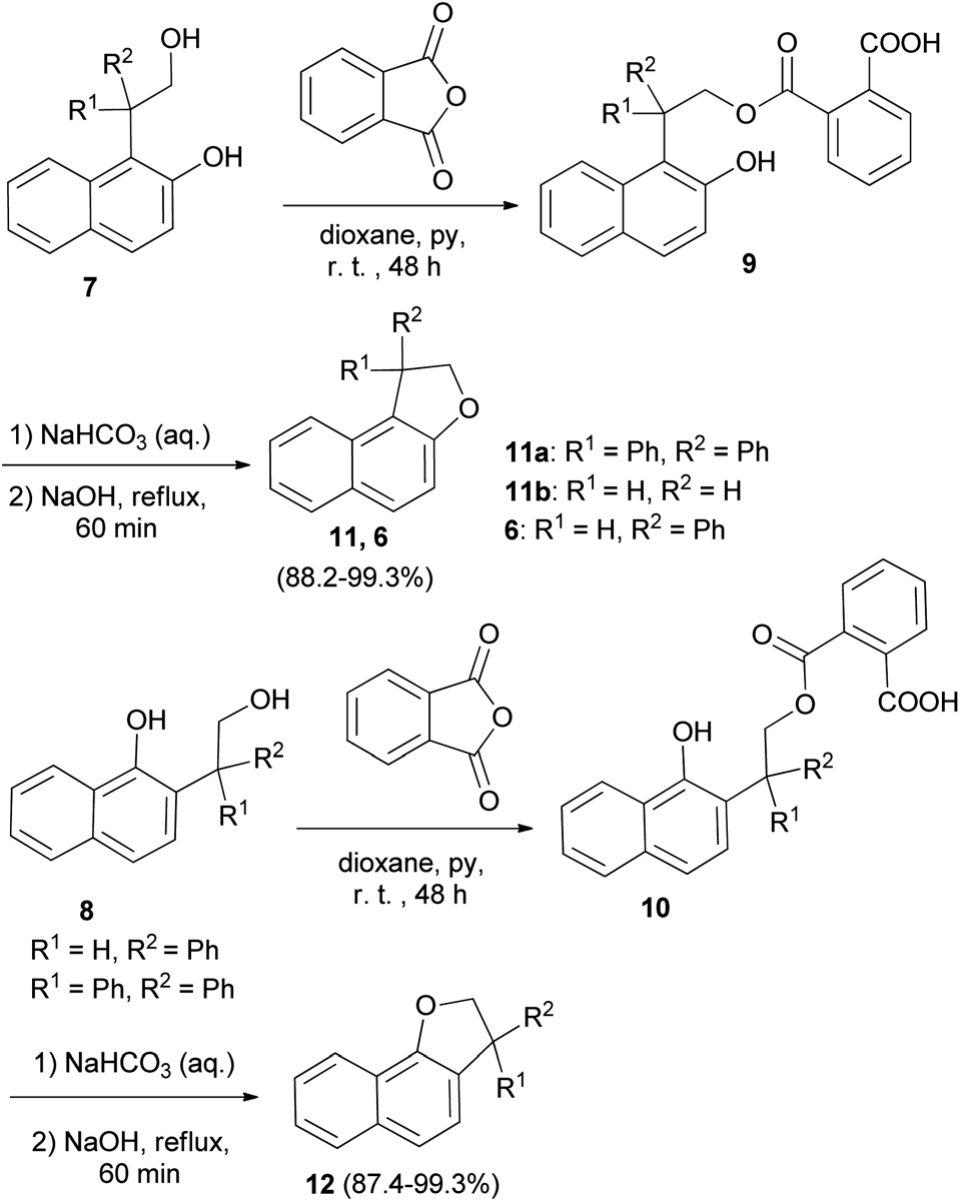
Synthesis of 1,2-dihydronaphtho[2,1-*b*]furans 11a–b, 6 and of 2,3-dihydronaphtho[1,2-*b*]furans 12.

In a similar fashion, phenol–alcohols 7 and 8 were converted into the acid phthalates 9 and 10 by allowing a solution of the phenol alcohols, phthalic anhydride, pyridine and dioxane to stand at room temperature for 48 hours. Then, addition of weaker solution of sodium hydroxide to the bicarbonate solution of the esters 9 and 10 and the slurry were heated at reflux temperature for 1 hour afforded 1,2-dihydronaphtho[2,1-*b*]furans 11a–b, 6 and 2,3-dihydronaphtho[1,2-*b*]furans 12 in 88.2–99.3% and 87.4–99.3% yields, respectively (Scheme 3).^23^

**Scheme 4:**
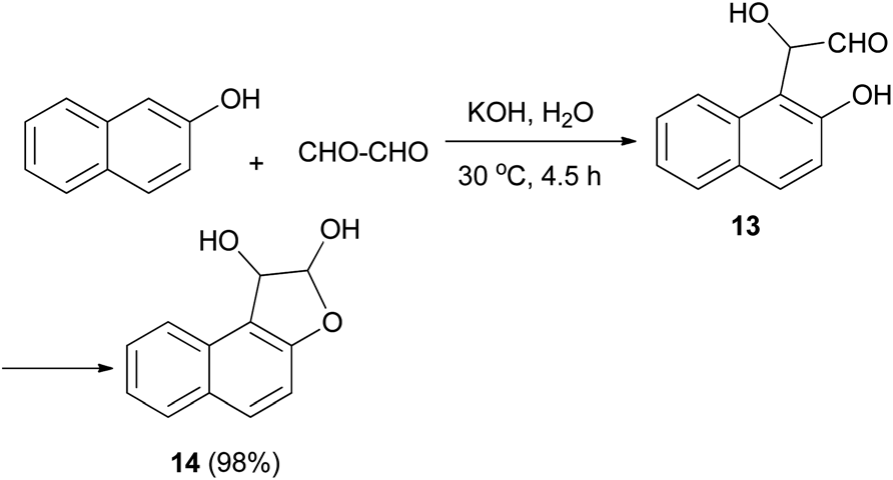
Synthesis of 1,2-dihydronaphtho[2,1-*b*]furan-1,2-diol (14).

In 1994, Kito *et al.* explored the reaction of 2-naphthol with glyoxal in the presence of KOH in aqueous media at 30 °C for 4.5 h, which furnished 1,2-dihydronaphtho[2,1-*b*]furan-1,2-diol (14) in 98% yield. Hemiacetal 14 was obtained *via* cyclization of intermediate 13 (Scheme 4).^24^

**Scheme 5:**
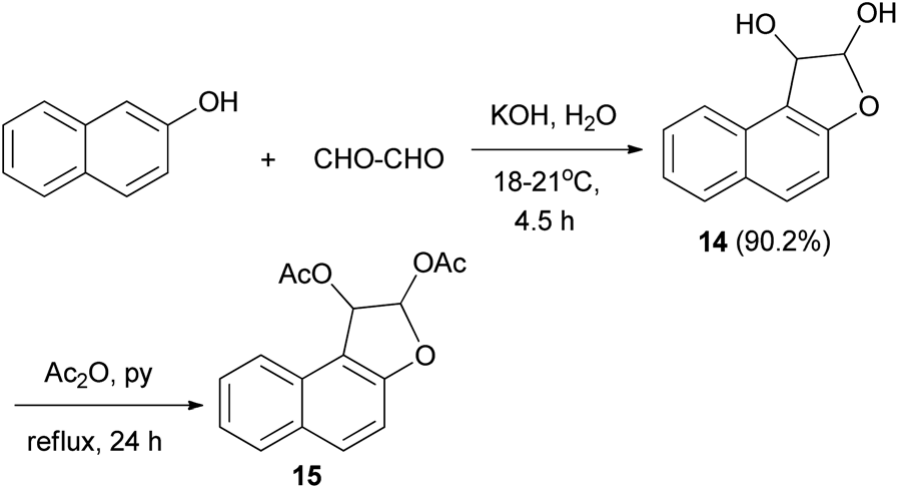
Synthesis of 1,2-diacetoxy-l,2-dihydronaphtho[2,1-*b*] furan (15).

In a similar manner, dihydronaphthofuran 14 could be obtained *via* treatment of 2-naphthol with aqueous glyoxal (40%) in aqueous solution of KOH at 18–21 °C for 4.5 h in 90.2% yield. Moreover, 1,2-diacetoxy-l,2-dihydronaphtho[2,1-*b*]furan (15) was efficiently achieved from 14 in acetic anhydride and pyridine under reflux for 24 h (Scheme 5).^25^

**Scheme 6:**
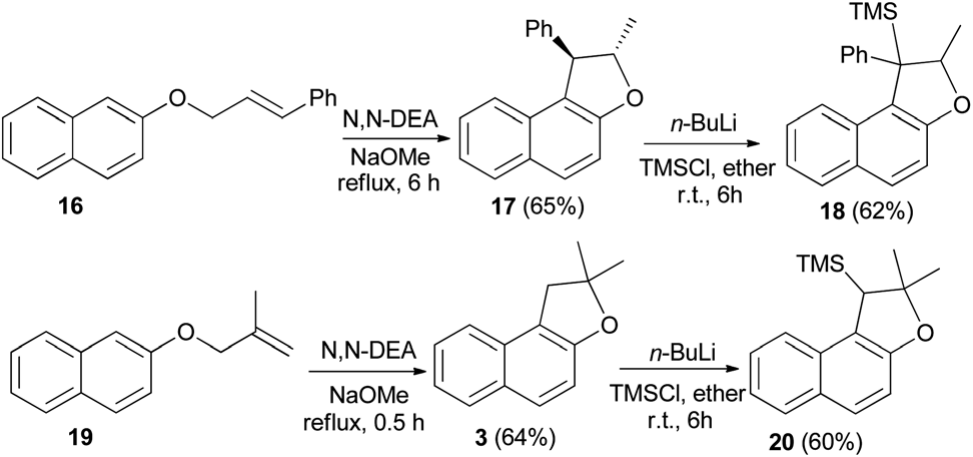
Synthesis of 1,2-dihydronaphtho[2,1-*b*]furan derivatives 3, 17, 18 and 20.

Saidi *et al.*^26^ reported that the reaction of 2-naphthol with alkenyl halide in acetone in the presence of K_2_CO_3_ and KI gave the corresponding naphthyl ethers 16 and 19. Naphthyl ethers in *N*,*N*′-diethyl aniline (*N*,*N*′-DEA) and sodium methoxide under reflux for 0.5–8 h afforded dihydronaphthofurans 17 and 3 in 65 and 64% yields, respectively. In order to prepare trimethylsilyl-substituted dihydronaphthofurans 18 and 20, dihydronaphthofurans 17 and 3 were treated with *n*-BuLi and then with chlorotrimethylsilane (TMS-Cl) in diethyl ether at room temperature for 6 h (Scheme 6).

**Scheme 7:**
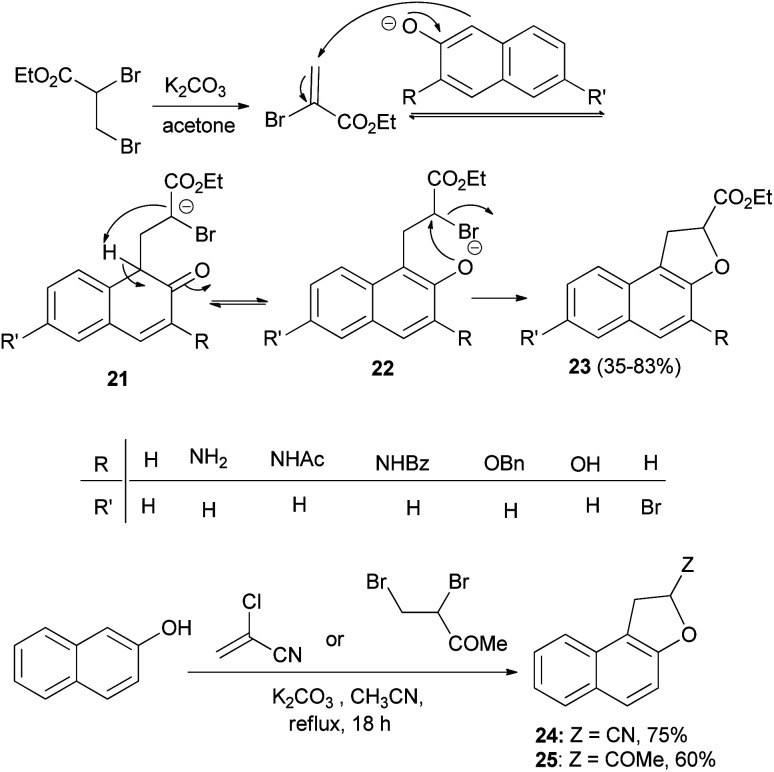
One-step synthesis of 1,2-dihydronaphtho[2,1-*b*]furans 23–25.

A one-step synthesis of ethyl 1,2-dihydronaphtho[2,1-*b*]furan-2-carboxylates 23 from substituted naphthols and ethyl 2,3-dibromopropanoate in the presence of K_2_CO_3_ in refluxing acetone for 18 h has been reported by Merour *et al.*^27^ First, ethyl 2,3-dibromopropanoate is easily transformed *in situ* into ethyl-2-bromoacrylate with potassium carbonate in refluxing acetone. Then, a Michael-type addition of the naphthalenolate to the 2-bromoacrylate generates the C–C bond forming intermediate 21. Aromatization and formation of the 2-naphthalenolate anion gives the intermediate 22. This is followed by an intramolecular nucleophilic substitution on the carbon bearing the bromo atom in 22 affording the gave the corresponding product 23 in 35–83% yields. Following the same methodology, the reaction of 2-naphthol with 2-chloroacrylonitrile and 3,4-dibromobutan-2-one in refluxing acetonitrile for 18 h afforded 24 and 25 in 75 and 60% yields, respectively (Scheme 7).

**Scheme 8:**
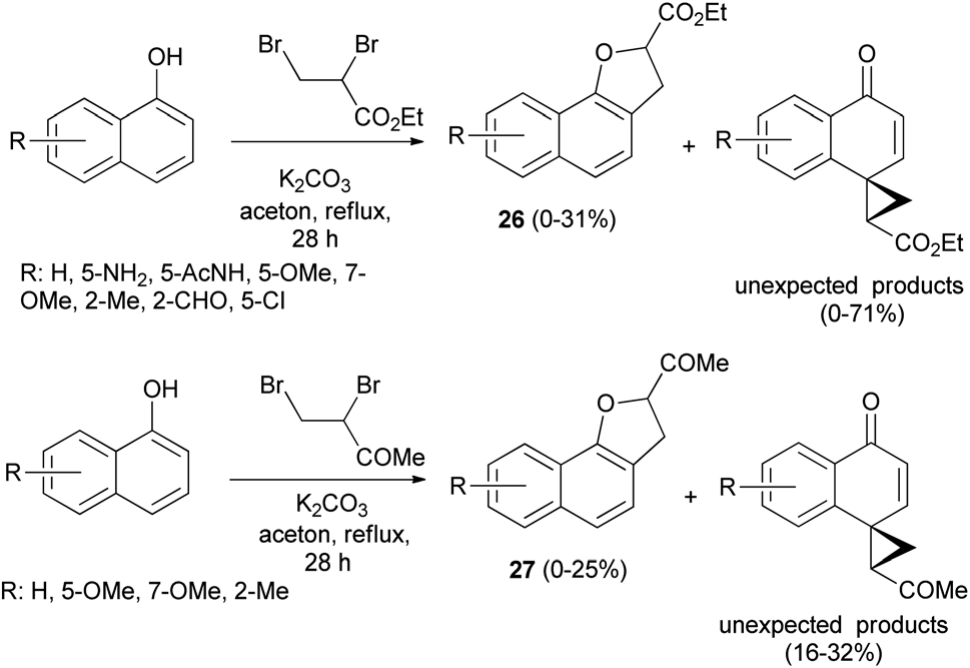
Synthesis of 2,3-dihydronaphtho[1,2-*b*]furans 26 and 27.

Treatment of 1-naphthol derivatives with ethyl 2,3-dibromopropanoate in the presence of K_2_CO_3_ in refluxing acetone for 28 h led to the formation of the expected ethyl 2,3-dihydronaphtho[1,2-*b*]furan-2-carboxylates 26 in 0–31% yields and unexpected ethyl 4′-oxospiro[cyclopropane-1,1′(4′*H*)-naphthalene]-2-carboxylates in 0–71% yields besides starting materials. Also, the reaction of 1-naphthols with 3,4-dibromobutan-2-one by applying the same methodology gave 2,3-dihydronaphtho[1,2-*b*]furan-2-yl ketones 27 in 0–25% yields and unexpected products in 16–32% yields (Scheme 8).^28^ Formation of 26 and 27 took place according to similar reported mechanism in Scheme 7.^27^

**Scheme 9:**
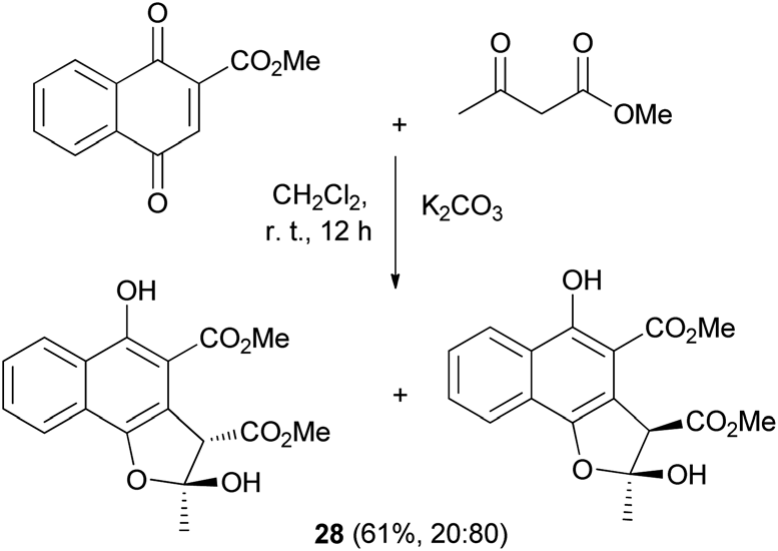
Synthesis of *cis* and *trans* 2,3-dihydronaphtho[1,2-*b*]furan derivatives 28.

An efficient procedure for the conjugate addition of methyl acetoacetate to 2-(methoxycarbonyl)-1,4-naphthoquinone using K_2_CO_3_ has been developed. The reaction was carried out in CH_2_Cl_2_ at room temperature for 12 h yielded 61% of a mixture of *cis* and *trans* (20 : 80) 2,3-dihydronaphtho[1,2-*b*]furan derivatives 28 (Scheme 9).^29^

**Scheme 10:**
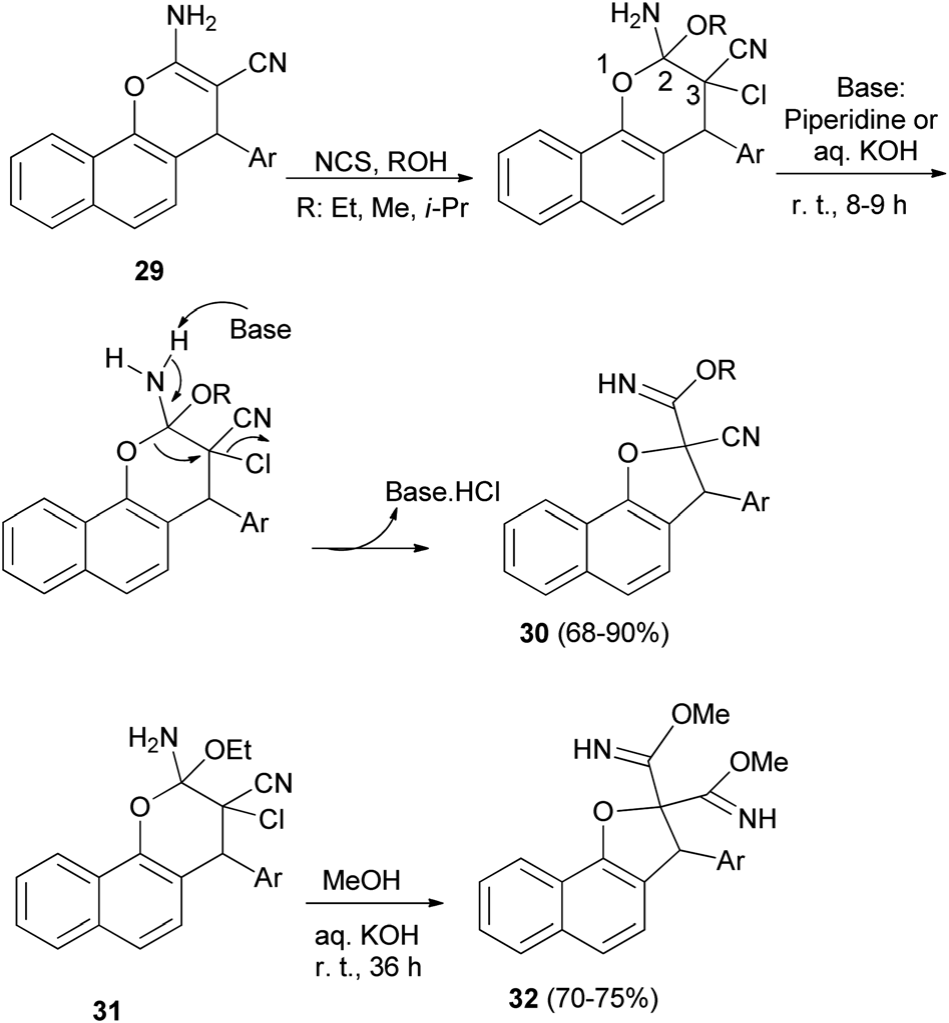
Synthesis of dihydronaphthofurans 30 and 32 from 4*H*-pyran derivatives.

Alla *et al.*^30^ noted that one-pot reaction of 2-amino-4*H*-pyran derivatives 29 with *N*-chlorosuccinimide and a base (piperidine or aqueous KOH) in alcohol medium at room temperature for 8–9 h gave dihydronaphthofurans 30 in 68–90% yields. Plausible mechanism for the formation of 30 has been arrived at Scheme 10. Initially aminopyran undergoes oxidative difunctionalization with NCS in the presence of an alcohol solvent. Subsequent addition of base to the aminopyran leads to proton abstraction from the amino group. This leads to a cascade pyran ring opening by the cleavage of the (C_2_–O_1_) bond, and ring closure of the (O_1_–C_3_) bond to dihydrofuran *via* elimination of HCl. The sequential elementary processes lead to formation of the ring contracted dihydrofuran carbimidate ester 30. In a similar manner, highly functionalized pyran derivatives 31 were treated with methanolic KOH (1 N) at room temperature for 36 h to obtain the products 32 in good yields (70–75%) (Scheme 10).

**Scheme 11:**
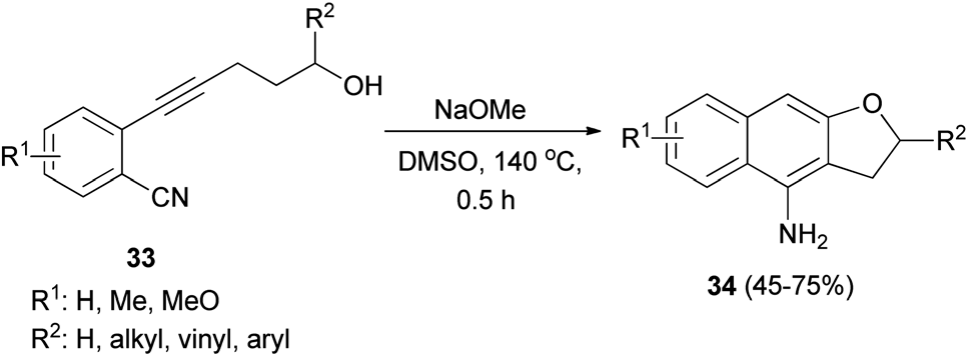
Synthesis of dihydronaphthofurans 34.

**Scheme 12:**
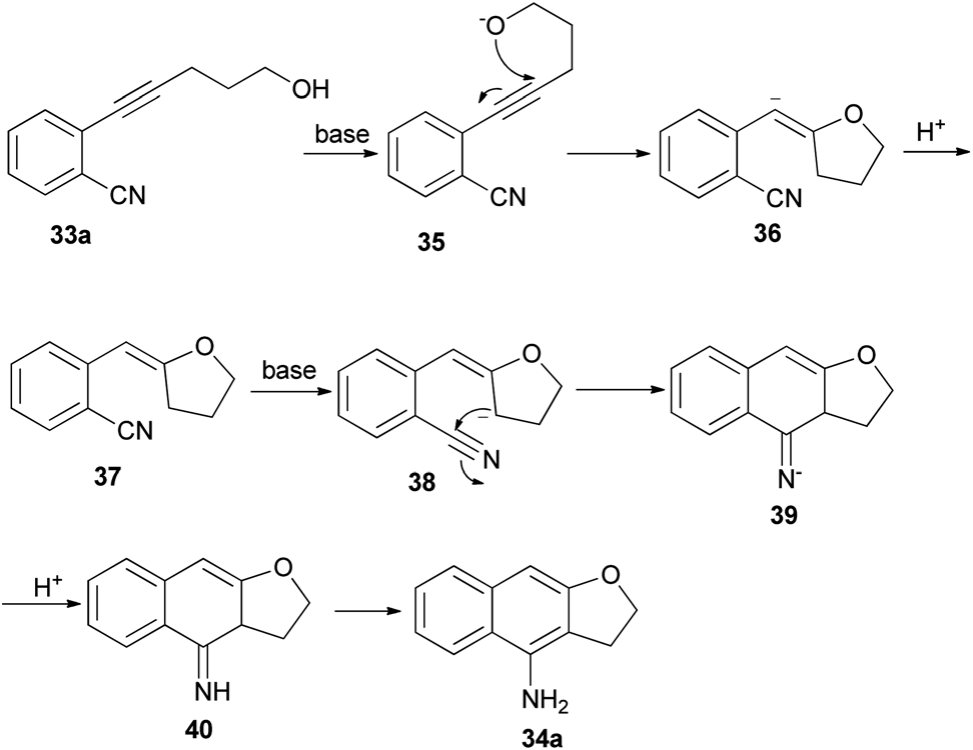
Proposed mechanism for the formation of 34a.

**Scheme 13:**
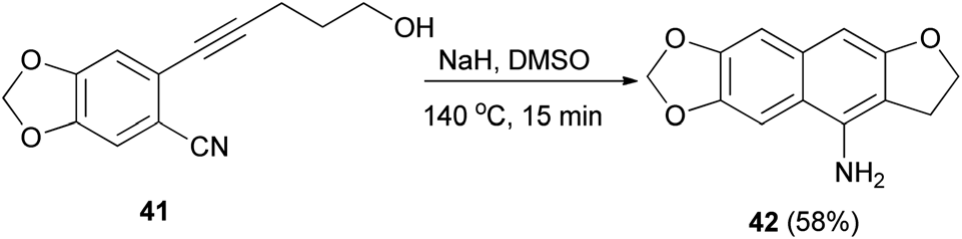
Preparation of 2,3-dihydronaphtho[2,3-*b*]furan 42.

Base-mediated cyclization reactions of 2-(5-hydroxy-1-pentynyl)benzonitriles 33 to 4-amino-2,3-dihydronaphtho[2,3-*b*]furans 34 have been reported by Wu *et al.*^31^ Treatment of 33 with NaOMe in DMSO at 140 °C for 0.5 h gave the desired products 34 in 45–75% yields (Scheme 11). The proposed mechanism for the cyclization of 33a to 34a is shown in Scheme 12. The first step is the deprotonation of 33a with base to form alkoxide 35 that would undergo intramolecular 5-*exo*-dig cyclization to form the vinyl anion 36. The vinyl anion 36 could undergo direct proton transformation to give 37. Deprotonation of 37 with base to form anion 38. Then, the electrocyclic ring closure reaction to give 39. Finally protonation of 39 to give imine 40 and following the imine–enamine tautomerization, to convert the imine 40 to the final product 34a.^31^ Also, cyclization reaction of 41 with NaH in DMSO at 140 °C for 15 min afforded product 42 in 58% yield (Scheme 13).^31^

**Scheme 14:**
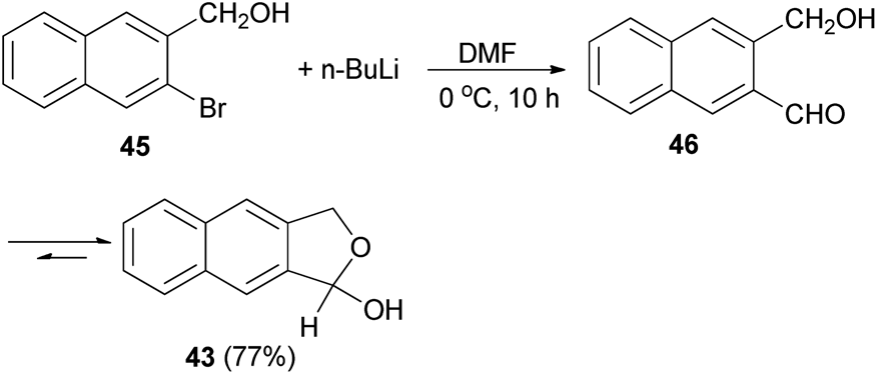
Preparation of dihydronaphthofuran 43.

**Scheme 15:**
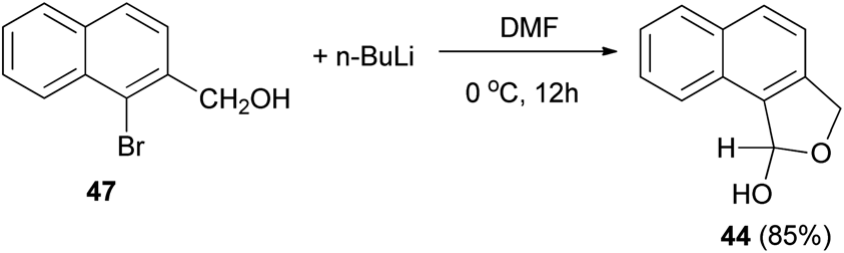
Preparation of dihydronaphthofuran 44.

Several reports on the synthesis of hemiacetals of dihydronaphthofurans have been described by Dibble *et al.* 1,3-Dihydro-1-hydroxynapbtho[2,3-*c*]furan (43) and 1,3-dihydro-*l*-hydroxynaphtho[1,2-*c*]furan (44) could be successfully prepared in 77% and 85% yields, respectively. In this reaction, treatment of bromonaphthalene methanol (45) in dry diethyl ether with *n*-butyl lithium in hexane at −78 to 0 °C for 30 min afforded 3-(hydroxymethyl)-2-naphthaldehyde 46, which on treatment with dry DMF at 0 °C for 10 h led to dihydronaphthofuran 43 (Scheme 14). In a similar manner, dihydronaphthofuran 44 was prepared from bromonaphthalene methanol 47 in 85% yield as shown in Scheme 15.^32^

**Scheme 16:**
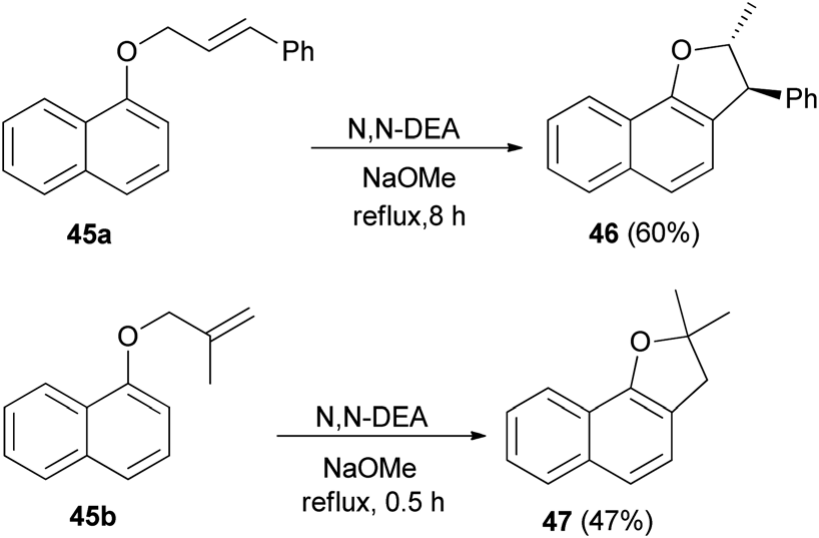
Synthesis of 2,3-dihydronaphtho[1,2-*b*]furan derivatives 46 and 47.

Treatment of 1-naphthol with alkenyl halide in acetone in the presence of K_2_CO_3_ and KI gave the corresponding allyl naphthyl ethers 45a–b. Heating of 45 in *N*,*N*′-diethyl aniline (*N*,*N*′-DEA) and sodium methoxide under reflux for 0.5–8 h afforded 2,3-dihydronaphtho[1,2-*b*]furan derivatives 46 and 47*via* Claisen rearrangement in 60 and 47% yields, respectively (Scheme 16).^26^

### 2.2. Acid-catalyzed synthesis

**Scheme 17:**
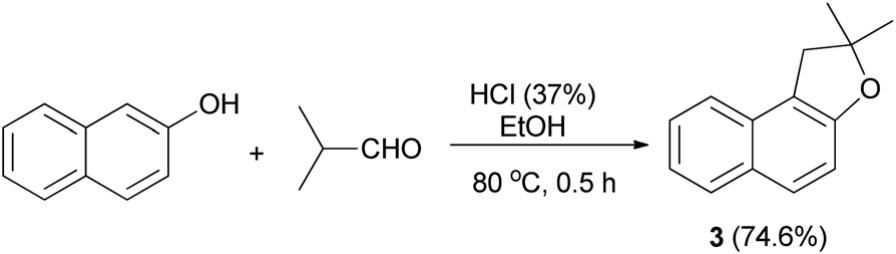
Acid-catalyzed synthesis of dihydronaphthofuran 3.

In 1971, Martini reported that preparation of dihydronaphthofuran 3 was accomplished by the treatment of 2-naphthol with isobutylaldehyde using HCl in EtOH at 80 °C for 0.5 h in 74.6% yield (Scheme 17).^3^

**Scheme 18:**
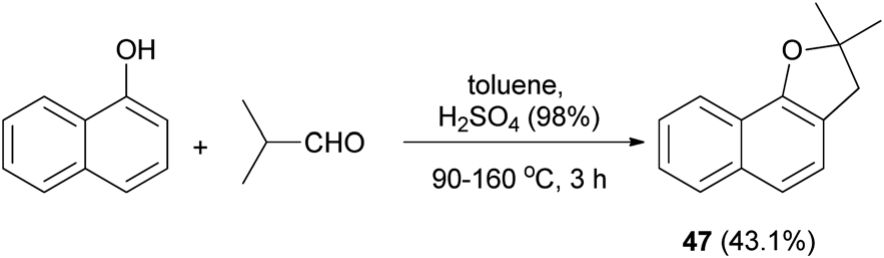
Acid-catalyzed synthesis of 2,3-dihydro-2,2-dimethylnaphthol[1,2-*b*] furan (47).

In a similar fashion, synthesis of 2,3-dihydro-2,2-dimethylnaphthol[1,2-*b*] furan (47) in 43.1% yield has been accomplished by the treatment of 1-naphthol with isobutylaldehyde using H_2_SO_4_ in toluene at 90–160 °C for 3 h (Scheme 18).^33^

**Scheme 19:**
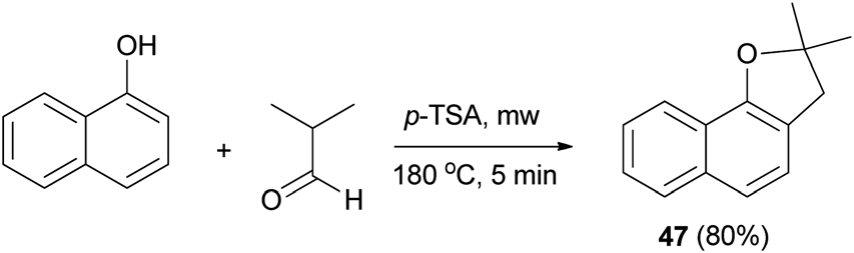
*p*-TSA-catalyzed synthesis of dihydronaphthofuran 47.

Similarly, dihydronaphthofuran 47 was obtained *via* the reaction of isobutyraldehyde with 1-naphthol in the presence of catalytic amount of *p*-TSA under closed vessel solvent-free microwave irradiation conditions at 180 °C for 5 min in 80% yield (Scheme 19).^34^

**Scheme 20:**
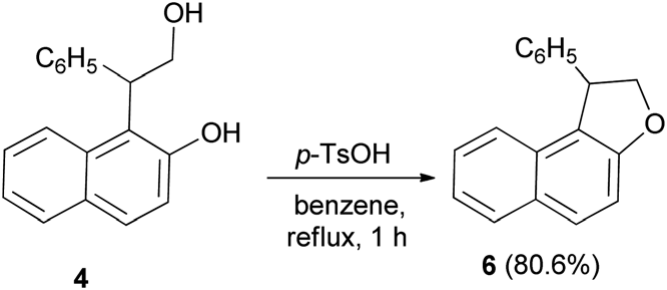
Acid-catalyzed synthesis of 1-pheny1-1,2-dihydronaphtho[2,1-*b*]furan (6).

Treatment of 4 with *p*-toluenesulfonic acid monohydrate in benzene under reflux condition for one hour with simultaneous removal of 50 cm^3^ of benzene containing the water formed, gave dihydronaphthofuran 6 in 80.6% yield (Scheme 20).^22^

**Scheme 21:**
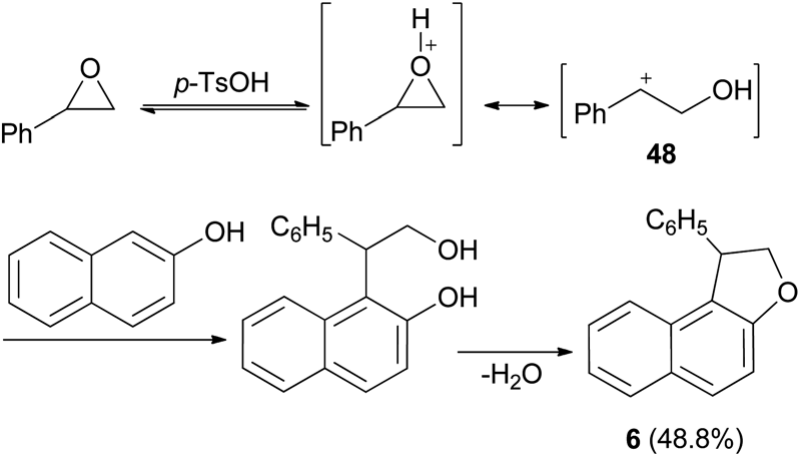
Synthesis of dihydronaphthofuran 6.

It was also shown that dihydronaphthofuran 6 was obtained by the reaction of styrene oxide with 2-naphthol in refluxing benzene for 1 h in 48.8% yield (Scheme 21). This reaction proceed *via* the acid catalyzed unimolecular ring opening of styrene oxide, followed by nucleophilic attack of naphthol to the resulting adduct 48 and cyclization, then elimination of water afforded the final product 6.^35^

**Scheme 22:**
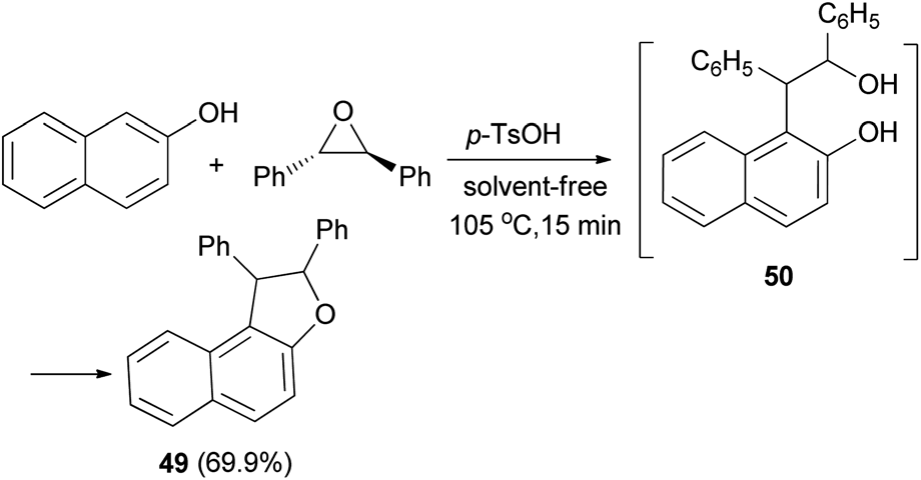
Acid-catalyzed synthesis of 1,2-diphenyl-1,2-dihydronaphtho[2,1-*b*]furan (49).

In 1953, Guss *et al.*^36^ have obtained 1,2-diphenyl-1,2-dihydronaphtho[2,1-*b*]furan (49) in 69.9% yield from the reaction of 2-naphthol and *trans*-stilbene oxide by using *p*-toluenesulfonic acid monohydrate as catalyst in 105 °C for 15 min under solvent-free condition. Presumably a phenol–alcohol 50 was first formed and then cyclized under these reaction conditions to the desired product 49 (Scheme 22).

**Scheme 23:**
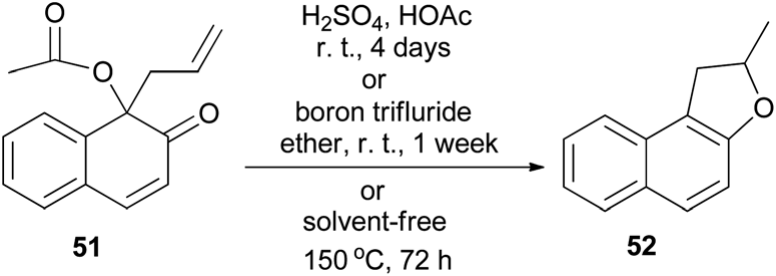
Rearrangement of 1-acetoxy-1-allyl-2-naphthalenone (51) to dihydronaphthofuran 52.

Miller and Lin noted that rearrangement of 1-acetoxy-1-allyl-2-naphthalenone (51) in 10% sulfuric acid in acetic acid at room temperature for 4 days, or in boron trifluoride etherate at room temperature for one week, gave 2-methyl-1,2-dihydronaphtho[2,1-*b*]furan (52) as the only product. Similarly, attempted thermal rearrangement at 150 °C for 3 days in the absence of solvent afforded 52 (Scheme 23).^37^

**Scheme 24:**
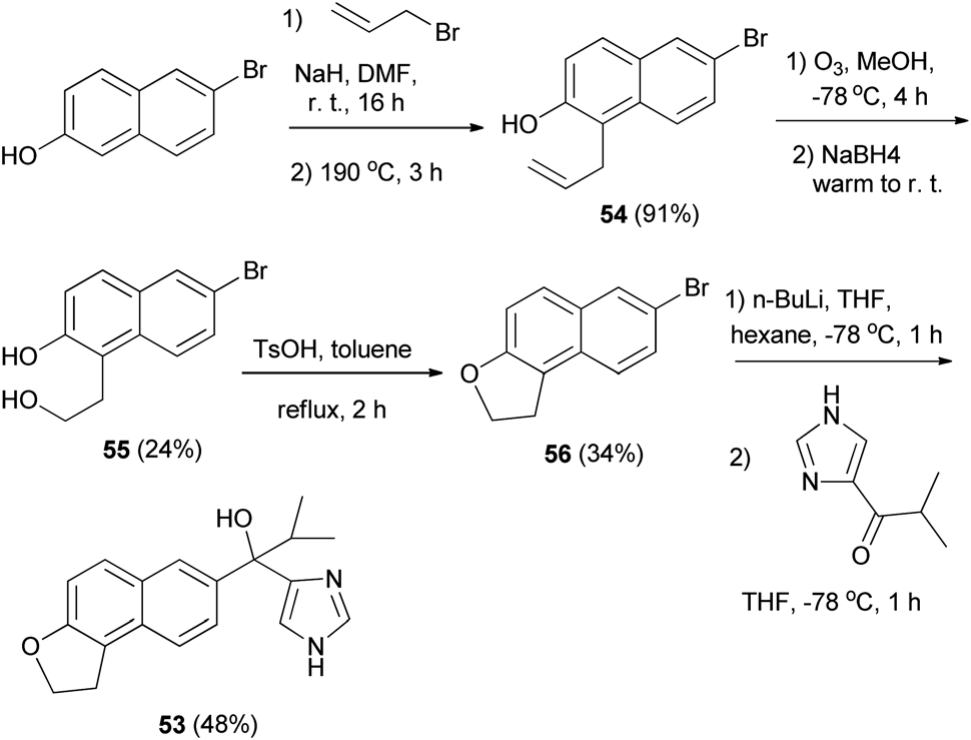
Synthesis of 1,2-dihydronaphtho[2,1-*b*]furan derivatives 53 and 56.

Matsunaga *et al.*^8^ developed a strategy for the synthesis of 1,2-dihydronaphtho[2,1-*b*]furan 53. The reaction was carried out *via* the key intermediate 54 as shown in Scheme 24. Allylation of 6-bromo-2-hydroxynaphthalene followed by a Claisen rearrangement at 190 °C gave the 1-allyl-2-naphthol 54. Ozonolysis of 54 followed by treatment with NaBH_4_ afforded the diol 55 and intramolecular dehydration under acidic conditions provided 56. Lithiation of 1,2-dihydronaphthofuran 56 and then addition to the imidazolyl isopropyl ketone furnished 1,2-dihydronaphtho[2,1-*b*]furan 53 in 48% yield.

**Scheme 25:**
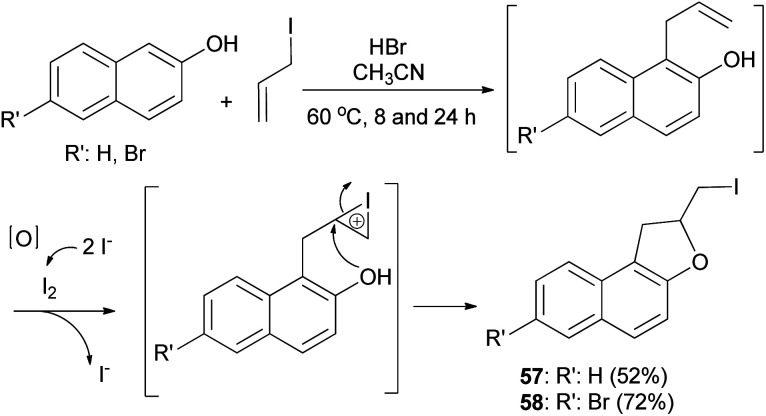
HBr-catalyzed preparation of 1,2-dihydronaphtho[2,1-*b*] furans 57 and 58.

Hydrobromic acid-catalyzed Friedel–Crafts type reactions of 2-naphthols with allyl iodide in CH_3_CN at 60 °C for 8 and 24 h led to the formation of dihydronaphthofuran 57 and 7-bromo-2-(iodomethyl)-1,2-dihydronaphtho[2,1-*b*]furan (58) in 52% and 72% yields, respectively. Formation of products by reaction of 2-naphthols with allyl iodide is assumed to proceed *via* allylation of naphthols followed by iodocyclization to produce the corresponding products (Scheme 25).^38^

**Scheme 26:**
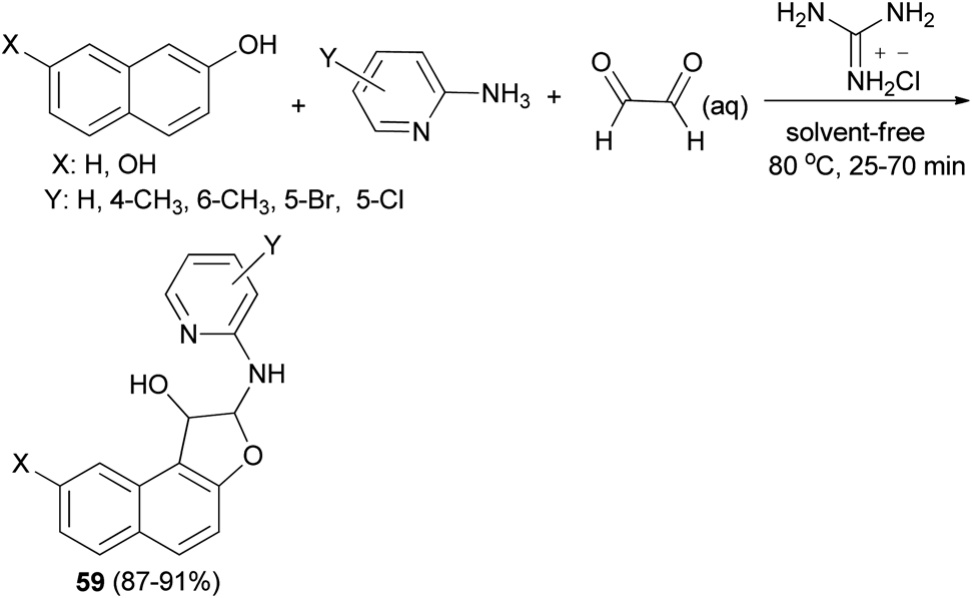
Guanidinium chloride catalyzed synthesis of 1,2-dihydronaphtho[2,1-*b*]furans 59.

**Scheme 27:**
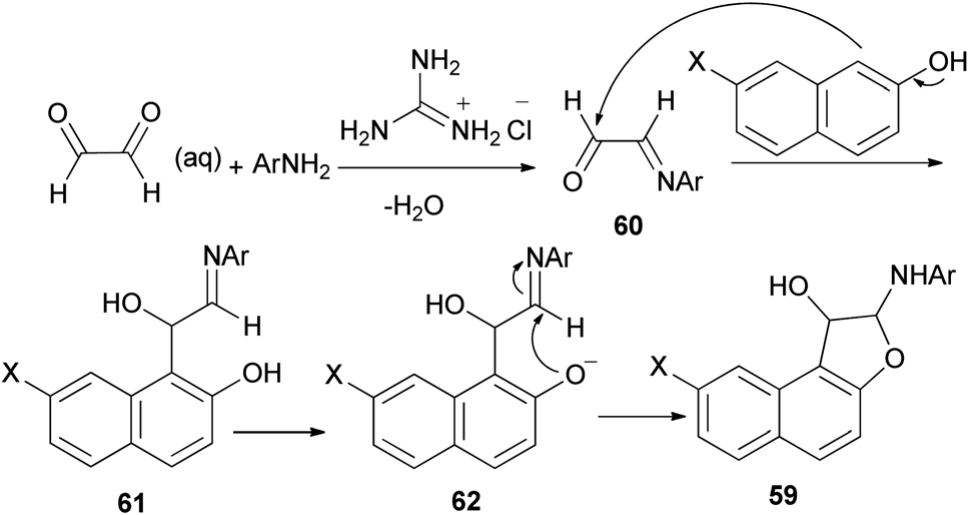
Plausible mechanism for the formation of 59.

A simple and highly efficient one-pot protocol for the synthesis of novel polysubstituted 1,2-dihydronaphtho[2,1-*b*]furans 59 in 87–91% yields has been developed by three-component coupling reaction of 2-aminopyridines, naphthols and aqueous glyoxal in the presence of guanidinium chloride as a polyfunctional organocatalyst under solvent-free conditions at 80 °C for 25–70 min (Scheme 26). A plausible mechanism for this conversion is shown in Scheme 27. The formation of products can be rationalized by the initial formation of intermediate 60*via* condensation of amine with glyoxal. Subsequent addition of 2-naphthol to 60 affords intermediate 61 followed by intramolecular nucleophilic addition of naphthoxy ion 62 generated *in situ* by guanidinium chloride to activated imine group led to the formation of the desired products 59.^39^

**Scheme 28:**
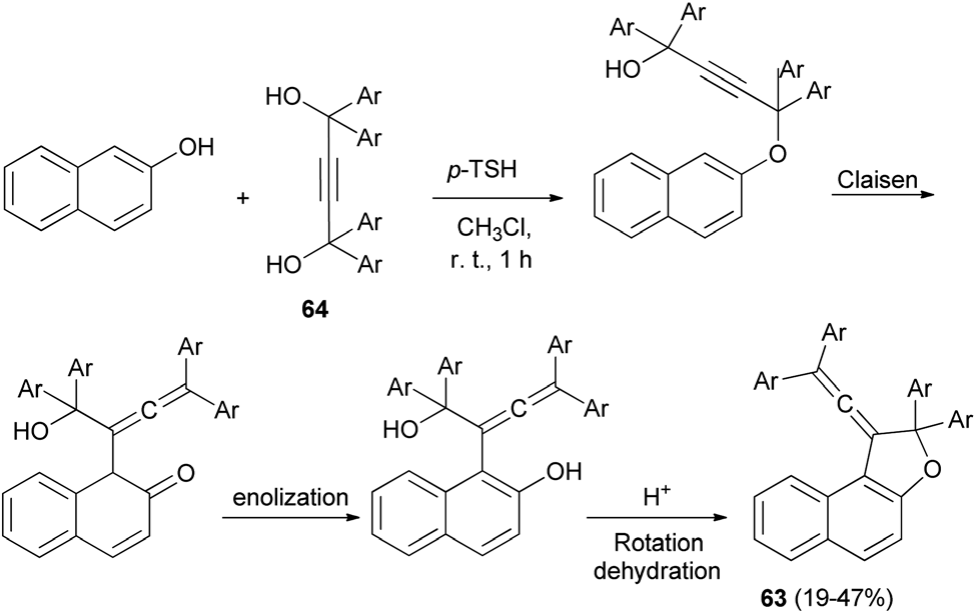
Synthesis of 1-vinylidene-naphthofurans 63.

A set of new 1-vinylidene-1,2-dihydronaphtho[2,1-*b*]furans 63 in 19–47% yields was unexpectedly obtained in the reaction of 2-naphthol with readily available 1,1,4,4-tetraarylbut-2-yne-1,4-diols 64 in the presence of 4-toluenesulfonic acid monohydrate in CHCl_3_ at room temperature for 1 h. This reaction may be assumed to proceed *via* Claisen rearrangement, enolization and rotation dehydration as shown in Scheme 28.^15^

**Scheme 29:**
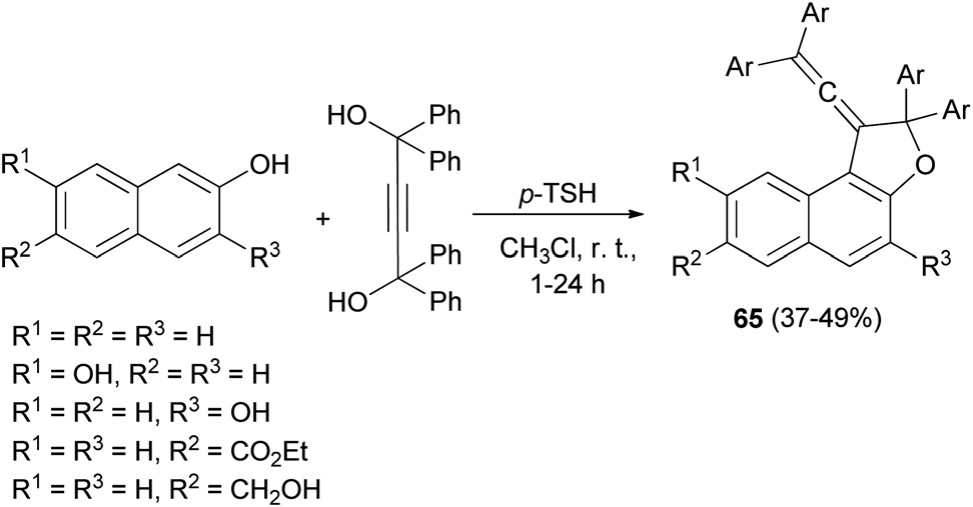
*p*-TSA-catalyzed synthesis of vinylidene-naphthofurans 65.

In a similar fashion, vinylidene-naphthofurans 65 are a new class of photochromic molecules, easily synthesized by reaction of 1,1,4,4-tetraphenylbut-2-yne-1,4-diol with naphthols in the presence of 4-toluenesulfonic acid monohydrate in CHCl_3_ at room temperature for 1–24 h in 37–49% yields (Scheme 29).^16^

**Scheme 30:**
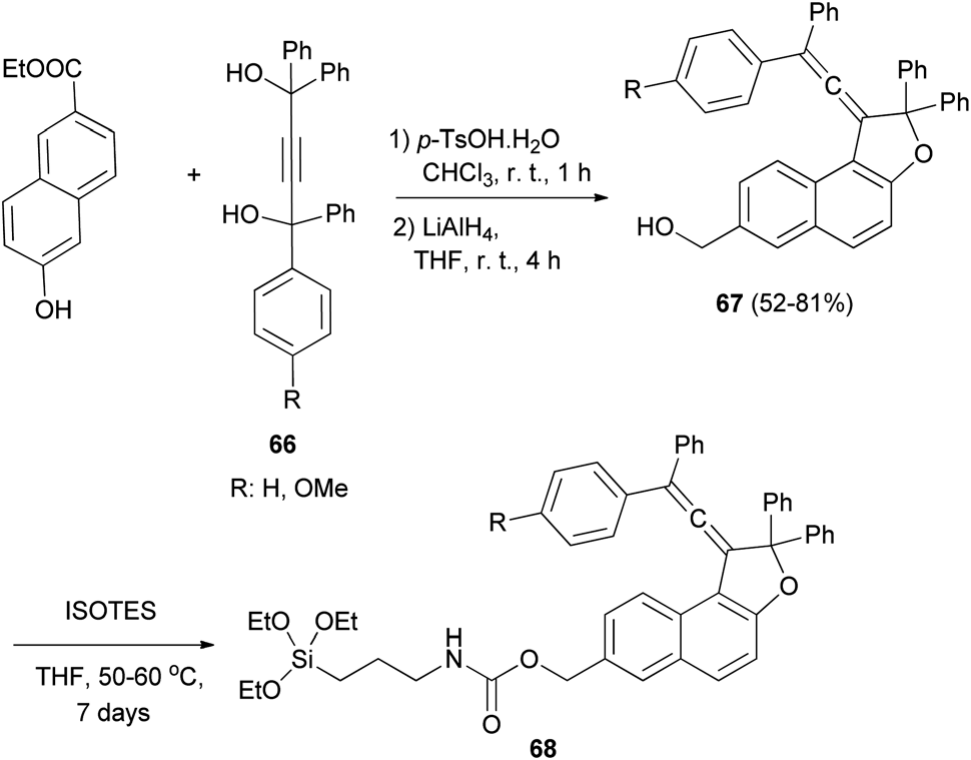
Synthesis of vinylidene-naphthofurans 67 and 68.

Coelho *et al.* observed that the treatment of ethyl 6-hydroxy-2-naphthoate with diols 66 under acid catalysis in CHCl_3_ at room temperature for 1 day, followed by reduction with LiAlH_4_ in THF at room temperature for 4 h afforded the vinylidene-naphthofurans 67 in good yields (52–81%). The later compounds 67 were silanized with 3-(triethoxysilyl) propyl isocyanate (ISOTES) in THF at 50–60 °C for 7 days afforded the respective silanized products 68 as the photoactive molecules (Scheme 30).^19^

**Scheme 31:**
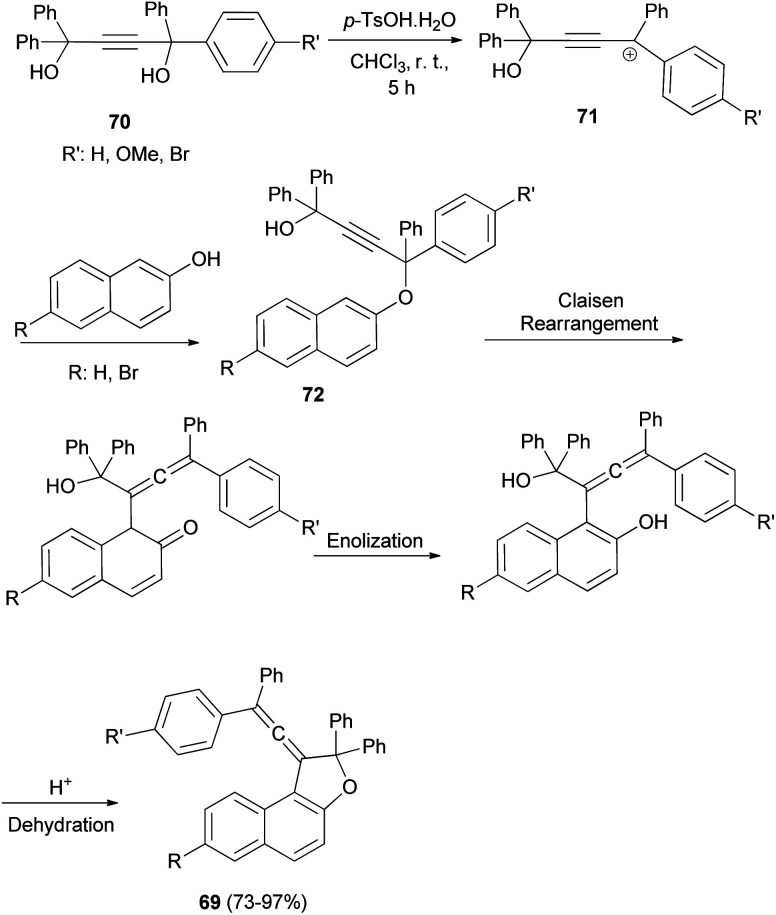
Synthesis of photochromic vinylidene-naphthofurans 69.

In a similar manner, a series of novel photochromic vinylidene-naphthofurans 69 was synthesized by reaction of 2-naphthols with tetraarylbut-2-yne-1,4-diol (70) in the presence of *p*-TSA in CHCl_3_ at room temperature for 5 h in 73–97% yields. This one-pot reaction occurs *via* a domino reaction (Scheme 31): under acid-catalysis the diol 70 is converted into a propargylic carbocation 71, which upon reaction with 2-naphthol provides a propargylic aryl ether 72. Then, this intermediate performs a [3,3]-sigmatropic Claisen rearrangement followed by enolization and acid-catalyzed intramolecular dehydration affording the final vinylidene-naphthofuran 69.^20^

**Scheme 32:**
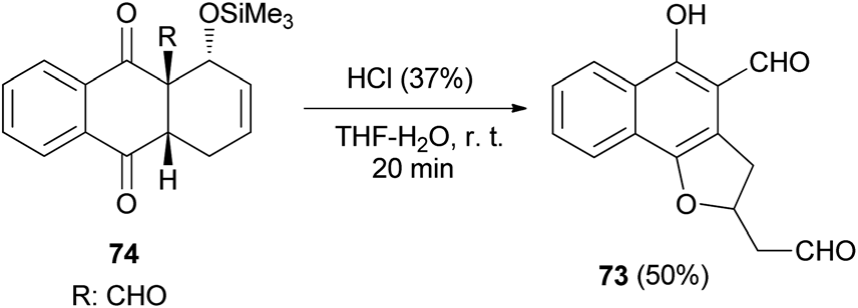
Synthesis of 2,3-dihydronaphtho[1,2-*b*]furan 73.

4-Formyl-5-hydroxy-2-(2′-oxoethyl)2,3-dihydronaphtho[1,2-*b*]furan (73) was readily achieved in 50% yield by acid induced fragmentation of the Diels–Alder adduct 74 in 90% aqueous THF at room temperature for 20 min (Scheme 32).^40^

**Scheme 33:**
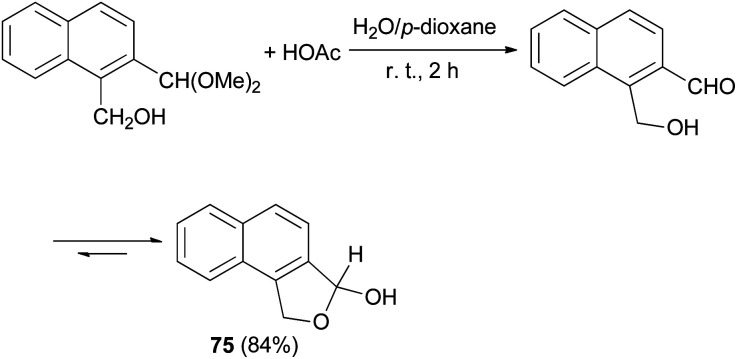
Preparation of dihydronaphthofuran 75.

Smith *et al.*^32^ synthesized 1,3-dihydro-3-hydroxynaphtho[1,2-*c*]furan (75) in 84% yield from the reaction of 1-(hydroxymethy1)-2-(dimethoxymethy1)naphthalene with HOAc in water and *p*-dioxane at room temperature for 2 h (Scheme 33).

**Scheme 34:**
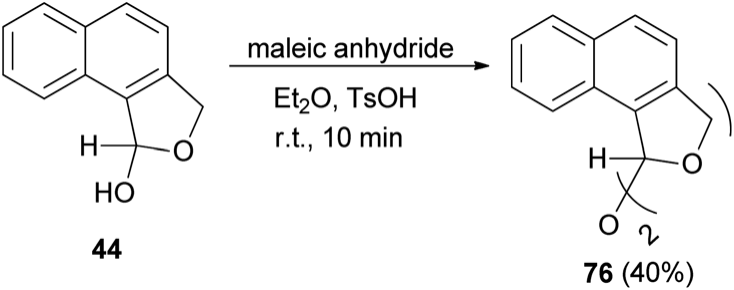
Preparation of bis-dihydronaphthofuran 76.

Also, bis(1,3-dihydro-*l*-naphthol[1,2-*c*]furanyl)ether (76) was obtained in 40% yield by the reaction of 44 with maleic anhydride in diethyl ether as a solvent using a catalytic amount of TsOH at room temperature for 10 min (Scheme 34).^32^

### 2.3. Transition-metal catalyzed synthesis

**Scheme 35:**
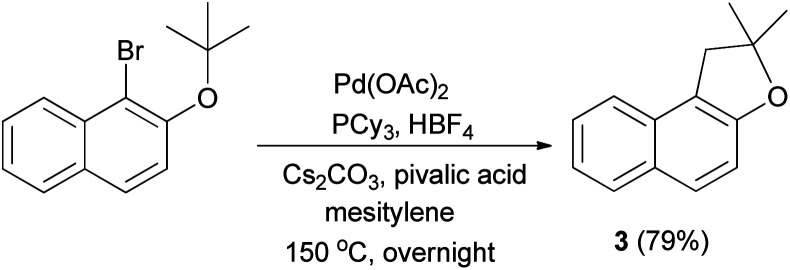
Pd(OAc)_2_ catalyzed synthesis of dihydronaphthofuran 3.

In 2007, Lafrance *et al.*^41^ have obtained dihydronaphthofuran 3 in 79% yield *via* the reaction of 1-bromo-2-(*tert*-butoxy)naphthalene in the presence of Pd(OAc)_2_ (5 mol%), PCy_3_, HBF_4_ (6 mol%) in conjunction with Cs_2_CO_3_ as base and 2,2-dimethylpropionic acid (pivalic acid, 30 mol%) as additive in mesitylene at 150 °C for overnight (Scheme 35).

**Scheme 36:**
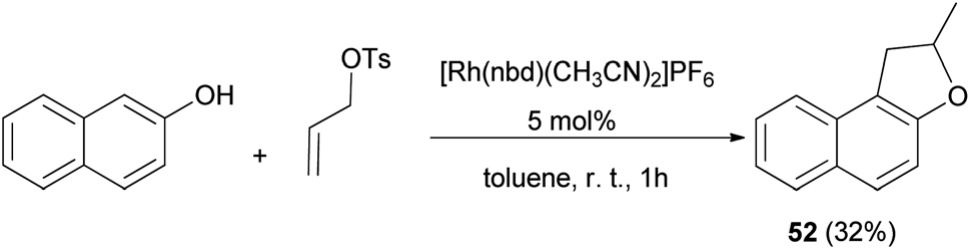
[Rh(nbd)(CH_3_CN)_2_]PF_6_ catalyzed synthesis of 1,2-dihydronaphthofuran 52.

A simple procedure for the preparation of 2-methyl-1,2-dihydronaphtho[2,1-*b*]furan (52) in 32% yield by allylation of 2-naphthol with allyl tosylate in the presence of [Rh(nbd)(CH_3_CN)_2_]PF_6_ as catalyst in toluene at room temperature for 1 h was described by Tsukada *et al.* (Scheme 36).^42^

**Scheme 37:**
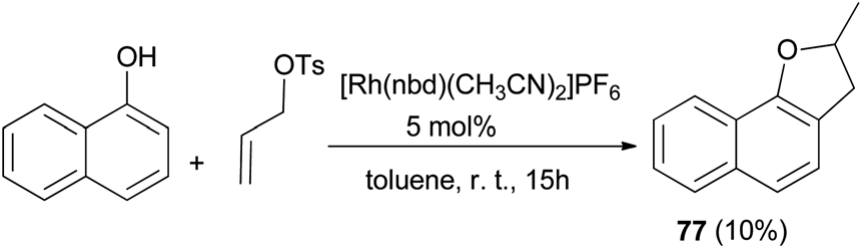
Transition-metal catalyzed synthesis of 2-methyl-2,3-dihydronaphtho[1,2-*b*]furan (77).

Similarly, 2-methyl-2,3-dihydronaphtho[1,2-*b*]furan (77) in 10% yield was obtained *via* the above reaction condition (Scheme 37).^42^

**Scheme 38:**
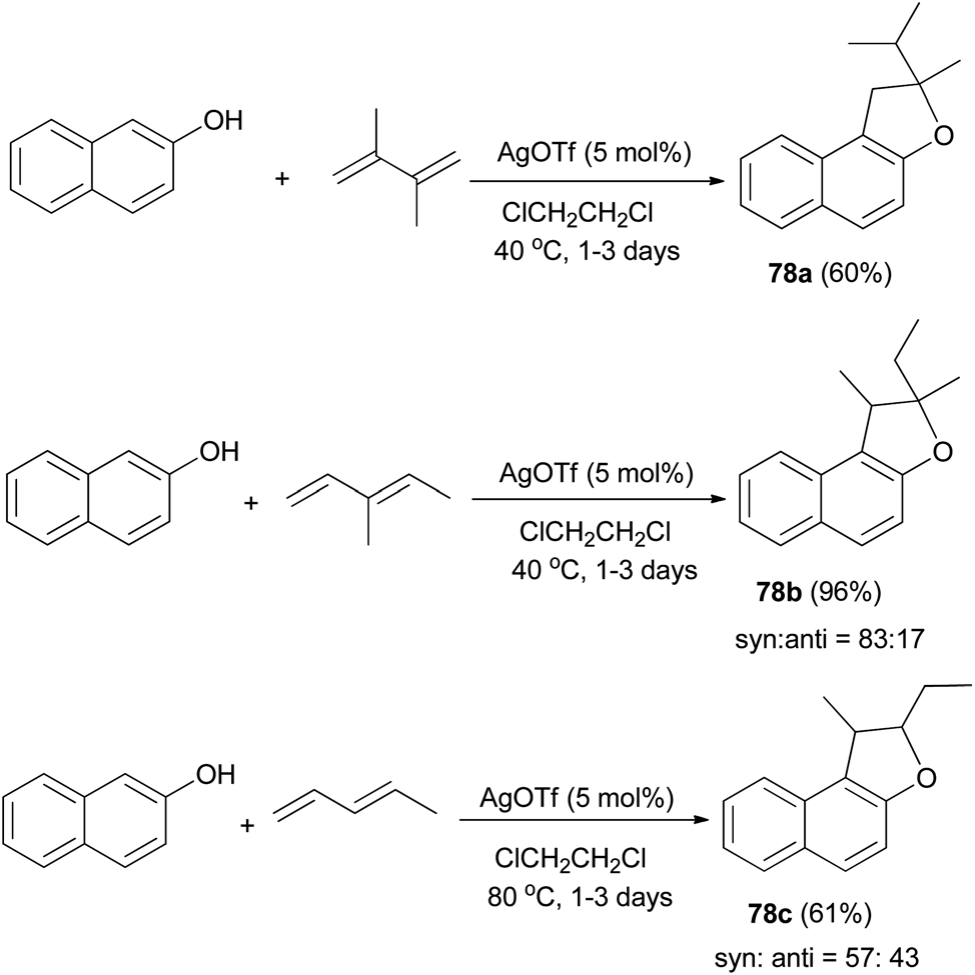
AgOTf-catalyzed synthesis of dihydronaphthofurans 78.

**Scheme 39:**
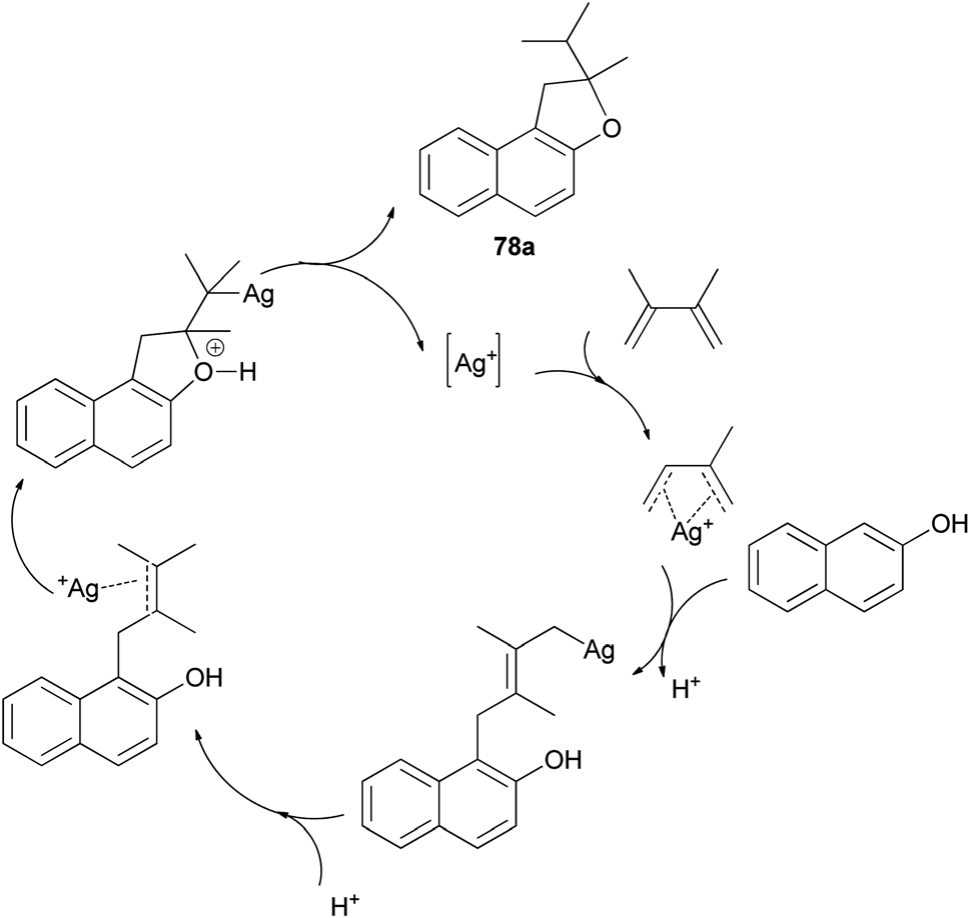
Plausible mechanistic pathway for the formation of 78a.

Mild, efficient, and economical Ag(i)-catalyzed sequential C–C/C–O bond formations between 2-naphthol and dienes in dichloroethane at 40 and 80 °C for 1–3 days were developed to afford a variety of 1,2-dihydronaphtho[2,1-*b*]furans 78a–c in 60–96% yields (Scheme 38). A plausible mechanism for formation of products 78a is shown in Scheme 39. Activation of the diene by coordination to Ag(i) is followed by intermolecular nucleophilic attack by the arene. The reactions of 2-naphthol with 1-substituted-1,3-diene substrates occur by either 1,2- or 1,4-addition. The resulting Ag–C bond is protonated to give 2-allylphenol intermediate then recoordination of C–C π-bond by Ag(i) activates the olefin toward intramolecular nucleophilic attack by the phenolic oxygen. Subsequent proton transfer produces the final product and regenerates the Ag(i) catalyst.^43^

**Scheme 40:**
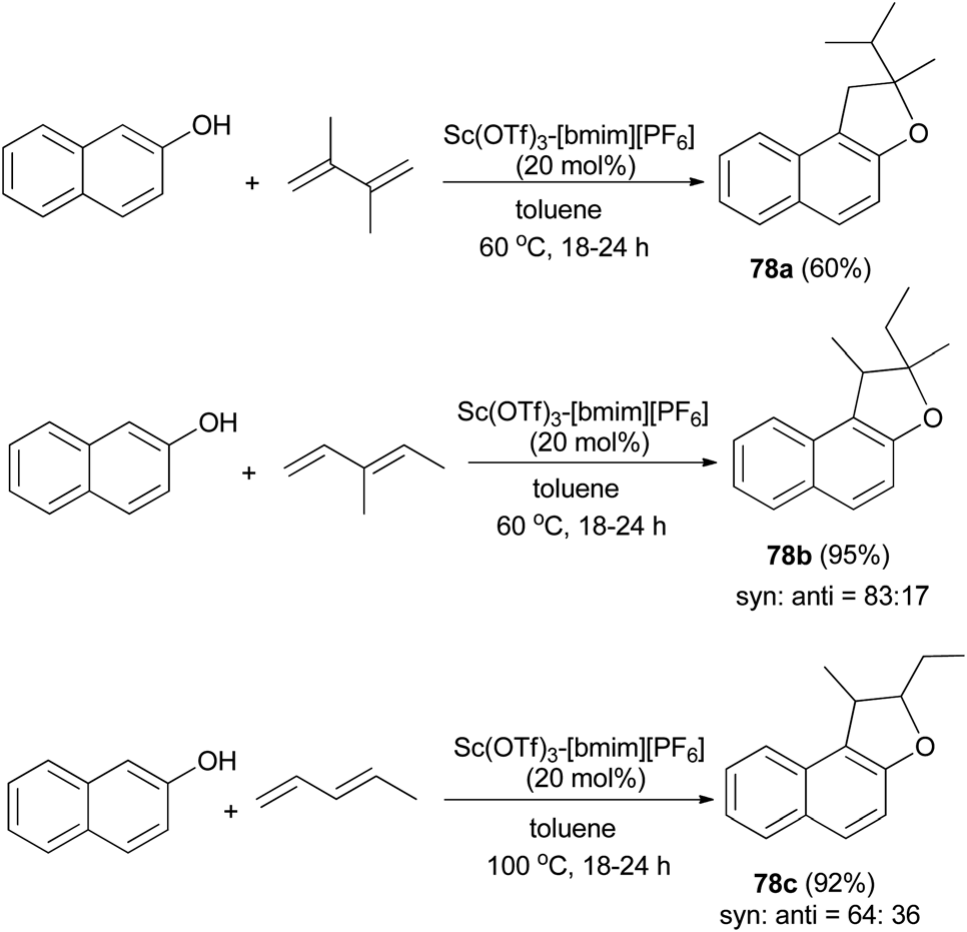
Sc(OTf)_3_-[bmim][PF_6_]-catalyzed synthesis of dihydronaphthofurans 78.

In a similar fashion, reusable scandium/ionic liquid catalyst system for sequential C–C and C–O bond formations between 2-naphthol and dienes with atom economy has been reported for the synthesis of 78a–c in toluene at 60 and 100 °C for 18–24 h in 60–95% yields (Scheme 40).^44^

**Scheme 41:**
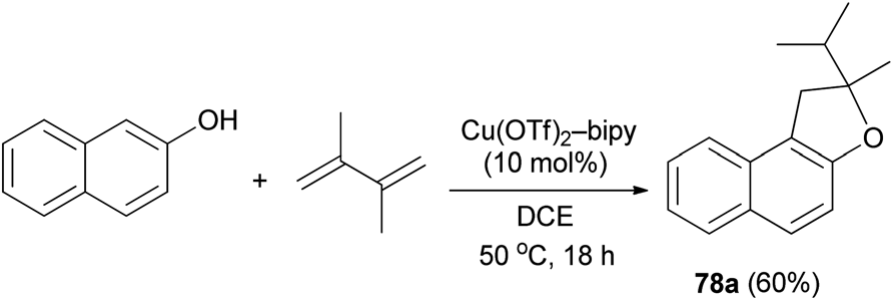
Cu(OTf)_2_-bipy catalyzed synthesis of dihydronaphthofuran 78a.

It was also shown that air-and moisture-stable Cu(OTf)_2_-bipy catalyze the addition of 2-naphthol to 1,3-diene in dichloroethane at 50 °C for 18 h in a tandem hydroalkoxylation-rearrangement-hydroalkylation sequence, furnishing *O*-heterocycles such as 2-isopropyl-2-methyl-1,2-dihydronaphtho[2,1-*b*]furan (78a) in 60% yield (Scheme 41).^45^

**Scheme 42:**
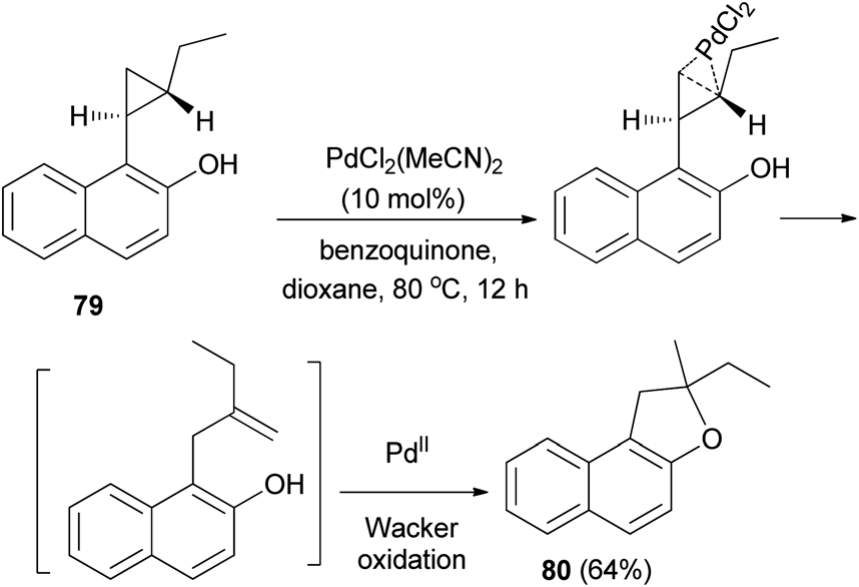
PdCl_2_-catalyzed synthesis of dihydronaphthofuran 80.

Palladium chloride-catalyzed intramolecular activation of electroneutral arylcyclopropane 79 in the presence of benzoquinone as oxidant in dioxane at 80 °C for 12 h resulted 2-ethyl-2-methyl-1,2-dihydronaphtho[2,1-*b*]furan (80) in 64% yield *via* cyclopropane isomerization followed by a Wacker oxidation (Scheme 42).^46^

**Scheme 43:**
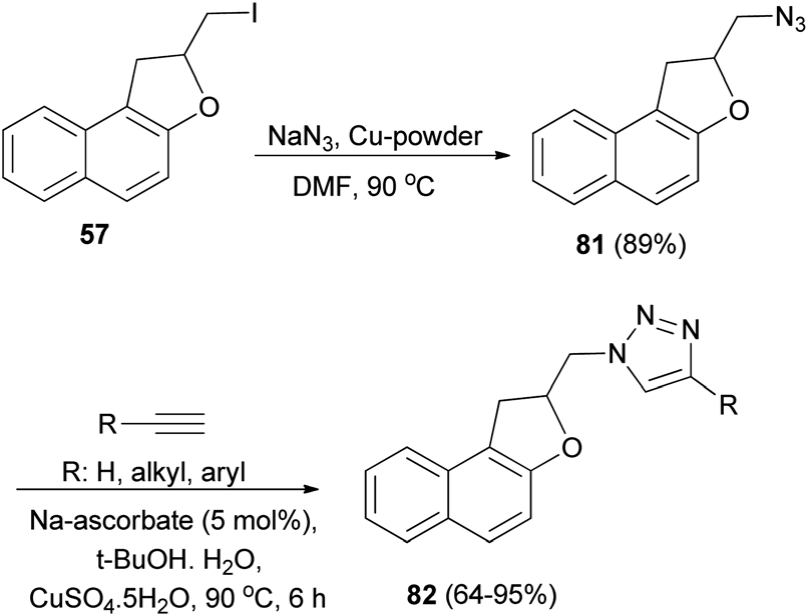
Preparation of dihydronaphthofurans 81 and 82.

Tripathi *et al.*^9^ investigated reaction of dihydronaphthofuran 57 with NaN_3_ in the presence of Cu-powder in DMF at 80 °C gave 2-(azidomethyl)-1,2-dihydronaphtho[2,1-*b*]furan (81) in 89% yield. [3 + 2] Cycloaddition of 81 with different alkynes in the presence of sodium ascorbate and CuSO_4_·5H_2_O (1 mol%) in 2 : 1 mixture of *tert*-butyl alcohol and water at 90 °C for 6 h afforded dihydronaphthofurans 82 in 64–95% yields (Scheme 43).

**Scheme 44:**
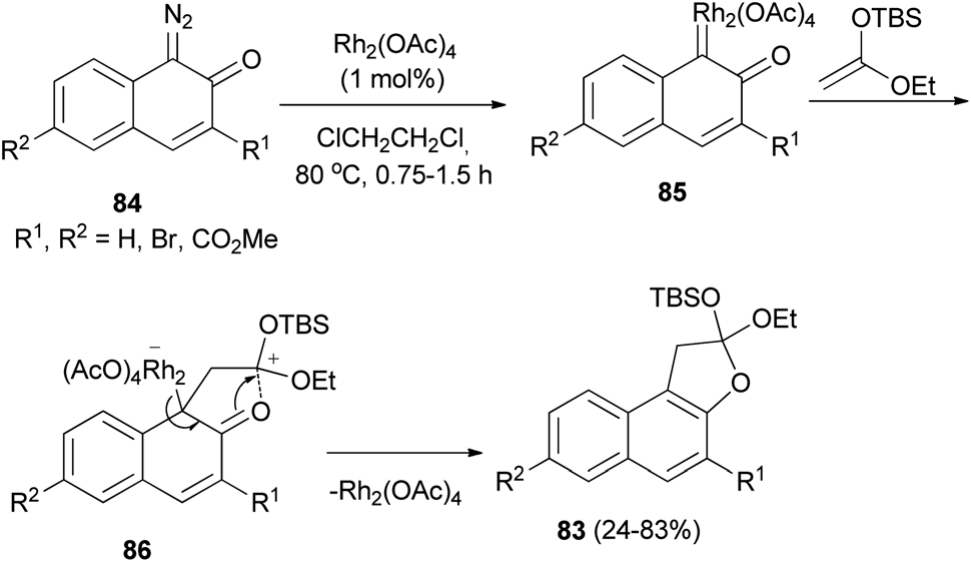
Rhodium-catalyzed synthesis of dihydronaphthofurans 83.

Kitamura *et al.*^47^ developed Rh_2_(OAc)_4_-catalyzed synthesis of dihydronaphthofurans 83 in 24–83% yields by the cycloaddition reaction of diazonaphthoquinones 84 with enol ethers in dichloroethane at 80 °C for 0.75–1.5 h. A possible reaction mechanism is illustrated in (Scheme 44). Initially, Rh_2_(OAc)_4_ reacts with 84 to form Rh^II^ carbene complex 85. Nucleophilic attack by the enol ether on carbene complex 85 proceeds to form 86. Then, aromatization drives the cyclization to form naphthofuran 83 with release of Rh_2_(OAc)_4_.

**Scheme 45:**
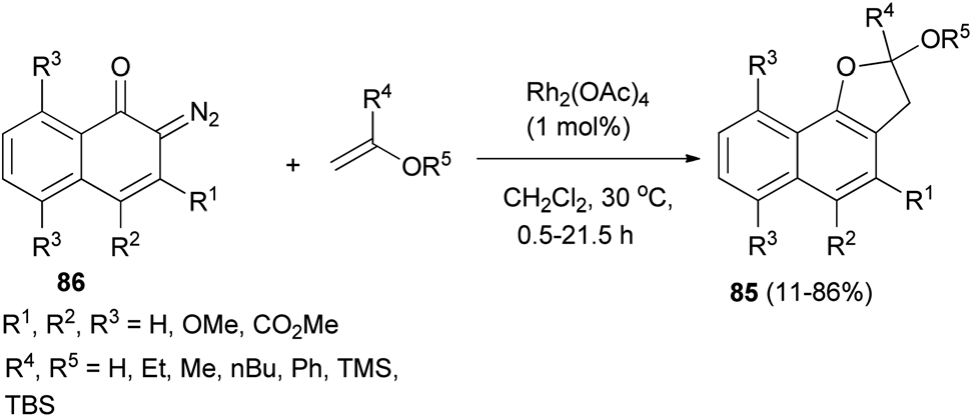
Rhodium-catalyzed synthesis of 2,3-dihydronaphtho[1,2-*b*]furan derivatives 85.

In a similar fashion, synthesis of 2,3-dihydronaphtho[1,2-*b*]furan derivatives 85 in 11–86% yields has been achieved by the cycloaddition reaction of diazonaphthoquinones 86 with enol ethers in CH_2_Cl_2_ at 30 °C for 0.5–21.5 h (Scheme 45).^47^

**Scheme 46:**
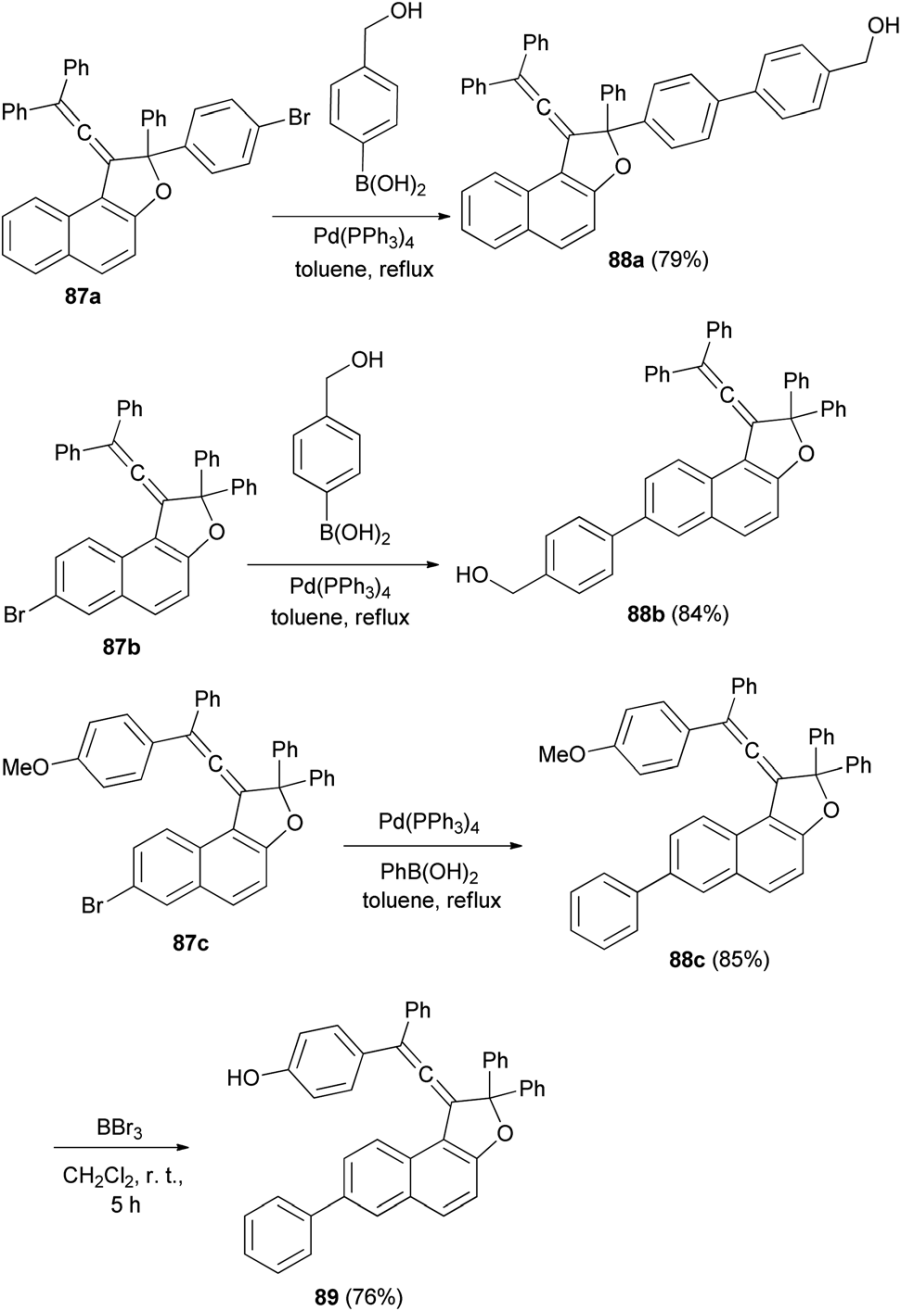
Synthesis of photochromic vinylidene-naphthofurans 88 and 89.

Reaction of vinylidene-naphthofuran derivatives 87a–c with boronic acid in the presence of Pd(PPh_3_)_4_ and K_2_CO_3_ in refluxing toluene for 4–5 h afforded photochromic vinylidene-naphthofurans 88a–c in 79–85% yields. The reaction of 88c with BBr_3_ in dry CH_2_Cl_2_ at room temperature for 5 h afforded photochromic vinylidene-naphthofuran 89 in 76% yield (Scheme 46).^20^

**Scheme 47:**
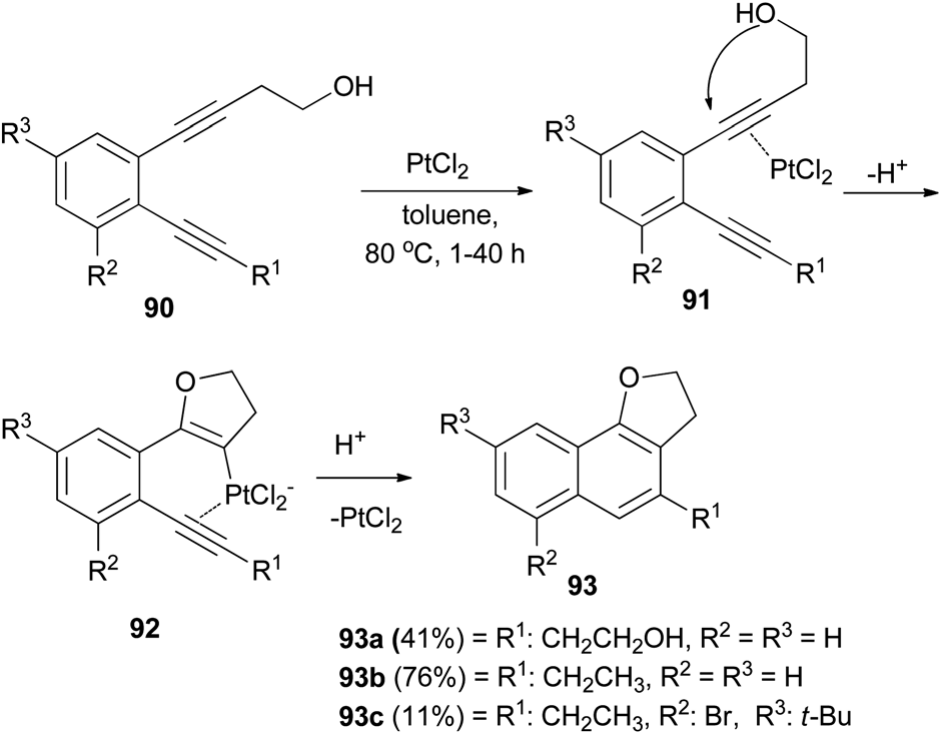
PtCl_2_-catalyzed cyclization of *o*-diethynylbenzenes to dihydronaphthofurans 93.

PtCl_2_-catalyzed cyclization of *o*-diethynylbenzene derivatives 90 bearing a hydroxyethyl group in toluene under an argon atmosphere at 80 °C for 1–40 h afforded dihydronaphthofuran derivatives 93a–c in 11–76% yields. Formation of 93 can be rationalized by initial intramolecular cyclization of the hydroxy group to an activated ethynyl group in 91 to form intermediate 92, followed by attack of the second ethynyl group to give 93 (Scheme 47).^48^

**Scheme 48:**
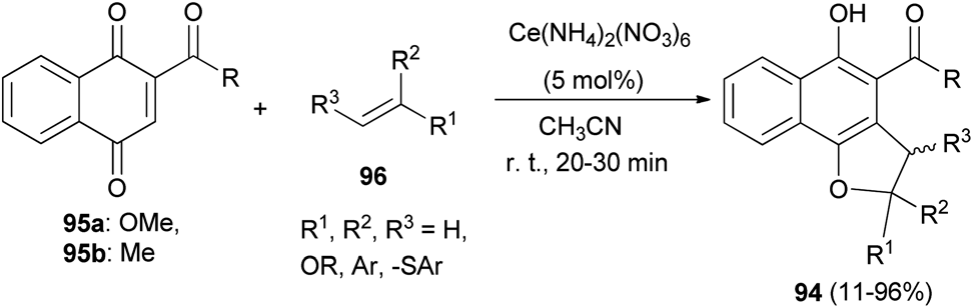
Ceric ammonium nitrate-catalyzed synthesis of dihydronaphtho[1,2-*b*]furans 94.

**Scheme 49:**
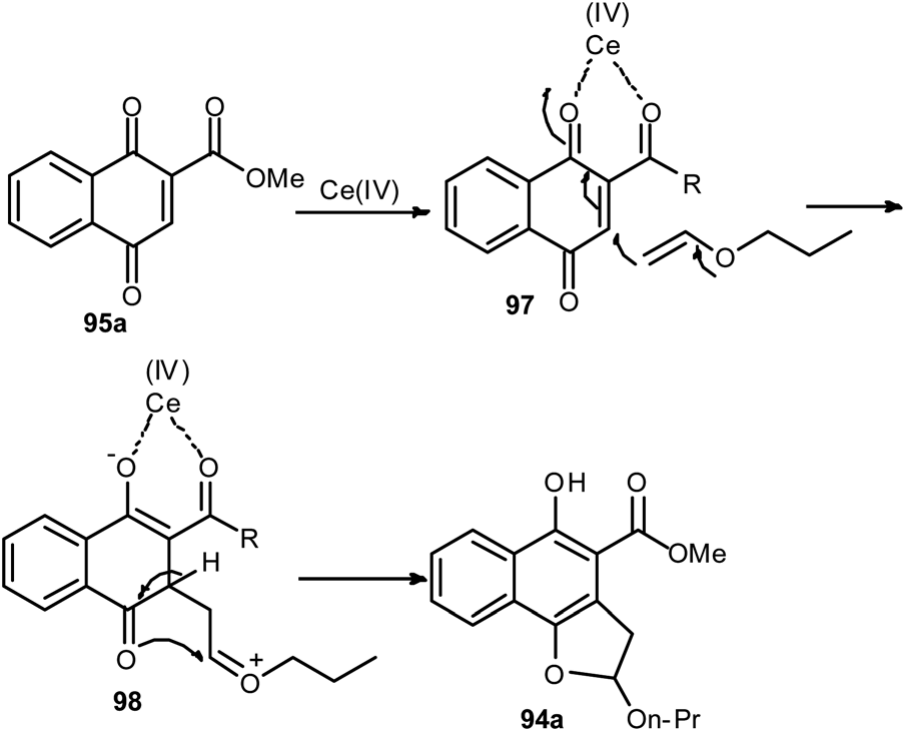
Plausible mechanistic pathway for formation of 94.

Lee and Xia described a novel approach for the synthesis of diverse dihydronaphtho[1,2-*b*]furans 94 in 11–96% yields from 1,4-naphthoquinones 95 and olefins 96 in the presence of ceric ammonium nitrate in CH_3_CN at room temperature for 20–30 min *via* formal [3 + 2] cycloaddition reaction (Scheme 48). This methodology was also used successfully to synthesize the biologically important natural product furomollugin in only 2 steps. The formation of 94 can be explained by the mechanism proposed in Scheme 49. The methyl 1,4-dioxo-1,4-dihydronaphthalene-2-carboxylate (95a) first forms complex 97 in the presence of CAN. The vinyl group of ethyl vinyl ether then attacks 97 to give another intermediate 98. Isomerization of 98 followed by intramolecular cyclization then gives the final product 94a.^49^

**Scheme 50:**
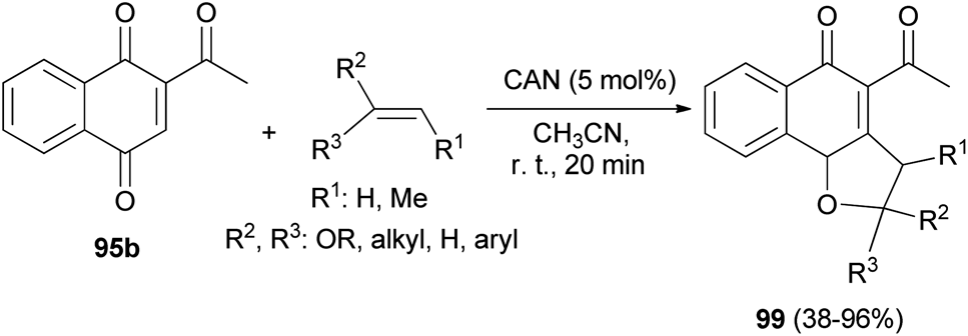
Ammonium cerium(iv) ammonium nitrate-catalyzed synthesis of 2,3-dihydronaphtho[1,2-*b*]furans 99.

Similarly, 2,3-dihydronaphtho[1,2-*b*]furans 99 were synthesized in 38–96% yields by the reaction of 2-acetylnaphthalene-1,4-dione (95b) with olefins in the presence of cerium(iv) ammonium nitrate (CAN, 5.0 mol%) as catalyst in CH_3_CN at room temperature for 20 min (Scheme 50).^10^

**Scheme 51:**
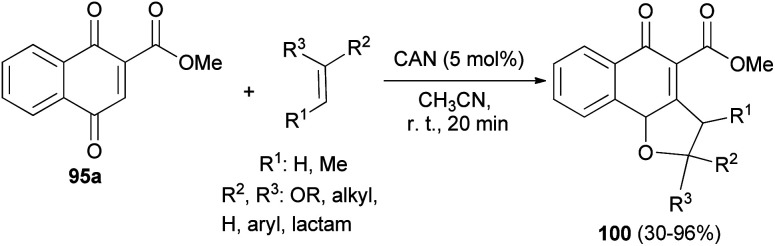
CAN-catalyzed synthesis of 2,3-dihydronaphtho[1,2-*b*]furan derivatives 100.

Also, Lee *et al.*^50^ have described the reaction of 1,4-dihydroxynaphthalene-2-carboxylate 95a and the corresponding olefins in MeCN in the presence of cerium(iv) ammonium nitrate (5.0 mol%) at room temperature for 20 min *via* [3 + 2] cycloaddition reaction afforded dihydronaphthofuran derivatives 100 in 30–96% yields (Scheme 51).

**Scheme 52:**
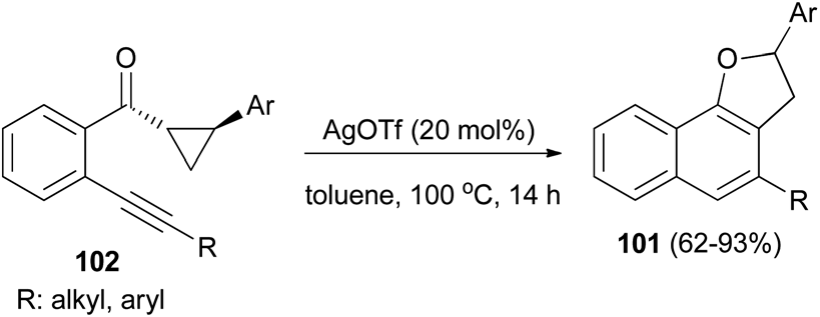
AgOTf-catalyzed synthesis of 2,3-dihydronaphtho[1,2-*b*]furans 101.

**Scheme 53:**
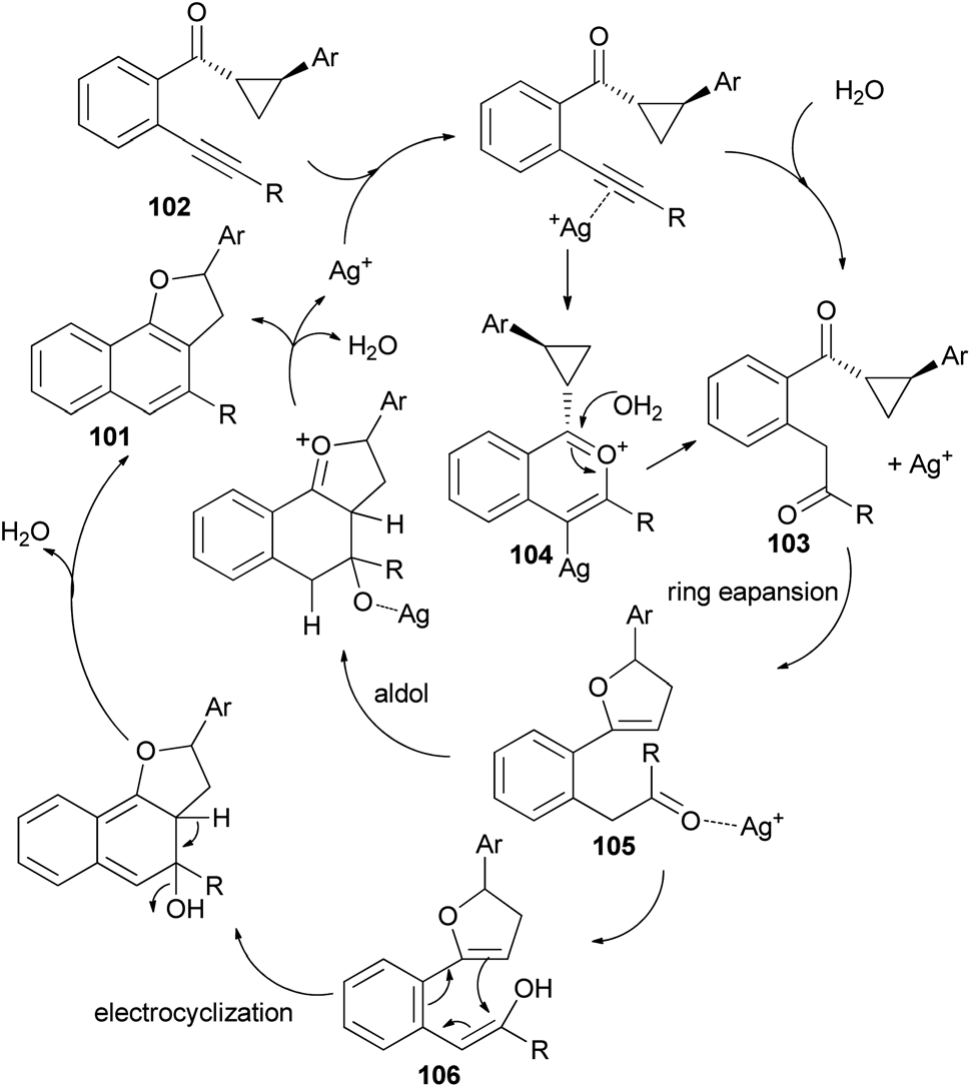
Proposed mechanism of the formation of 101.

Recently Katukojvala *et al.* found that 2,3-dihydronaphtho[1,2-*b*]furans 101 could be prepared in 62–93% yields from intramolecular transannulation of ((2-alkynyl)aryl)cyclopropyl ketones 102 in the presence of AgOTf (20 mol%) as catalyst in toluene at 100 °C for 14 h (Scheme 52). As shown in Scheme 53, a plausible mechanism for the transannulation reaction was proposed. The first step involves Ag(i)-catalyzed, neighbouring carbonyl group directed regioselective hydration of the alkyne moiety of 102*via* the benzo[*c*]-pyrylium cation 104 to give the intermediate diketone 103. In the next step, Ag(i)-catalyzed regioselective ring expansion of the donor–acceptor cyclopropyl ring of 103 leads to the transient 2,3-dihydrofuran 105. Subsequent intramolecular benzannulation of 105 *via* either aldol condensation or 6π-electrocyclization of trienol 106 leads to the 2,3-dihydronaphtho[1,2-*b*]furan 101.^51^

**Scheme 54:**
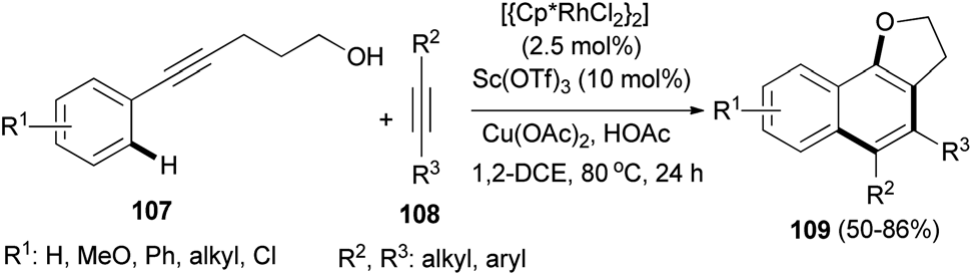
Sc(OTf)_3_ and rhodium catalyzed synthesis of 2,3-dihydronaphtho[1,2-*b*]furans 109.

The cascade reaction of alkynols 107 with alkynes 108 under combined Sc(OTf)_3_ and rhodium catalyst, Cu(OAc)_2_ and HOAc in dichloroethane under nitrogen atmosphere at 80 °C for 24 h led to the formation of 2,3-dihydronaphtho[1,2-*b*]furans 109 in 50–86% yields (Scheme 54).^52^

**Scheme 55:**
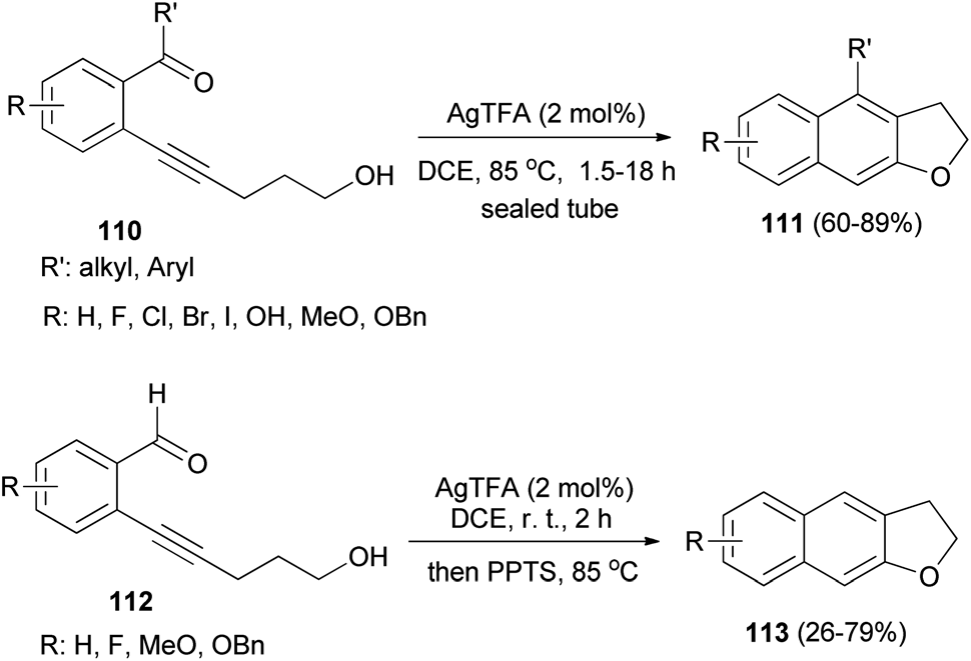
AgTFA-catalyzed synthesis of 2,3-dihydronaphtho[2,3-*b*]furans 111 and 113.

*ortho*-Carbonylarylacetylenols 110 have been employed for the synthesis of 2,3-dihydronaphtho[2,3-*b*]furans 111 in 60–89% yields *via* AgTFA (2 mol%) catalyzed annulation reaction in DCE at 85 °C for 1.5–18 h. In a similar fashion, annulation of *ortho*-formylarylacetylenol 112 in the presence of AgTFA and PPTS gave 2,3-dihydronaphtho[2,3-*b*]furans 113 in 26–79% yields (Scheme 55).^53^

**Scheme 56:**
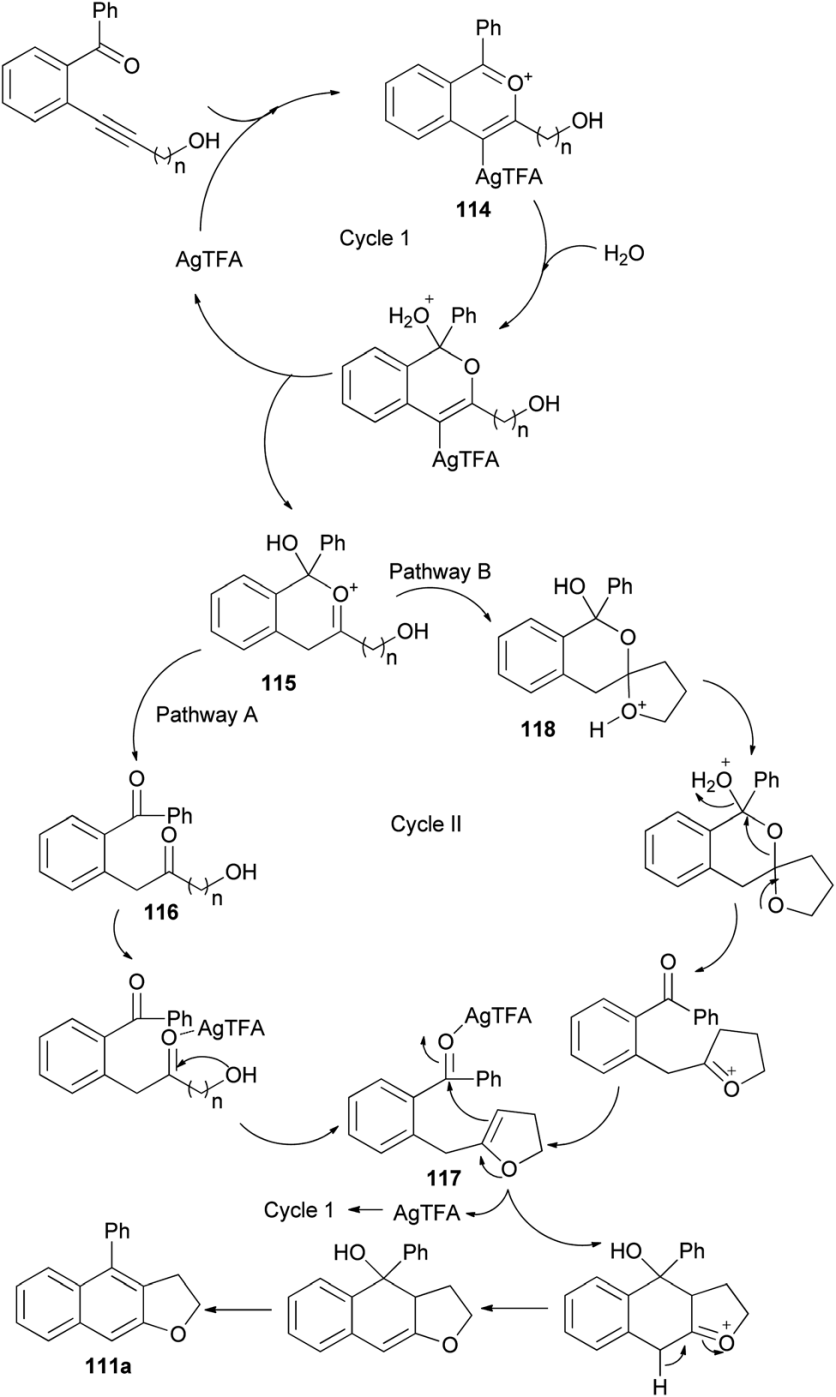
Proposed reaction mechanism for the synthesis of 111a.

The reaction mechanism of this transformation was then proposed as shown in pathway A of Scheme 56. First, alkyne was activated by Ag-catalyst with the assistance of *ortho*-carbonyl neighboring group to form oxo-carbenium ion intermediate 114. After downstream process, this intermediate was then converted to intermediate 115 which further collapsed to give 1,5-diketone 116. Under the presence of mild Ag-Lewis acid, this intermediate was activated and underwent the cyclization to obtain dihydrofuran intermediate 117. Next, the second cyclization proceeded followed by tautomerization and dehydration providing 2,3-dihydronaphtho[2,3-*b*]furan product 111a. Moreover, the mechanism in pathway B is also possible and cannot be ruled out. In pathway B, intermediate 115 underwent the cyclization to give spiro acetal 118 which could be converted to dihydrofuran intermediate 117. This intermediate could also be transformed to the desired product *via* the reaction mechanism in pathway A.^53^

### 2.4. Iodine-assisted synthesis

**Scheme 57:**
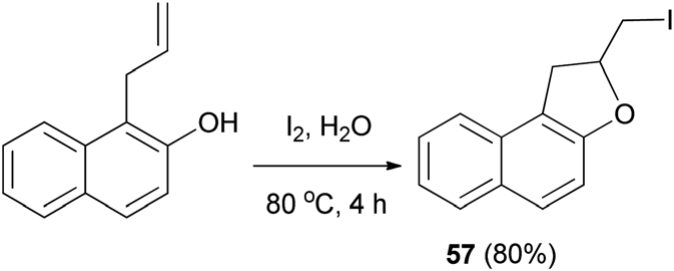
Synthesis of 2-(iodomethyl)-1,2-dihydronaphtho[2,1-*b*]furan (57).

Tripathi *et al.*^54^ demonstrated that synthesis of 2-(iodomethyl)-1,2-dihydronaphtho[2,1-*b*]furan (57) in 80% yield by reaction of 1-allyl-2-naphthol with iodine in water at 80 °C for 4 h (Scheme 57).

**Scheme 58:**
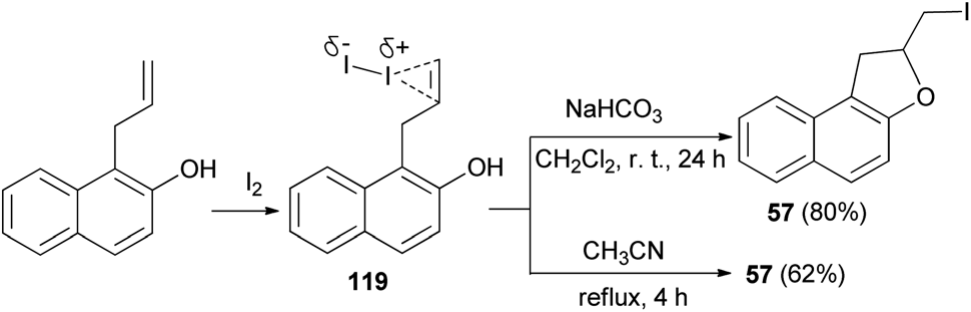
Synthesis of dihydronaphthofuran 57 in the presence of I_2_.

Similarly, an efficient method was developed for the synthesis of dihydronaphthofuran 57 in 80% yield by the treatment of allyl naphthol with molecular iodine in the presence of NaHCO_3_ in CH_2_Cl_2_ at room temperature for 24 h. The key step involve iodocyclization. In a similar manner, *ortho*-allylnaphthol on reaction with molecular iodine in CH_3_CN under reflux condition for 4 h gave dihydronaphthofuran 57 in 62% yield (Scheme 58). This reaction is assisted by the hydroxyl group, involves formation of iodonium ion 119 and proceeds through non radical mechanism pathway.^55^

**Scheme 59:**
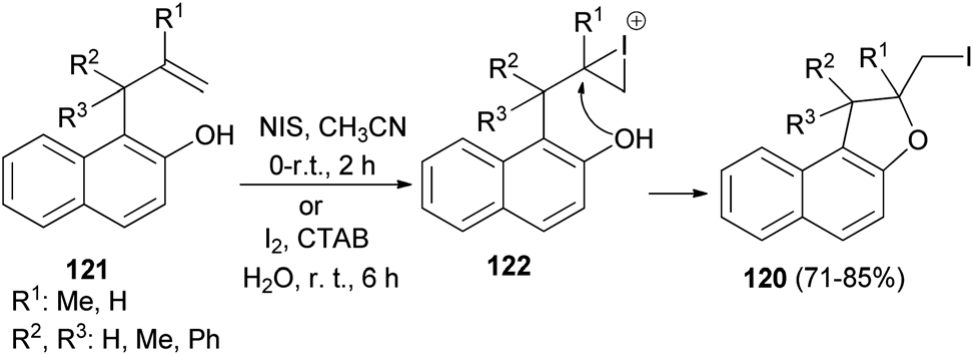
Synthesis of dihydronaphthofurans 120.

A number of 2-(iodomethyl)-1,2-dihydronaphtho[2,1-*b*]furan derivatives 120 in 71–85% yields have been synthesized from the corresponding allylhydroxy naphthalene precursors 121 involving *N*-iodosuccinimide in acetonitrile at 0–5 °C and then at room temperature for 2 h or by employing molecular iodine in aqueous micelle using CTAB as surfactant at 0–5 °C and then at room temperature for 6 h. At first the allylhydroxy precursor 121 may generate the iodonium intermediate 122, which undergoes 5-*exo*-trig cyclization to from the cyclized product 120 (Scheme 59).^56^

**Scheme 60:**
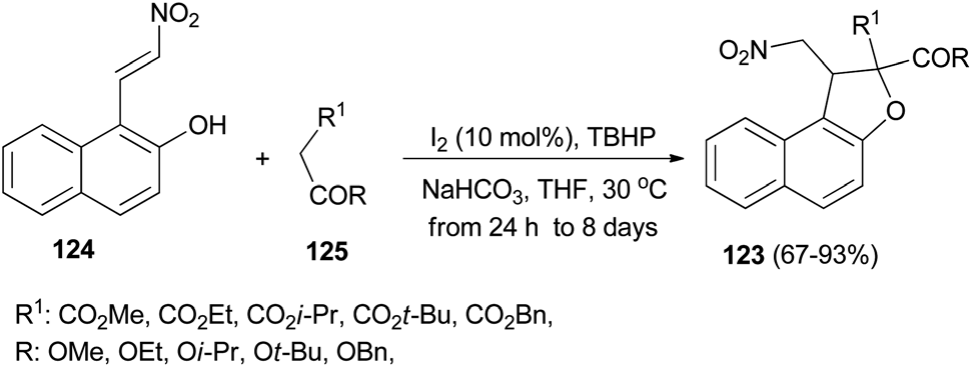
Iodine-catalyzed synthesis of dihydronaphthofurans 123.

**Scheme 61:**
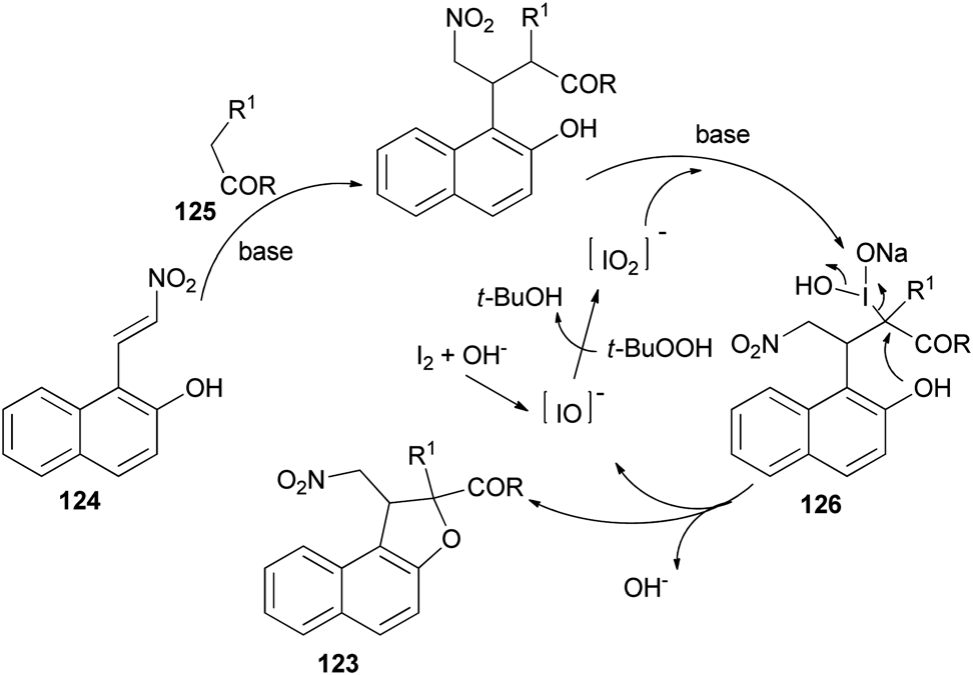
Tentative mechanism for the formation of 123.

Recently Deng *et al.*^57^ found that dihydronaphthofurans 123 in 67–93% yields could be prepared from readily available 1-(2-nitrovinyl)naphthalen-2-ol 124 and malonate esters 125 in the presence of I_2_ (10 mol%), NaHCO_3_ and TBHP as oxidant in THF at 30 °C for 24 h to 8 days (Scheme 60). A tentative mechanism is proposed in Scheme 61. As depicted in Scheme 61, I_2_ could be transformed into hypoiodite [IO]^−^ under basic conditions initially, then, which is further oxidized by TBHP to form the iodite [IO_2_]^−^. The Michael adduct formed by the reaction of malonate ester 125 and nitroalkene 124 could possibly react with the *in situ* formed reactive species [IO_2_]^−^ to give the intermediate 126, which could be captured by intramolecular phenolic hydroxyl to construct dihydronaphthofuran 123 and releasing hypoiodite species [IO]^−^ for the next catalytic cycle.

**Scheme 62:**
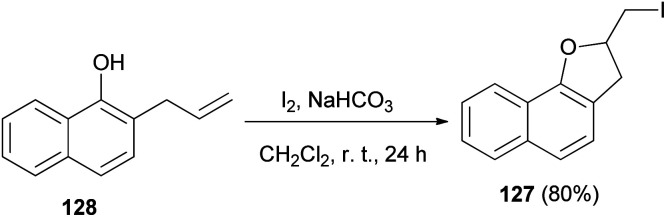
Synthesis of 2-(iodomethyl)-2,3-dihydronaphtho[1,2-*b*]furan (127) in the presence of I_2_.

An efficient method was developed for the synthesis of 2-(iodomethyl)-2,3-dihydronaphtho[1,2-*b*]furan (127) in 80% yield by the reaction of 2-allyl-1-naphthol 128 with molecular iodine in the presence of NaHCO_3_ in CH_2_Cl_2_ at room temperature for 24 h (Scheme 62).^58^

**Scheme 63:**
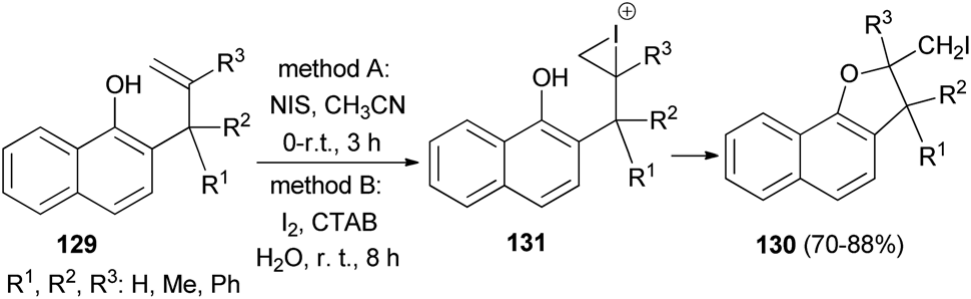
Regioselective 5-*exo*-trig heterocyclization of 2-allyl-1-naphthols 129 to dihydronaphthofurans 130.

Regioselective iodocyclization of a series of allylhydroxy naphthalene precursors 129 involving *N*-iodosuccinimide (method A) and environment friendly green approach associated with surfactant (CTAB)-promoted molecular-iodine-mediated (method B) 5-*exo*-trig cyclization strategies has been explored. Method A: the reaction mixture in CH_3_CN was magnetically stirred for 150 minutes at 0–5 °C and then at room temperature for further 30 minutes afforded dihydronaphthofurans 130 in 70–76% yields. Method B: the reaction mixture in water was stirred at room temperature for an additional period of 8 h gave dihydronaphthofurans 130 in 80–88% yields. The transformation is believed to proceed *via* the iodonium intermediate 131 (Scheme 63).^59^

### 2.5. Syntheses *via* phosphorus, nitrogen and sulfur ylides

**Scheme 64:**
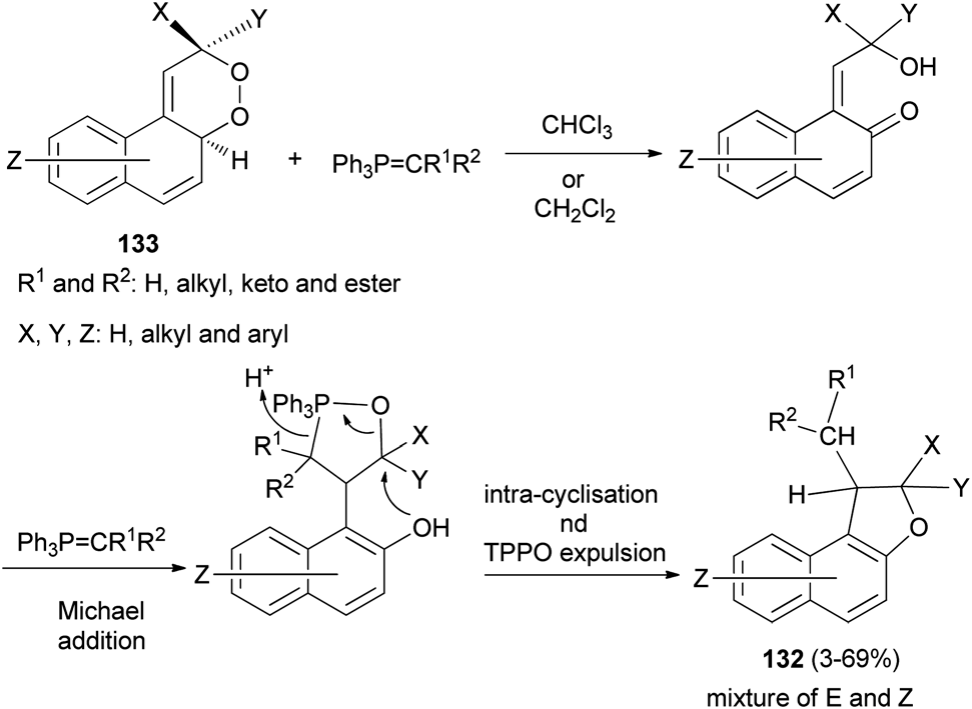
Synthesis of 1,2-dihydronaphtho[2,1-*b*]furans 132.

Taylor *et al.*^60^ described practical synthesis of functionalized 1,2-dihydronaphtho[2,1-*b*]furans 132 from substituted 2,4*a*-dihydronaphtho[2,1-*c*][l,2]dioxines 133 and stabilised phosphorus ylides. Interaction of 133 in anhydrous chloroform or dichloromethane under a nitrogen gas atmosphere with ylide at a temperature of 60 °C or ambient temperature for 3–7 days resulted in the formation of *E* and *Z* isomers of 132 in 3–69% yields (Scheme 64).

**Scheme 65:**
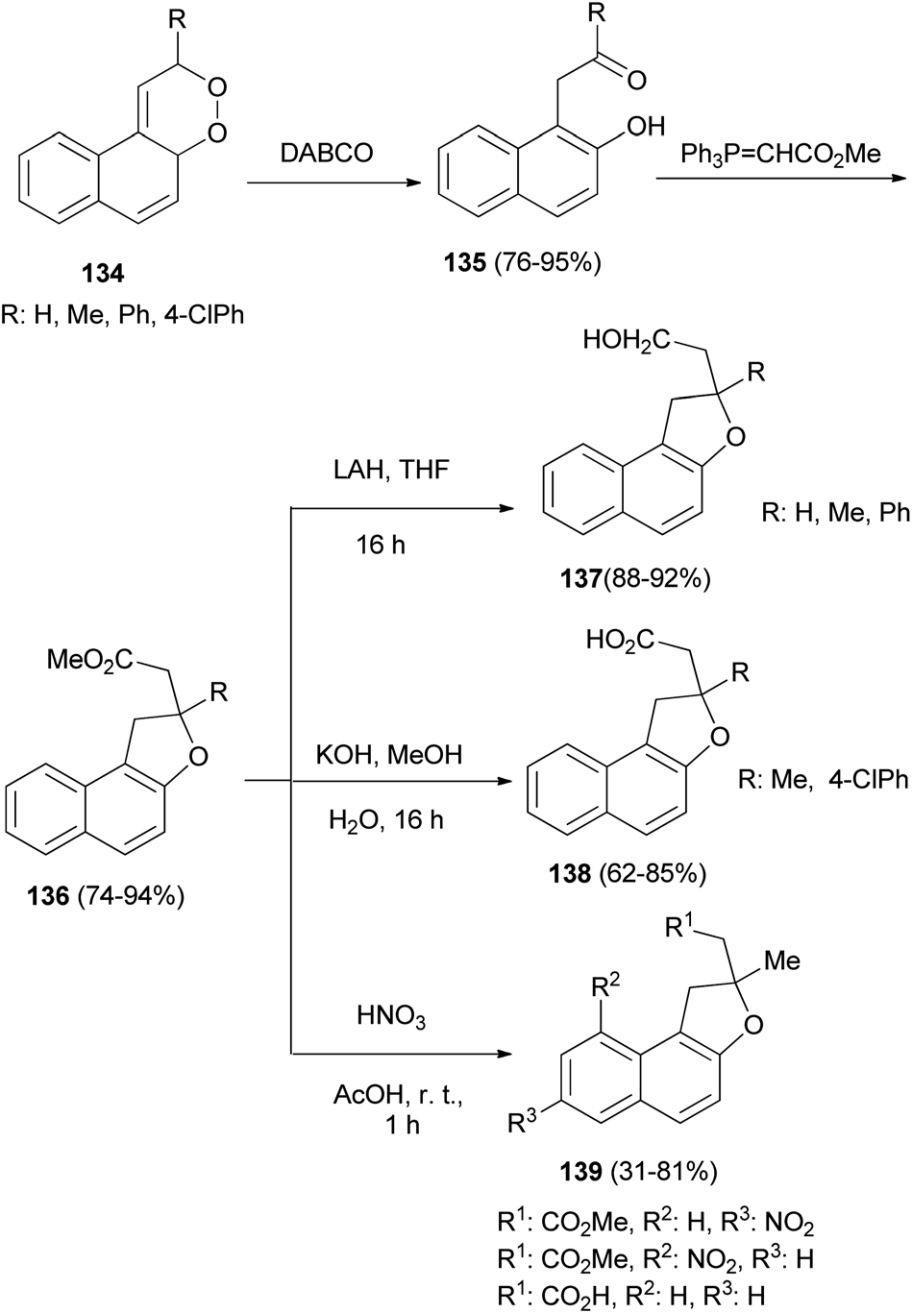
Synthesis of dihydronaphthofuran derivatives 136–139.

Rose bengal bis(triethylammonium) salt sensitized photooxidation of 1-vinylnapthalenes gave the 1,2-dioxines 134. 1,2-Dioxines 134 underwent rearrangement when allowed to react with DABCO to afford the 1-(β-keto)-2-naphthols 135 in excellent yield. Reaction of the naphthols 135 with methyl(triphenylphosphoranylidene) acetate afforded the requisite dihydronaphthofurans 136 in 74–94% yields *via* a Wittig/oxy-Michael sequence. Saponification of the esters 136 afforded the acids 137 in 62–85% yields and LiAlH_4_ reduction of 136 gave the alcohols 138 in 88–92% yields. Reaction of dihydronaphthofurans 136 with nitric acid in glacial acetic acid at room temperature for 1 h afforded 1,2-dihydronaphtho[2,1-*b*]furan derivatives 139 in 31–81% yields (Scheme 65).^61^

**Scheme 66:**
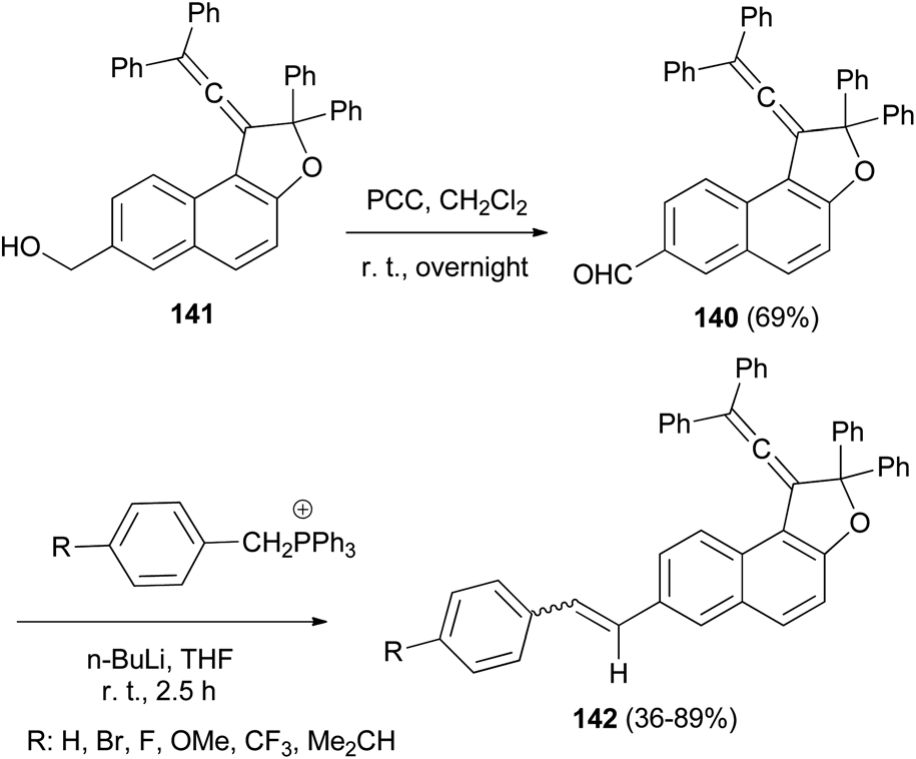
Synthesis of photochromic 1-vinylidenenaphthofurans 142.

1-(2,2-Diphenylvinylidene)-2,2-diphenyl-1,2-dihydronaphtho[2,1-*b*]furan-7-carbaldehyde (140) in 69% yield could be prepared by the reaction of dihydronaphthofuran 141 in the presence of pyridinium chlorochromate in CH_2_Cl_2_ at room temperature for overnight. 1,2-Dihydronaphtho[2,1-*b*]furans 142 were prepared in 36–89% yields by the Wittig reaction of 140 with triarylphosphonium salt in the presence of *n*-BuLi in THF at room temperature for 2.5 h (Scheme 66).^17^

**Scheme 67:**
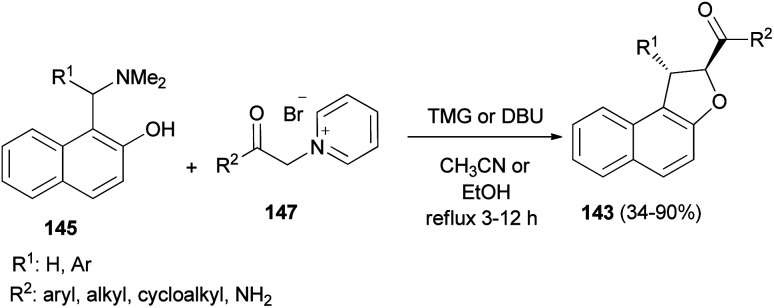
Diastereoselective synthesis of *trans*-1,2-dihydronaphtho[2,1-*b*]furans 143.

**Scheme 68:**
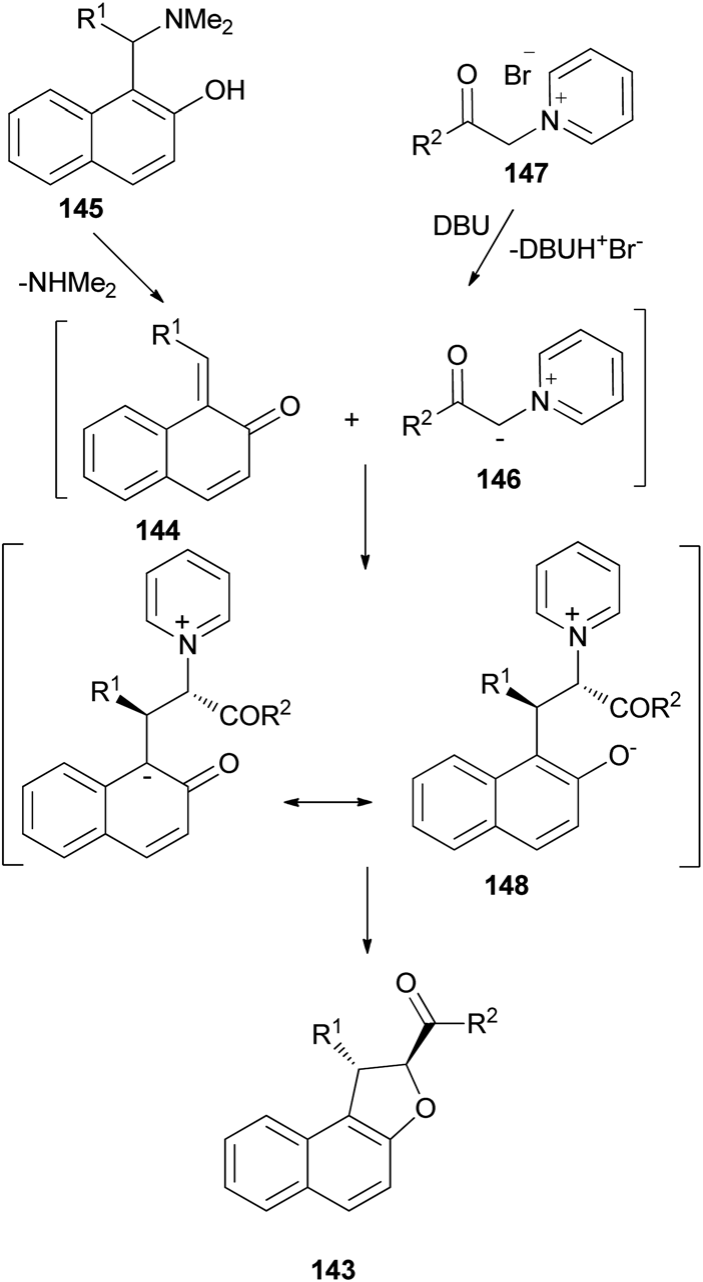
Proposed mechanism for the formation of 143.

Diastereoselective synthesis of 1,2-dihydronaphtho[2,1-*b*]furans 143 in 34–90% yields has been developed by the reactions of *o*-quinone methides 144 from 2-naphthol Mannich base 145 with pyridinium methylides 146 in the presence of 1,1,3,3-tetramethylguanidine (TMG) or DBU in refluxing CH_3_CN or EtOH under an argon atmosphere for 3–12 h (Scheme 67).^62^ A mechanistic rationale portraying the probable sequence of events is given in Scheme 68. The first step is the formation of the two reaction intermediates. *o*-QM 144 is formed by the thermal decomposition of the Mannich base and deprotonation of the pyridinium salt 147 gives ylide 146. The second step is a Michael-type addition of a pyridinium ylide 146 to the electron-deficient *o*-QM to afford the zwitterion intermediate 148 which react further to give products 143.

**Scheme 69:**
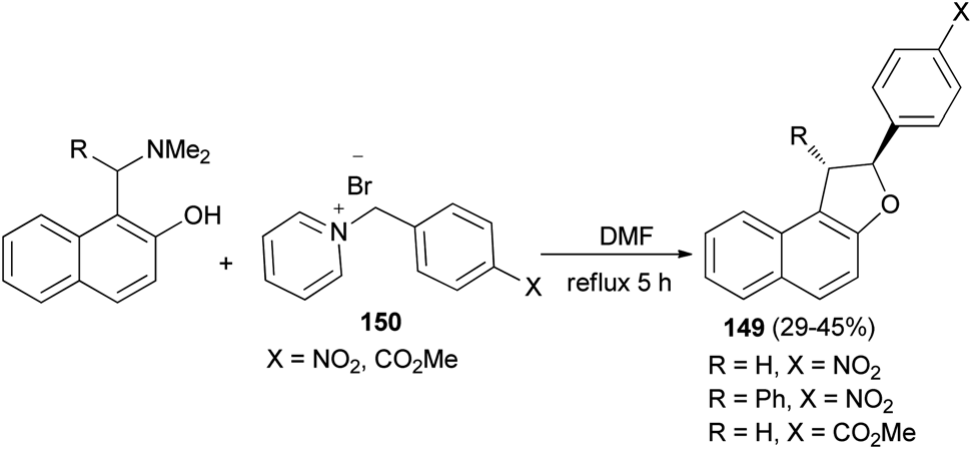
Preparation of 2-aryl-1,2-dihydronaphtho[2,1-*b*]furans 149.

In a similar manner, 2-aryl-1,2-dihydronaphtho[2,1-*b*]furans 149 in 29–45% yields were synthesized by the reaction of 2-naphthol Mannich base and pyridinium salts 150 in refluxing DMF for 5 h under an argon atmosphere (Scheme 69).^62^

**Scheme 70:**
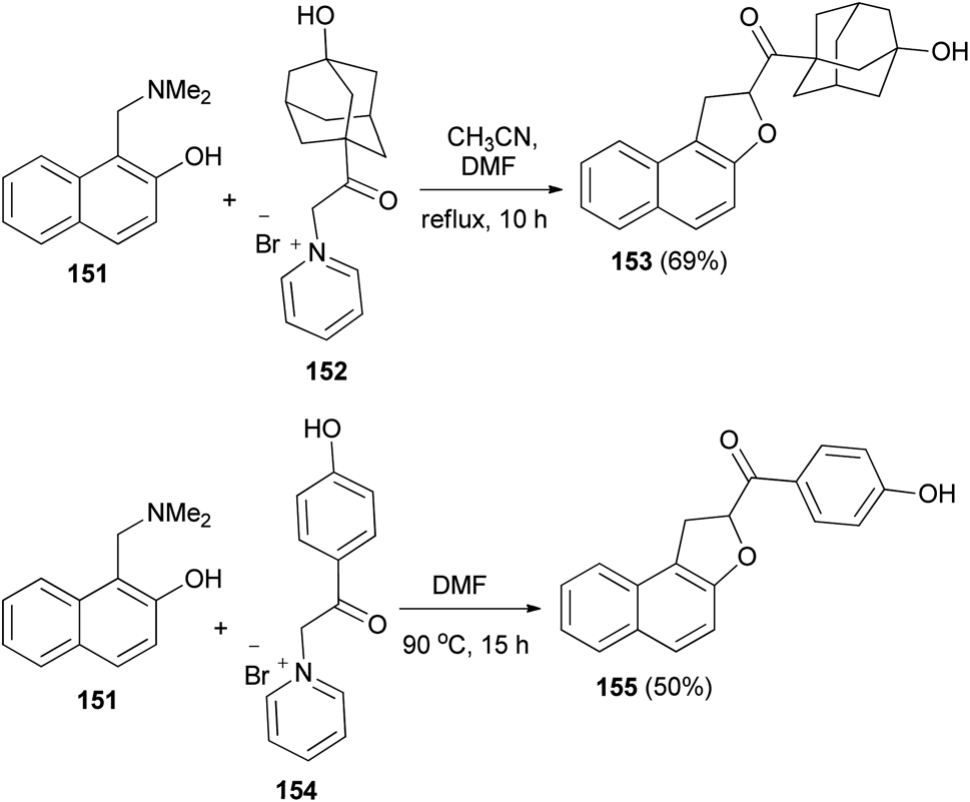
Synthesis of 1,2-dihydronaphtho[2,1-*b*]furans 153 and 155.

Spasov *et al.*^12^ have shown the reaction of 1-((dimethylamino)methyl)naphthalen-2-ol (151) and 1-(2-(3-hydroxyadamantan-1-yl)-2-oxoethyl)pyridin-1-ium bromide (152) in refluxing a mixture of acetonitrile-DMF (3 : 1) for 10 h afforded 1,2-dihydronaphtho[2,1-*b*]furan 153 in 69% yield. In a similar manner, the reaction of 151 with 1-(2-(4-hydroxyphenyl)-2-oxoethyl)pyridin-1-ium bromide (154) in DMF at 90 °C for 15 h gave 1,2-dihydronaphtho[2,1-*b*]furan 155 in 50% yield (Scheme 70).

**Scheme 71:**
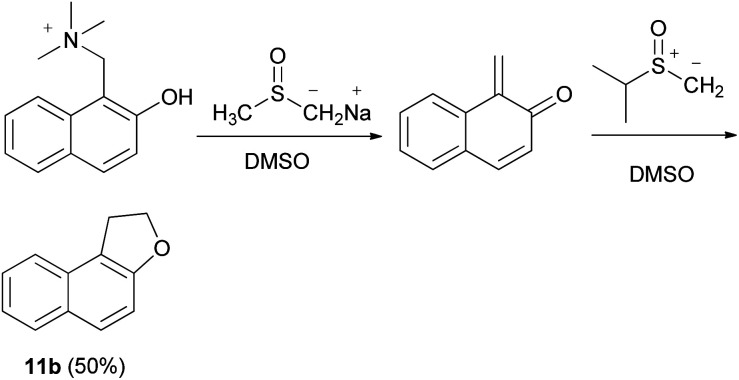
Mannich base synthesis of dihydronaphthofuran 11b.

1,2-Dihydronaphtho[2,l-*b*]furan (11b) could also be obtained in 50% yield from the Mannich base methiodide of 2-naphthol *via* the corresponding quinone methide in the presence of dimethyl sulfoxonium methylide in dimethyl sulfoxide (DMSO) and base (B = Na^+^-CH_2_SOCH_3_) (Scheme 71).^63^

**Scheme 72:**
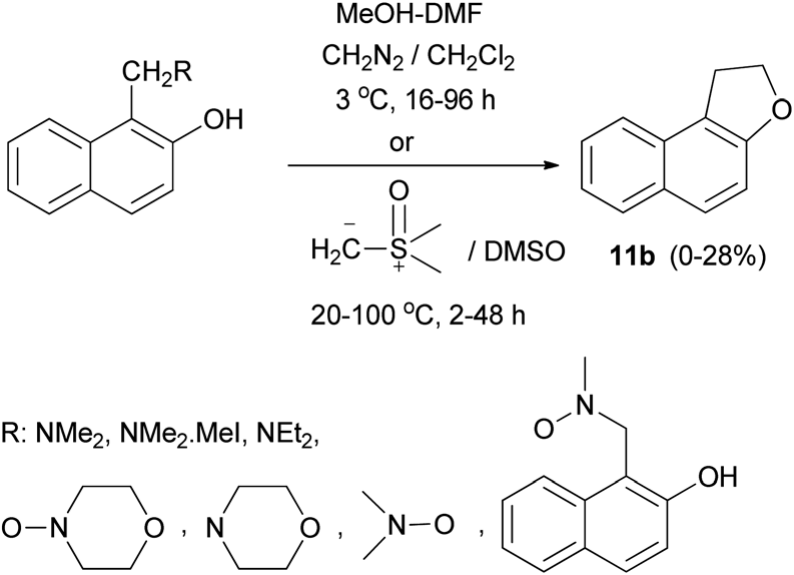
Synthesis of dihydronaphthofuran 11b from phenolic Mannich bases.

In a similar manner, dihydronaphthofuran 11b was obtained by the reaction of Mannich bases, Mannich base methiodides, and Mannich base *N*-oxides derived from naphthols with diazomethane in CH_2_Cl_2_ at 3 °C for 16–96 h or with dimethylsulphoxonium methylide in DMSO at 20–100 °C for 2–48 h in 0–28% yields (Scheme 72).^64^

**Scheme 73:**
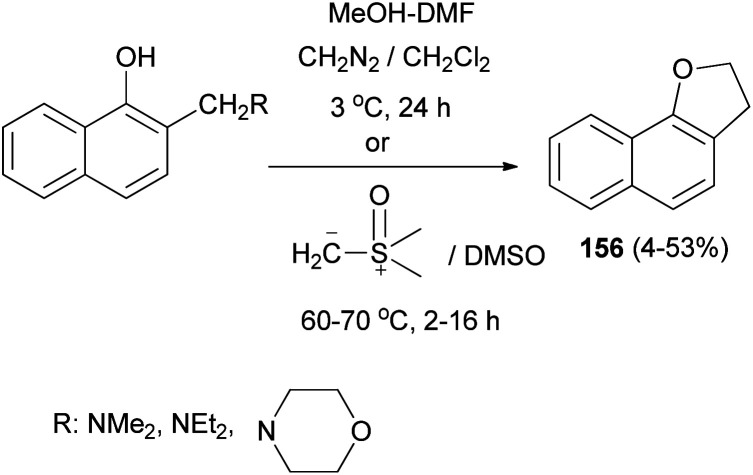
Synthesis of 2,3-dihydronaphtho[1,2-*b*] furan (156).

Also, 2,3-dihydronaphtho[1,2-*b*]furan (156) was obtained in 4–53% yields *via* the reaction of Mannich bases, Mannich base methiodides, and Mannich base *N*-oxides derived from 1-naphthols with diazomethane in CH_2_Cl_2_ at 3 °C for 24 h or with dimethylsulphoxonium methylide in DMSO at 60–70 °C for 2–16 h (Scheme 73).^64^

**Scheme 74:**
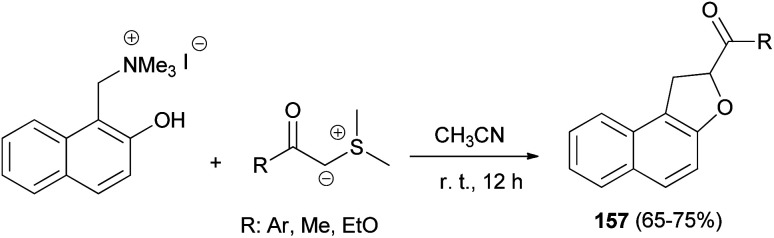
Synthesis of 2-acyl- or 2-aroyl-substituted 1,2-dihydronaphtho[2,1-*b*]furans 157.

A simple and general route to the synthesis of 1,2-dihydronaphtho[2,1-*b*]furans 157, substituted in position 2 by an acyl or aroyl group, starting from phenolic Mannich base methiodides and the carbonyl-stablished sulphonium ylide in CH_3_CN has been developed. The reaction proceeds readily at room temperature for 12 h and usually affords desired products 157 in 65–75% yields (Scheme 74). The formation of the products can be rationalised by assuming the known behaviour of stabilised sulphonium ylides towards system bearing an electrophilic centre and a nucleophilic heteroatom.^65^

**Scheme 75:**
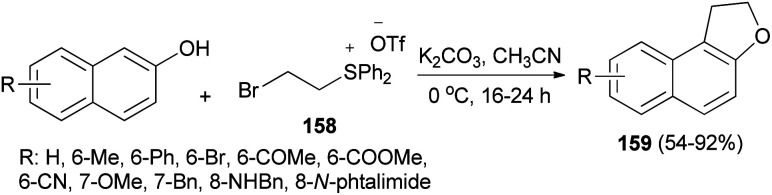
Preparation of dihydronaphthofurans 159.

**Scheme 76:**
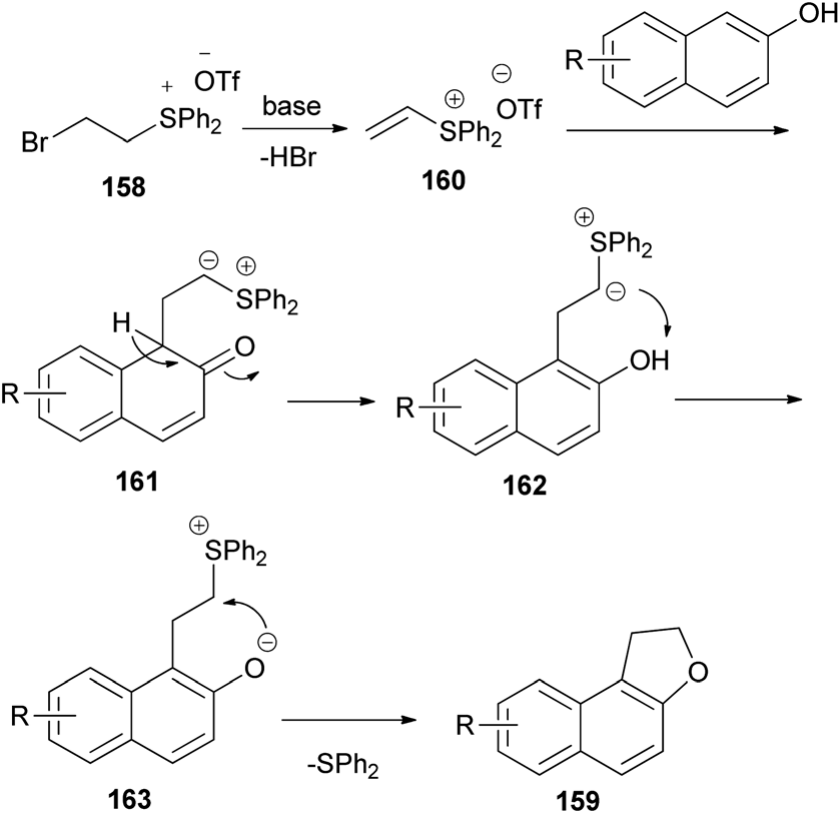
Proposed reaction mechanism for the formation of 159.

The work of Yan *et al.*^66^ demonstrated that annulation reaction of β-naphthols with (2-bromoethyl)diphenylsulfonium trifluoromethanesulfonate salt (158) in the presence of K_2_CO_3_ in CH_3_CN at 0 °C under an argon atmosphere for 16–24 h led to the formation of dihydronaphthofurans 159 in moderate to good yields (54–92%) (Scheme 75). A tentative reaction mechanism is outlined in Scheme 76. The reaction begins with the generation of a vinylsulfonium salt 160*via* the elimination of hydrogen bromide from 158. The vinylsulfonium salt 160 reacts with β-naphthols to give sulfonium ylides 161, which then tautomerize to intermediates 162. After a proton transfer, the zwitterions 163 are formed. The subsequent intramolecular SN_2_ reaction led to the formation of products 159 and eliminates diphenyl sulfide.

### 2.6. Enantioselective synthesis

**Scheme 77:**
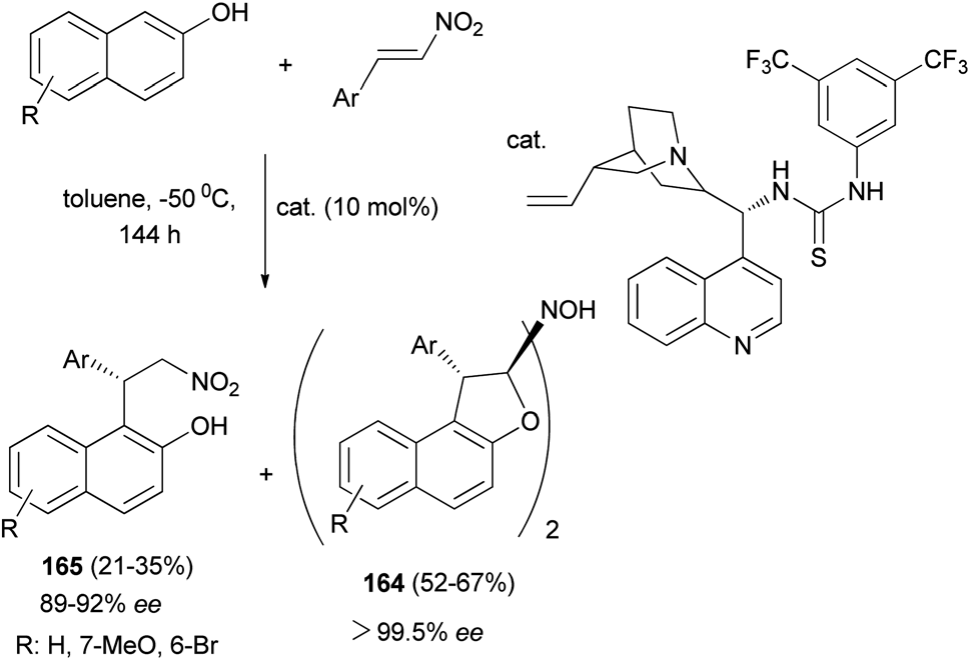
Organocatalyzed synthesis of dimeric tricyclic 1,2-dihydronaphtho[2,1-*b*]furanyl-2-hydroxylamine derivatives 164.

Chen *et al.*^67^ described synthesis of enantiopure 1,2-dihydronaphtho[2,1-*b*]furanyl-2-hydroxylamine derivatives 164 in 52–67% yields with 99.5% ee and products 165 by the asymmetric Michael-type Friedel–Crafts alkylation of 2-naphthols with nitroolefins in toluene in the presence of bifunctional thiourea-tertiary amine organocatalyst at −50 °C for 144 h (Scheme 77).

**Scheme 78:**
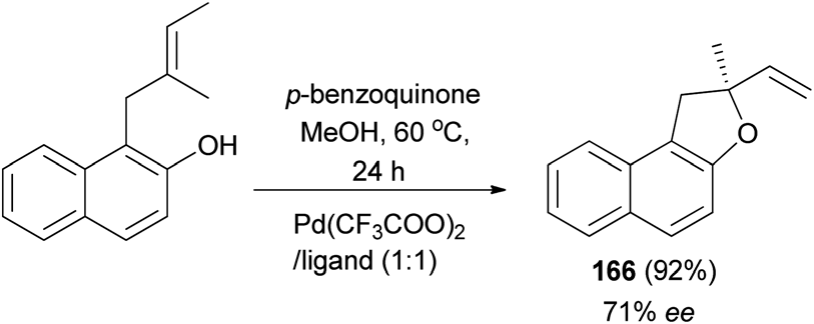
Pd-catalyzed synthesis of dihydronaphtofuran 166.

The work of Zhang *et al.* demonstrated that 2-methyl-2-vinyl-1,2-dihydronaphtho[2,1-*b*]furan (166) was synthesized in 92% yield with enantioselectivity 71% ee by intramolecular Wacker-type cyclizations of *o*-substituted allyl naphthol in the presence of Pd(CF_3_COO)_2_/tetraoxazoline as ligand (Pd/ligand 1 : 1) and *p*-benzoquinone in methanol at 60 °C for 24 h (Scheme 78).^68^

**Scheme 79:**
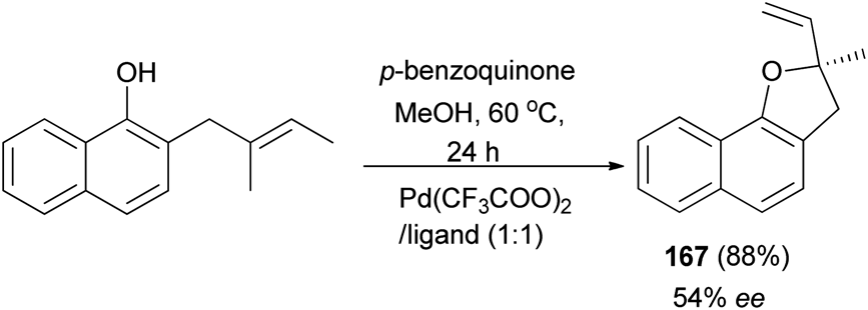
Enantioselective synthesis of 2-methyl-2-vinyl-2,3-dihydronaphtho[1,2-*b*]furan (167).

In a similar fashion, 2-methyl-2-vinyl-2,3-dihydronaphtho[1,2-*b*]furan (167) was synthesized in 88% yield and enantioselectivity 54% ee (Scheme 79).^68^

**Scheme 80:**
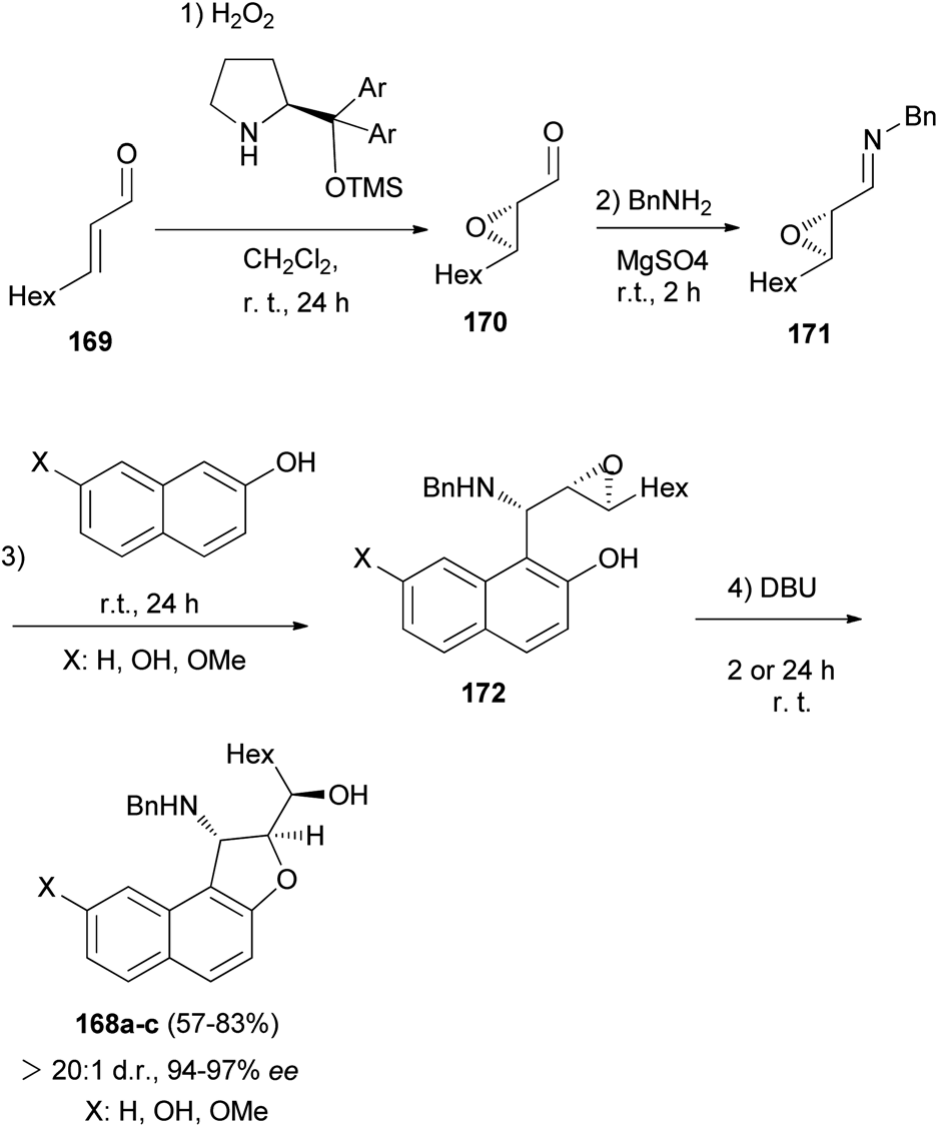
Enantioselective synthesis of dihydronaphthofurans 168a–c.

Asymmetric organocatalytic synthesis of optically active dihydronaphthofurans 168a–c has been reported. Treatment of *trans*-2-nonenal 169 with H_2_O_2_ in CH_2_Cl_2_ at room temperature for 24 h afforded 2,3-epoxy aldehyde 170. Then, the reaction of 2,3-epoxy aldehyde 170 with BnNH_2_ at room temperature in the presence of MgSO_4_ gave epoxy imine 171 as 1,2-di-electrophilic species. Friedel–Crafts alkylation of 2-naphthols with epoxy imine at room temperature afforded intermediate 172. Subsequent 5-*exo*-tet epoxide opening of 172 through the hydroxyarenic oxygen atom in the presence of DBU at room temperature led to the formation of dihydronaphthofurans 168 in 57–83% yields and excellent enantioselectivities (94–97% ee) (Scheme 80).^69^

**Scheme 81:**
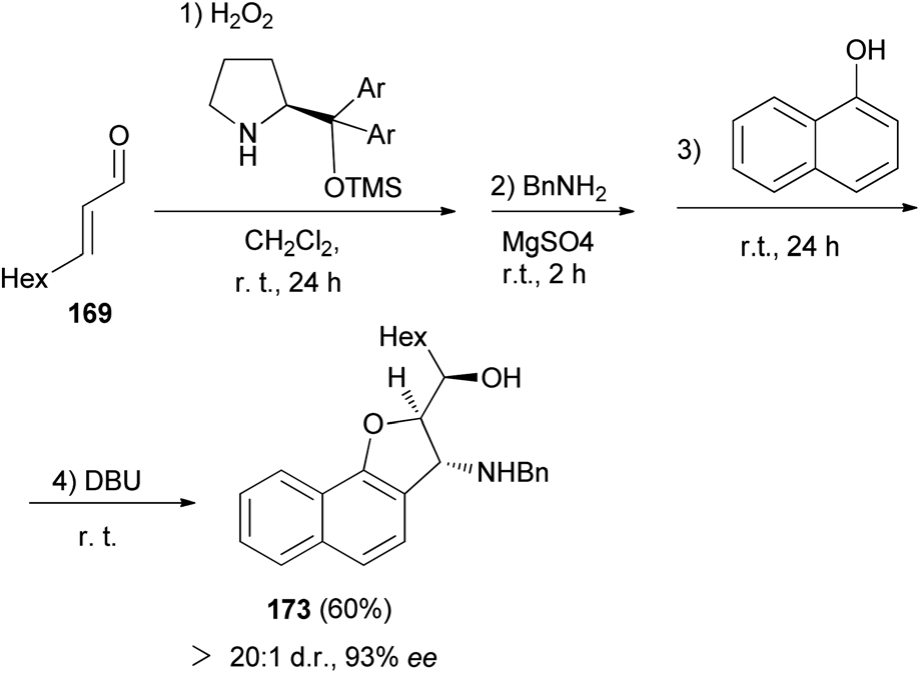
Enantioselective synthesis of 2,3-dihydronaphtho[1,2-*b*]furan 173.

Moreover, 2,3-dihydronaphtho[1,2-*b*]furan 173 in 60% yield and excellent enantioselectivitie (93% ee) was obtained *via* the same reaction conditions that it has been reported for the synthesis of 168 (Scheme 81).^69^

**Scheme 82:**
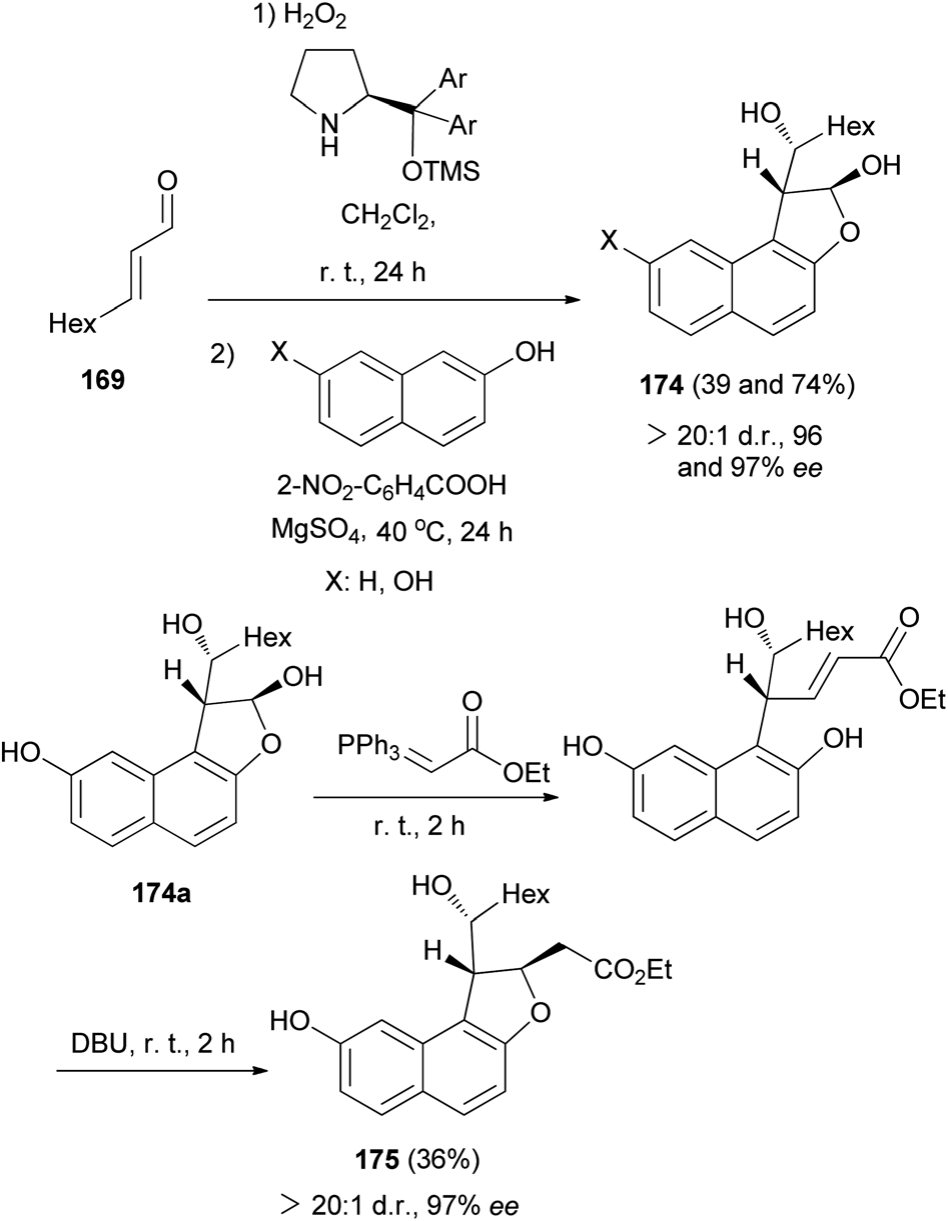
Enantioselective synthesis of 1,2-dihydronaphtho[2,1-*b*]furans 174 and 175.

In a similar fashion, reaction of 2,3-epoxy aldehyde 170 with naphthols in the absence of BnNH_2_ and DBU gave dihydronaphthofurans 174 in 39 and 74% yields and excellent enantioselectivities (96 and 97% ee). Also, dihydronaphthofuran 174a (X: OH) *via* the Wittig reaction, using the stabilized phosphorus ylide, and subsequent base-induced oxa-Michael addition proved successful, thus affording access to the dihydrobenzofuran 175 in 36% yield and 97% ee (Scheme 82).^69^

**Scheme 83:**
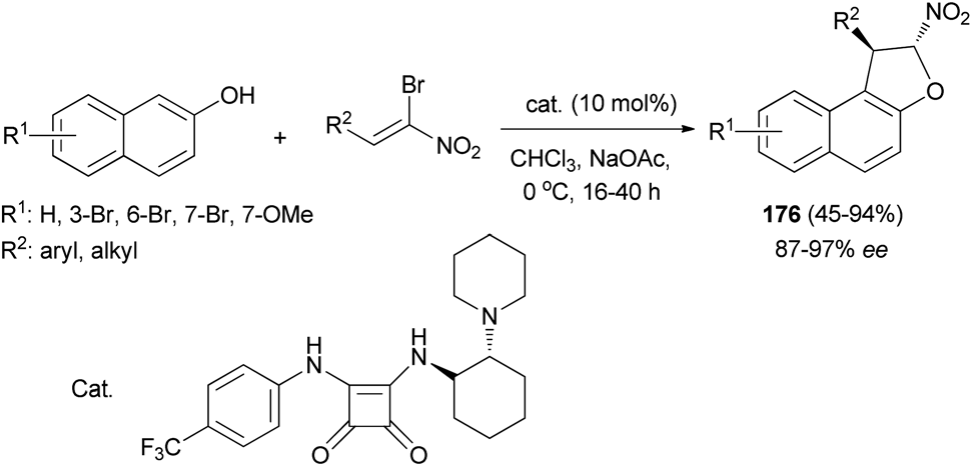
Squaramide catalyzed synthesis of *trans*-dihydroarylfurans 176.

Asymmetric synthesis of *trans*-dihydroarylfurans 176 in 45–94% yields and excellent ee's (up to 97%) *via* a Friedel–Crafts/substitution domino reaction of (*Z*)-bromonitroalkenes and 2-naphthols in the presence of squaramide as catalyst and co-base (sodium acetate) in CHCl_3_ at 0 °C for 16–40 h has been reported (Scheme 83).^70^

**Scheme 84:**
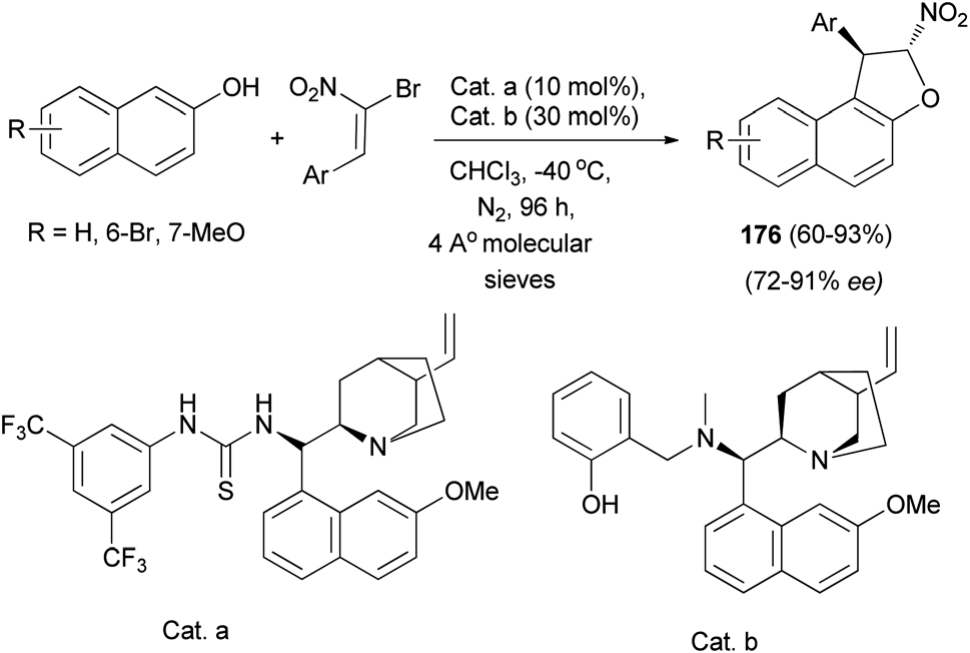
Chiral multiple catalysis synthesis of 1,2-dihydronaphtho[2,1-*b*] furans 176.

Also, a chiral multiple catalysis with two chiral units has been proved effective in the domino Friedel–Crafts alkylation of 2-naphthols with α-bromonitroalkenes. This efficient domino reaction affords chiral 1,2-dihydronaphtho[2,1-*b*] furans 176 in good to high yields (up to 93% yield) and high enantioselectivities (91% enantiomeric excess) (Scheme 84).^71^

**Scheme 85:**
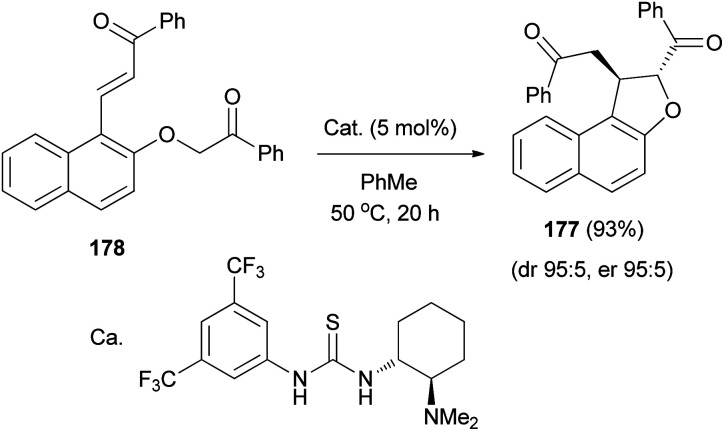
Tertiary amine-thiourea catalyzed synthesis of dihydronaphthofuran 177.

The diastereo- and enantioselective synthesis of 2,3-disubstituted *trans*-2,3-dihydronaphthofuran 177 (95 : 5 dr, 95 : 5 er) *via* intramolecular Michael addition has been developed using keto-enone substrate 178 and a bifunctional tertiary amine-thiourea catalyst (5 mol%) in toluene at 50 °C for 20 h in 93% yield (Scheme 85).^72^

**Scheme 86:**
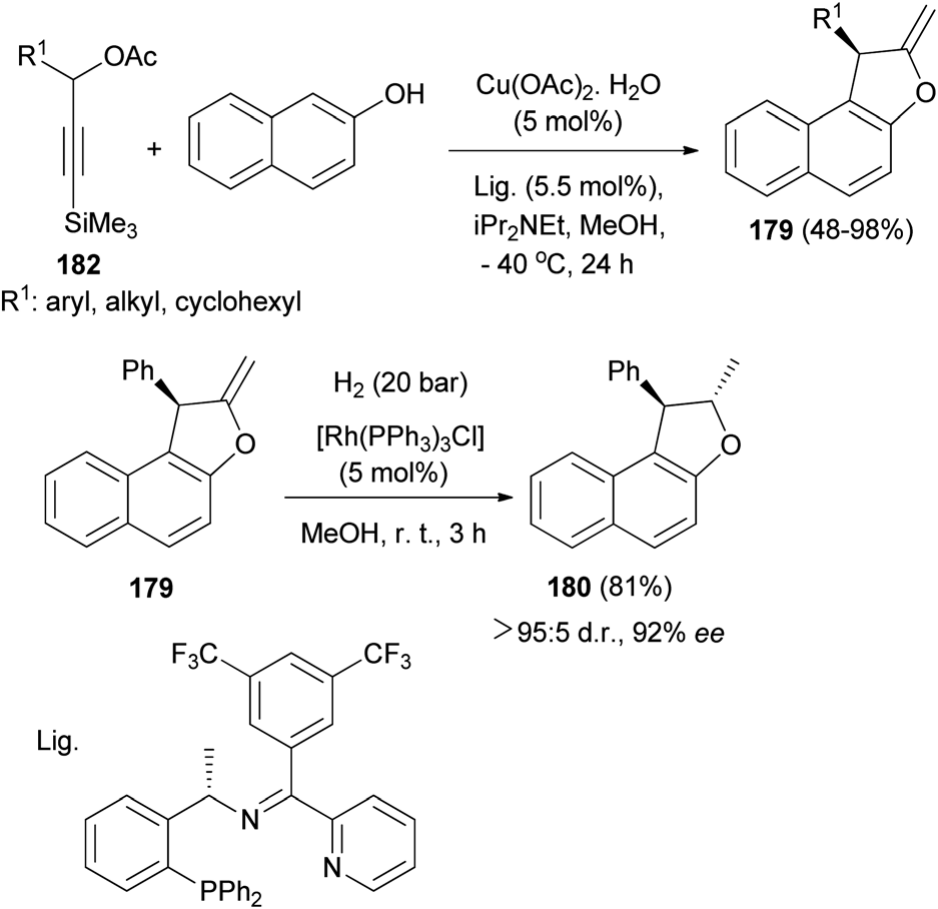
Cu-(OAc)_2_·H_2_O-catalyzed enantioselective synthesis of 1,2-dihydronaphtho[2,1-*b*]furans 179 and 180.

A copper-catalyzed asymmetric [3 + 2] cycloaddition of 3-trimethylsilylpropargylic esters with 2-naphthols has been realized and proceeds by a desilylation-activated process. Under the catalysis of Cu-(OAc)_2_·H_2_O in combination with a structurally optimized ketimine P,N,N-ligand and iPr_2_NEt in MeOH at −40 °C for 24 h a wide range of optically active 1,2-dihydronaphtho[2,1-*b*]furans 179 were obtained in good yields (48–98%) and with high enantioselectivities (up to 93% ee). Hydrogenation of (*R*)-2-methylene-1-phenyl-1,2-dihydronaphtho[2,1-*b*]furan 179 in the presence of [Rh(PPh_3_)_3_Cl] (5 mol%) in MeOH at room temperature for 3 h afforded (1*R*, 2*S*)-2-methyl-1-phenyl-1,2-dihydronaphtho[2,1-*b*]furan 180 in 81% yield and excellent diastereoselectivity without the obvious erosion in enantioselectivity (Scheme 86).^73^

**Scheme 87:**
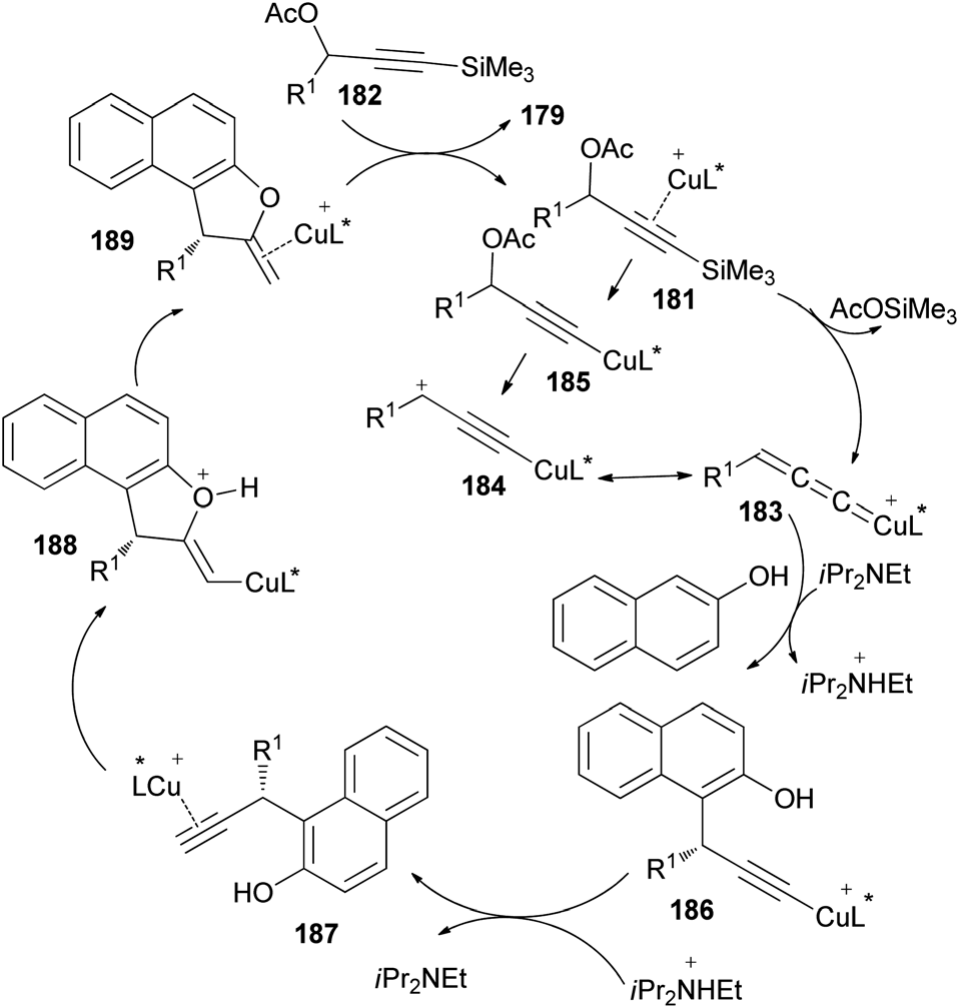
Proposed mechanism for the synthesis of 179.

A reaction pathway for the preparation of 179 is proposed as shown in Scheme 87. In the first step, a copper complex forms the π-complex 181 with 182. A copper-promoted Si–C(sp) bond cleavage of 181, followed by the elimination of an acetoxyl moiety, affords the copper allenylidene complex 183 or its resonance structure 184. The intramolecular Cu–Si exchange by the elimination of AcOSiMe_3_ proceeds more readily than the intermolecular base-assisted reaction with terminal propargylic esters for the formation of copper acetylide complex 185, which should be answered for the observed activation. The nucleophilic attack of the C_α_ atom of β-naphthol at the C_γ_ atom of the allenylidene complex 183 gives the copper acetylide complex 186, which is then converted into the copper π–alkyne complex 187. Intramolecular nucleophilic attack of the hydroxy group on the C_β_ atom of 187 generates an alkenyl complex 188, which is transformed into the copper π–alkene complex 189. The starting 181 is then regenerated from 189 by liberating 179 through ligand exchange with another 182.^73^

**Scheme 88:**
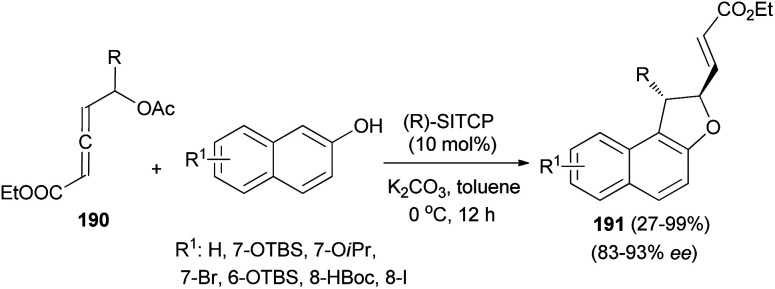
Phosphine-catalyzed asymmetry synthesis of 1,2-dihydronaphtho[2,1-*b*]furans 191.

**Scheme 89:**
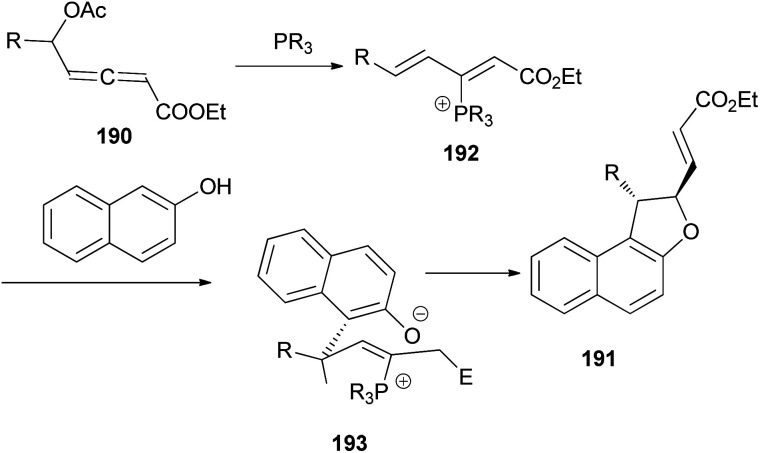
A plausible mechanism for the preparation of 191.

Wang and Tong demonstrated that phosphine-catalyzed asymmetric [3 + 2] annulations of δ-acetoxy allenoates 190 with 2-naphthols in the presence of K_2_CO_3_ in toluene at 0 °C for 12 h afforded 1,2-dihydronaphtho[2,1-*b*]furans 191 in moderate to excellent yields (27–99%) and with high enantioselectivity (83–93%) (Scheme 88). In the presence of phosphine catalyst, allenoate 190 can be readily converted into 3-phosphonium-2,4-dienoate 192*via* 1,4-addition of phosphine and subsequent 1,2-elimination of acetate group. For 192, the αC-position has more steric hindrance than the δC-position. Therefore, to minimize the steric repulsion, 2-naphthol would preferentially attack intermediate 192 at its δC-position *via* a Friedel–Crafts type process to afford intermediate 193, which underwent oxa-Michael addition *via* a half-chair conformation, thus finally leading to product 191 (Scheme 89).^74^

**Scheme 90:**
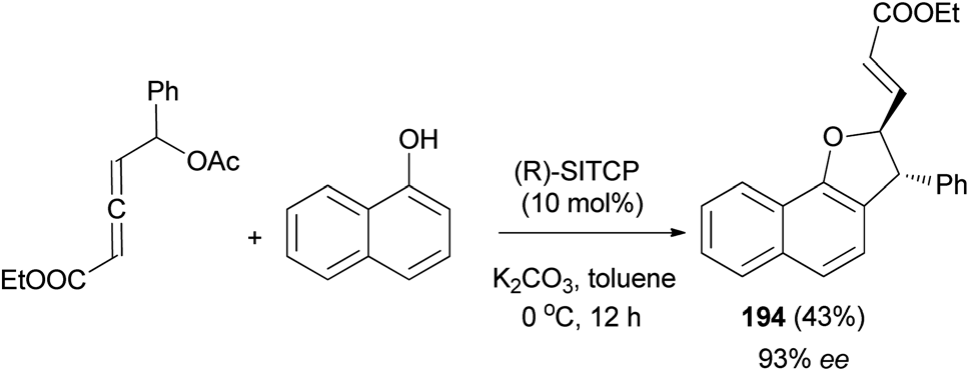
Enantioselective synthesis of 2,3-dihydronaphtho[1,2-*b*]furan derivative 194.

Similarly, phosphine-catalyzed asymmetric [3 + 2] annulation of δ-acetoxy allenoate with 1-naphthol afforded 2,3-dihydronaphtho[1,2-*b*]furan derivative 194 in 43% yield with high enantioselectivity (93% ee) (Scheme 90).^74^

**Scheme 91:**
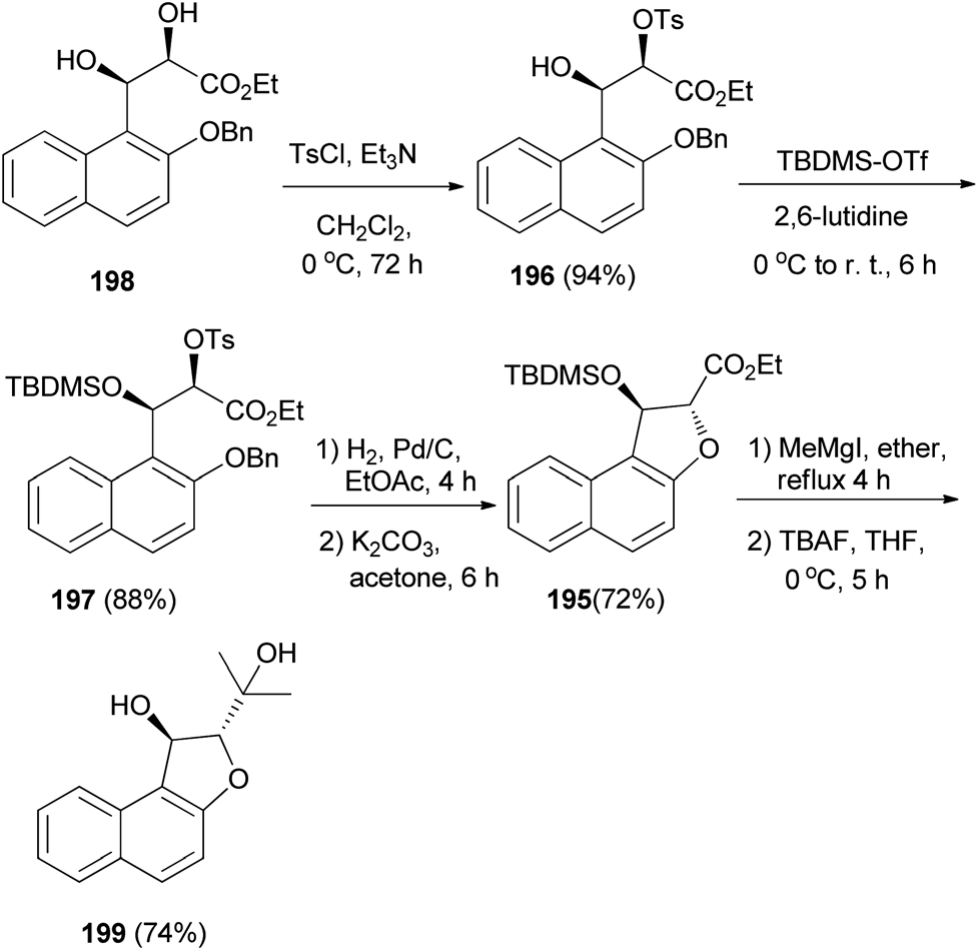
Enantioselective synthesis of dihydronaphthofurans 195 and 199.

The enantioselective synthesis of naphtho-annulated oxa-heterocycle dihydronaphthofuran 195 in 72% yield was accomplished by using β-hydroxy-α-tosyloxy esters 196 and 197 as chiral building blocks, which are easily accessible through the regioselective α-tosylation of Sharpless asymmetric dihydroxylation-derived *syn*-2,3-dihydroxy ester 198. Treatment of dihydronaphthofuran 195 with methylmagnesium iodide in refluxing ether for 4 h followed addition of tetra-*n*-butylammonium fluoride (TBAF) at 0 °C and stirring for 5 h afforded (2*S*,3*S*)-2-(1-hydroxy-1-methyl-ethyl)-1,2-dihydronaphtho[2,1-*b*]furan-1-ol (199) in 74% yield (Scheme 91).^75^

### 2.7. Microwave-assisted synthesis

**Scheme 92:**
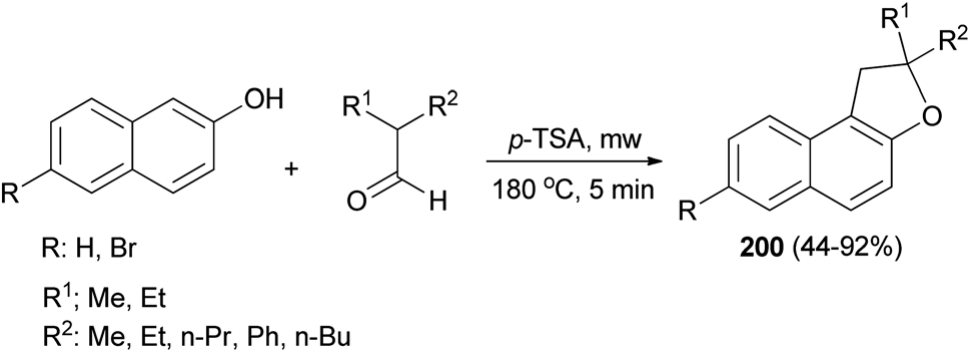
Microwave assisted synthesis of 1,2-dihydronaphtho[2,1-*b*]furans 200.

**Scheme 93:**
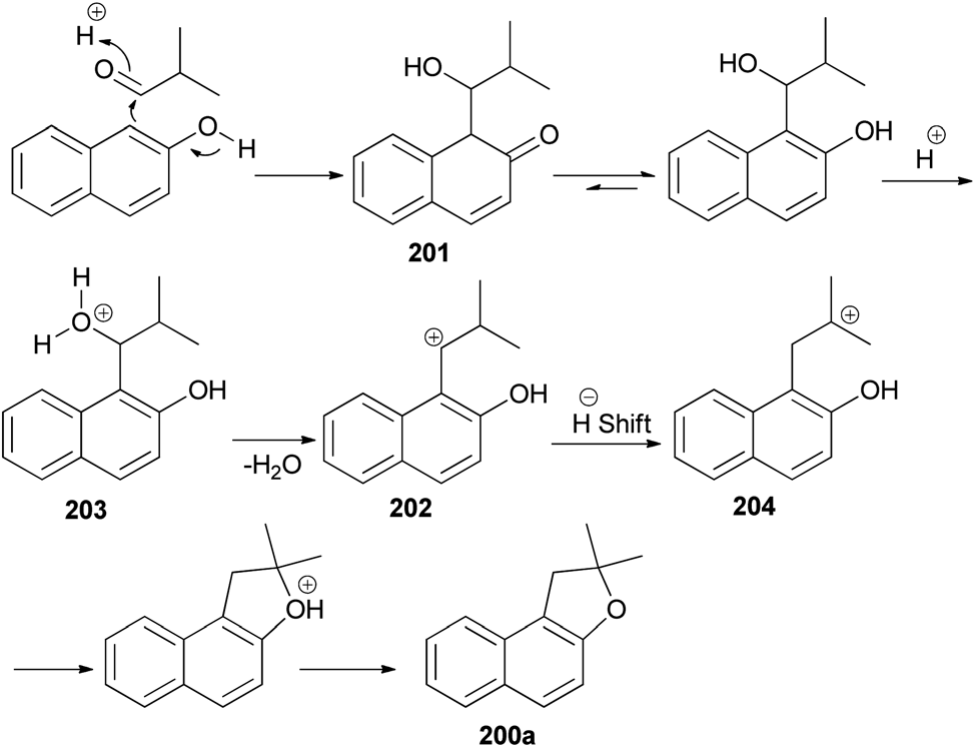
Proposed mechanism of formation of 200a.

The reaction of 2,2-dialkylacetaldehydes with electron-rich 2-naphthols in the presence of *p*-TSA under closed-vessel solvent-free microwave irradiation condition at 180 °C for 5 min resulted in the formation of corresponding 1,2-dihydronaphtho[2,1-*b*]furans 200 in 44–92% yields (Scheme 92). The reaction was carried out *via* oxidative annulation process. In several cases, small amounts of 14-alkyl-14-*H*-dibenzo[*a*,*j*]xanthenes were also formed.^34,76^ The proposed mechanism using isobutyraldehyde and 2-naphthol as starting materials is shown in Scheme 93. Nucleophilic C attack of 2-naphthol led to formation of a secondary alcohol intermediate 201 which formed a secondary benzylic carbocation 202 through corresponding oxonium ion 203 under catalytic amount of *p*-TSA. 1,2-Hydride shift leading a tertiary carbocation 204 followed by nucleophilic attack by the naphthol oxygen led to formation of 200a.^76^

**Scheme 94:**
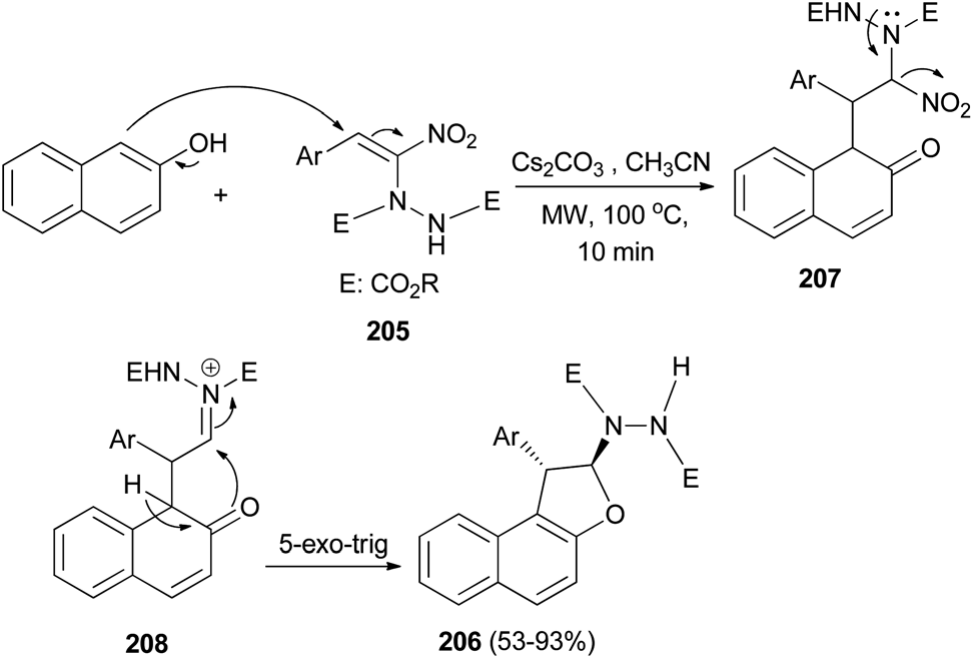
[3 + 2] annulation of 2-naphthol with hydrazinonitroalkene 205 to dihydronaphthofurans 206.

Namboothiri *et al.*^77^ exploited [3 + 2] annulation of β-naphthol with hydrazinonitroalkene 205 in the presence of Cs_2_CO_3_ in CH_3_CN under microwave radiation at 100 °C for 10 min afforded *trans*-1,2-dihydronaphtho[2,1-*b*]furans 206 in 53–93% yields. The proposed mechanism, taking β-naphthol as the representative arenol, envisages Cs_2_CO_3_ mediated addition of naphthol to MBH adduct 205 in a Michael fashion leading to the formation of intermediate 207 which forms an acyliminium type intermediate 208 with the loss of nitro group. Enolization of 208 and intramolecular 5-*exo*-trig cyclization gives rise to product 206 (Scheme 94).

**Scheme 95:**
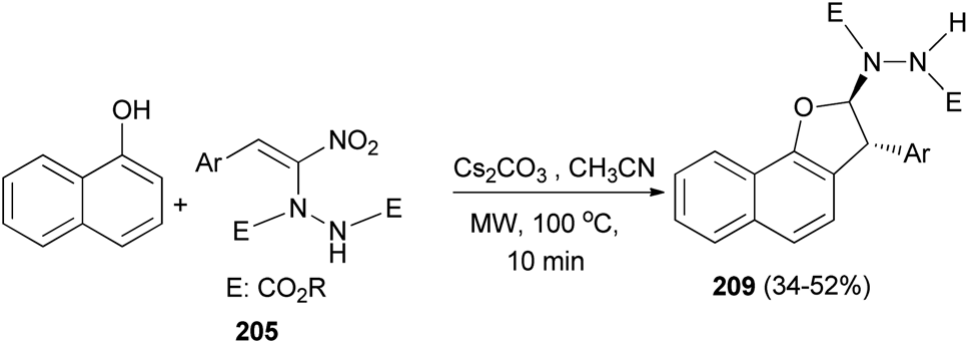
Microwave-assisted synthesis of *trans*-2,3-dihydronaphtho[1,2-*b*]furans 209.

Also, *trans*-2,3-dihydronaphtho[1,2-*b*]furans 209 was obtained in 34–52% yields *via* the reaction of 1-naphthol with hydrazinonitroalkene 205 (Scheme 95).^77^

**Scheme 96:**
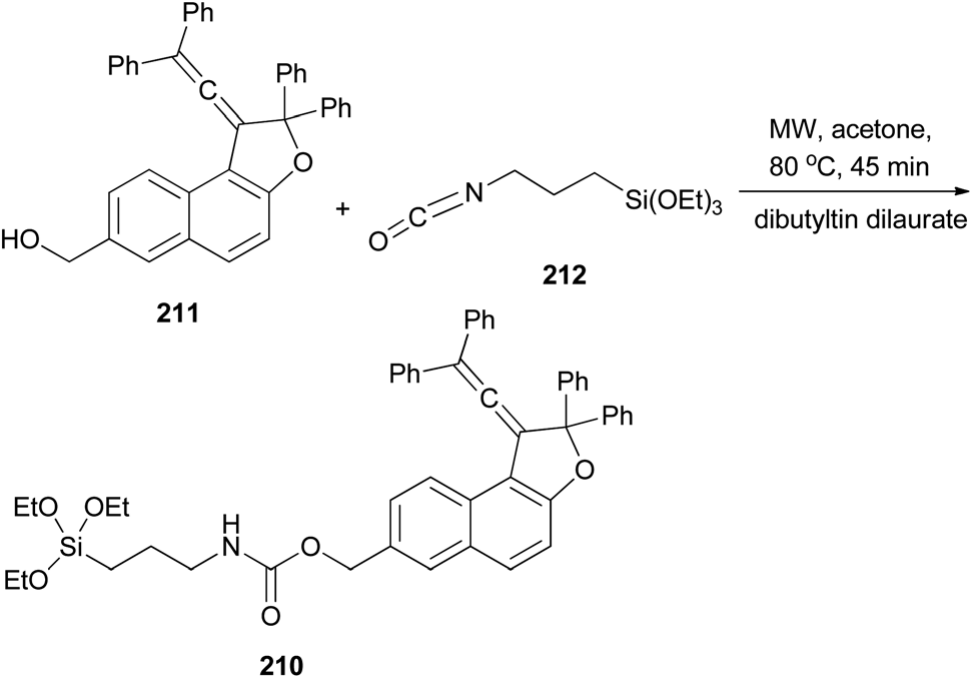
Synthesis of photochromic 1-vinylidene-naphtho[2,1-*b*]furan 210.

Freire *et al.*^18^ have shown that synthesis of novel photochromic 1-(2,2-diphenylvinylidene-2,2-diphenyl-1,2-dihydronaphtho[2,1-*b*]furan-7-yl)methyl (3-(triethoxysilyl)propyl)carbamate (210) was accomplished by the treatment of 211 with IsoTES 212 by a microwave-assisted reaction in the presence of dibutyltin dilaurate as catalyst in acetone at 80 °C during 45 min (Scheme 96).

### 2.8. Photochemical methods

**Scheme 97:**
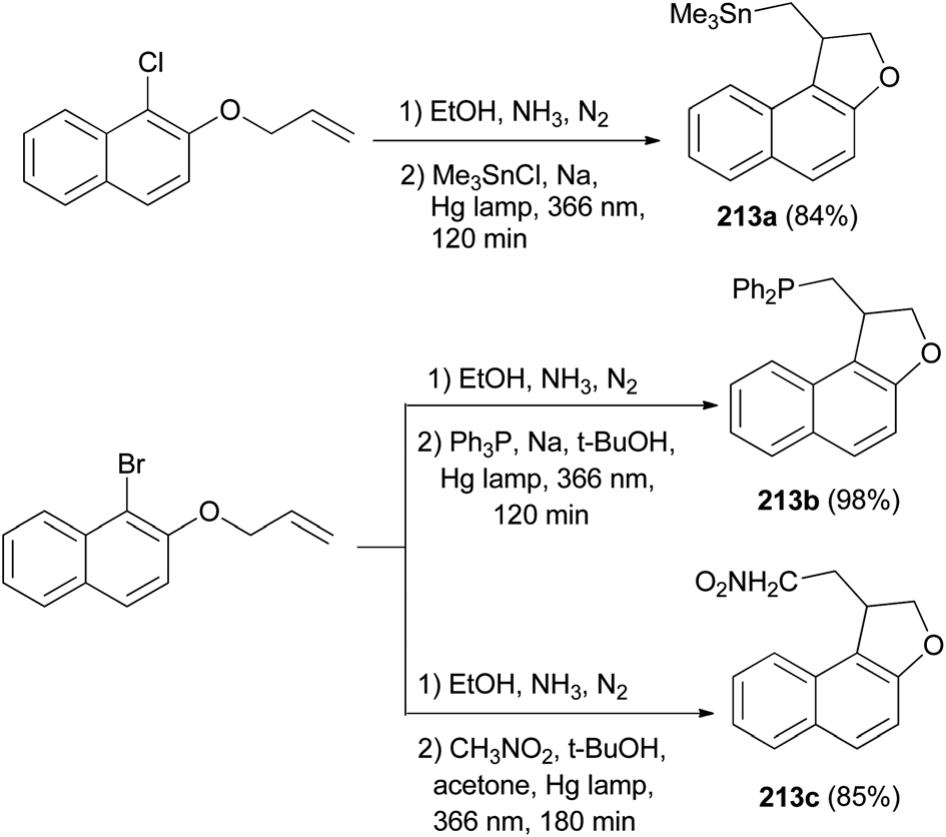
Photostimulated synthesis of 1,2-dihydronaphtho[2,1-*b*]furans 213.

**Scheme 98:**
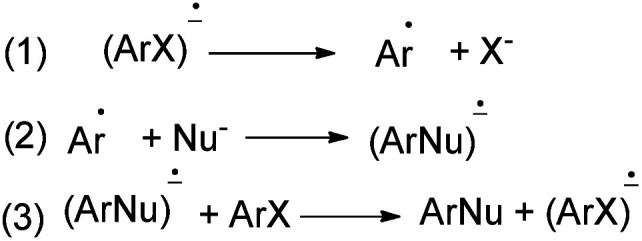
Propagation steps of an S_RN_1 mechanism.

Rossi *et al.*^78^ published synthesis of 1,2-dihydronaphtho[2,1-*b*]furans 213a–c in very good yields by S_RN_1 photostimulated reactions in liquid ammonia from adequate 2-(allyloxy)-1-halonaphthalene and Me_3_Sn^−^, Ph_2_P^−^, and ^−^CH_2_NO_2_ anions. The mixture of 2-(allyloxy)-1-chloronaphthalene in EtOH, liquid ammonia and Me_3_SnCl in dried diethyl ether and Na was irradiated for 120 min using two medium-pressure mercury lamps emitting maximally at 366 nm gave 1,2-dihydronaphtho[2,1-*b*]furan 213a in 84% yield. The reaction of 2-(allyloxy)-1-bromonaphthalene was performed by a procedure similar to that described for the other two nucleophiles, Ph_3_P and Na in *t*-BuOH and CH_3_NO_2_, *t*-BuOK in acetone afforded 1,2-dihydronaphtho[2,1-*b*]furans 213b–c in 98% and 85% yields, respectively (Scheme 97). The propagation steps of an S_RN_1 mechanism are presented in Scheme 98.

**Scheme 99:**
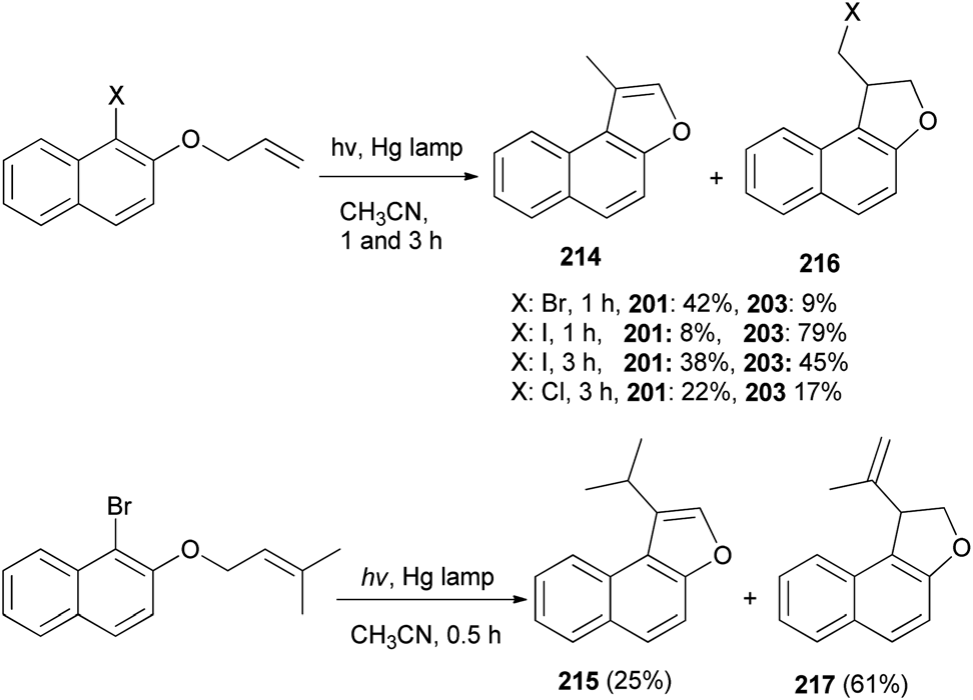
Photochemical synthesis of naphtho[*b*]furans 214–215 and dihydronaphthofurans 216–217.

**Scheme 100:**
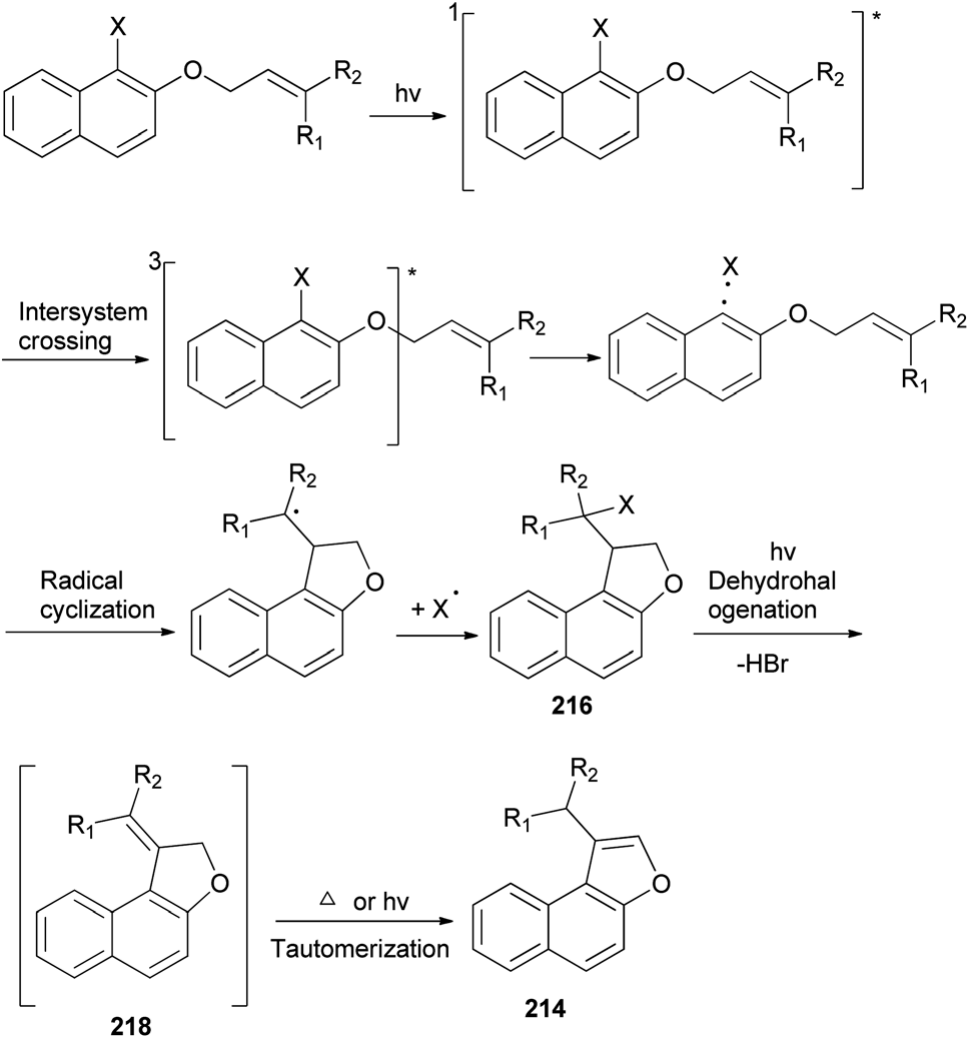
Plausible mechanism for the preparation of 214–217.

A mixture of naphtho[*b*]furans 214–215 and dihydronaphthofuran 216–217 were obtained from allyl halonaphthyl ethers employing photo induced radical cyclization with 100 W high-pressure mercury lamp in CH_3_CN under Ar atmosphere for 0.5–3 h (Scheme 99). In addition, observations made in this effort suggest that a plausible mechanism for these photoreactions (Scheme 100) begins with homolytic C–halogen bond cleavage in the triplet states of the substrates to generate radical pairs, which undergo 5-*exo* type cyclization and halogen atom capture to produce the initially formed 2-halomethyl substituted naphthodihydrofurans 216 similar to atom-transfer radical cyclizations. Subsequently, photoinduced dehydrohalogenation of 216 takes place to generate alkylidene dihydrofuran intermediates 218 that undergo tautomerization to generate the naphthofuran product 214. In the case of R_1_ = CH_3_, R_2_ = CH_3_, the photochemical dehydrohalogenation leads to the respective formation of 218 and the regioisomeric product 217, which is photochemically tautomerized to 215.^79^

### 2.9. Electrochemical methods

**Scheme 101:**
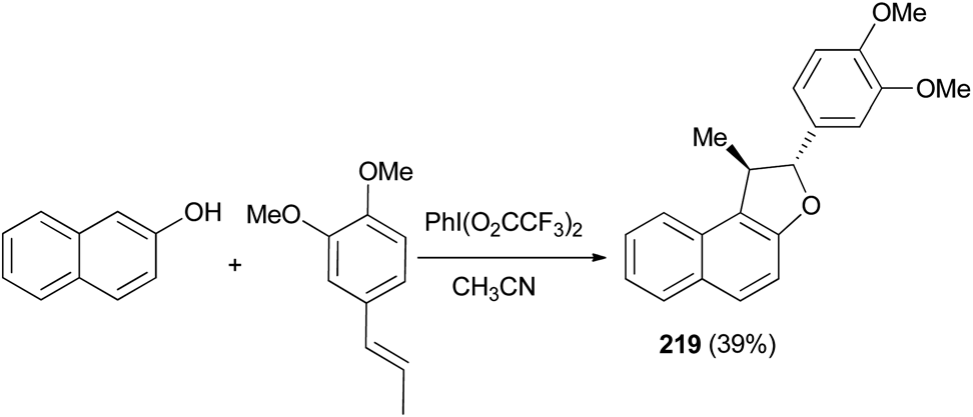
Electrochemical synthesis of dihydronaphthofuran 219.

Treatment of 2-naphthol with 1,2-dimethoxy-4-propenylbenzene in the electrochemical oxidation using iodobenzene bis-trifluoroacetate (PhI(O_2_CCF_3_)_2_) as oxidant in CH_3_CN, gave a very low yield of l,2-dihydronaphtho[2,1-*b*] furan 219 (Scheme 101).^80^

**Scheme 102:**
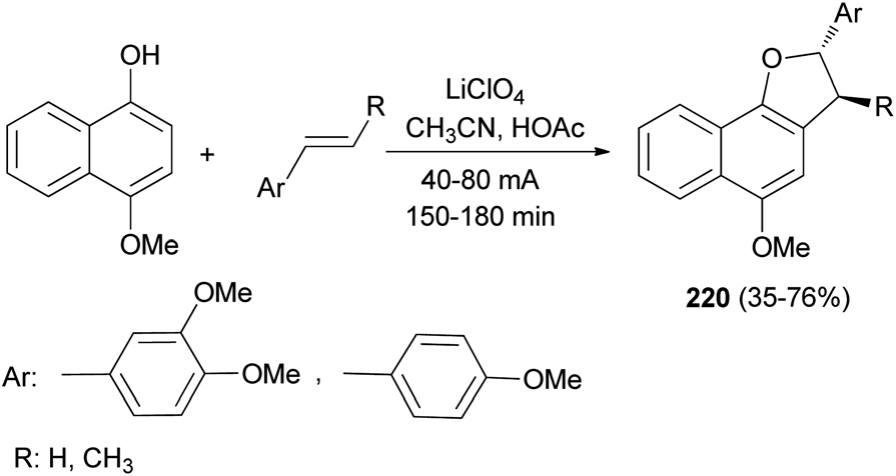
Electrochemical synthesis of 2,3-dihydronaphtho [1,2-*b*]furan derivatives 220.

Anodic oxidation of 4-methoxynaphthol and electron-rich styrenes gave 2,3-dihydronaphtho[1,2-*b*]furan derivatives 220 in 35–76% yields. Reactions were conducted with a current density of 40–80 mA for 150–180 min in 8 : 1 CH_3_CN/HOAc using a platinum anode and cathode and perchlorate as supporting electrolyte (Scheme 102).^80^

**Scheme 103:**
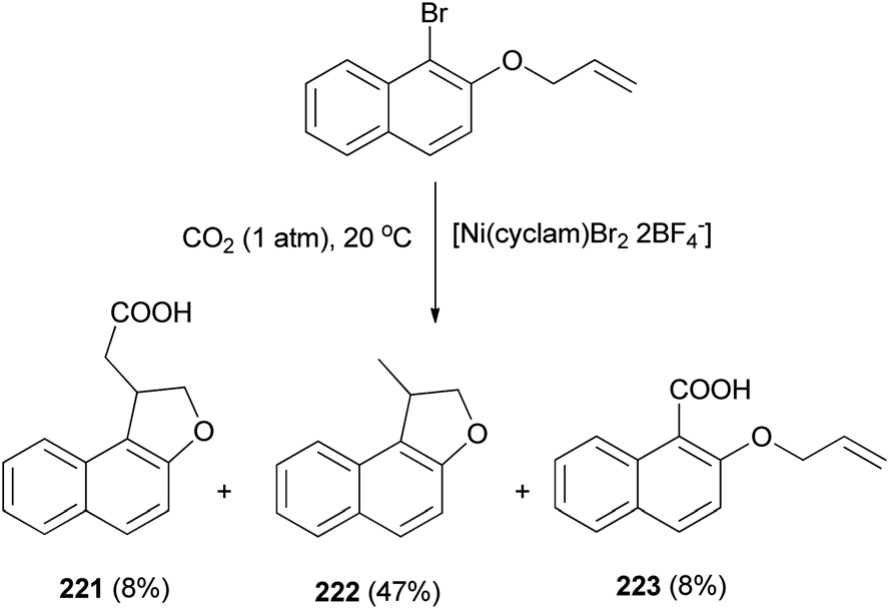
Electrochemical synthesis of dihydronaphthofurans 221 and 222.

**Scheme 104:**
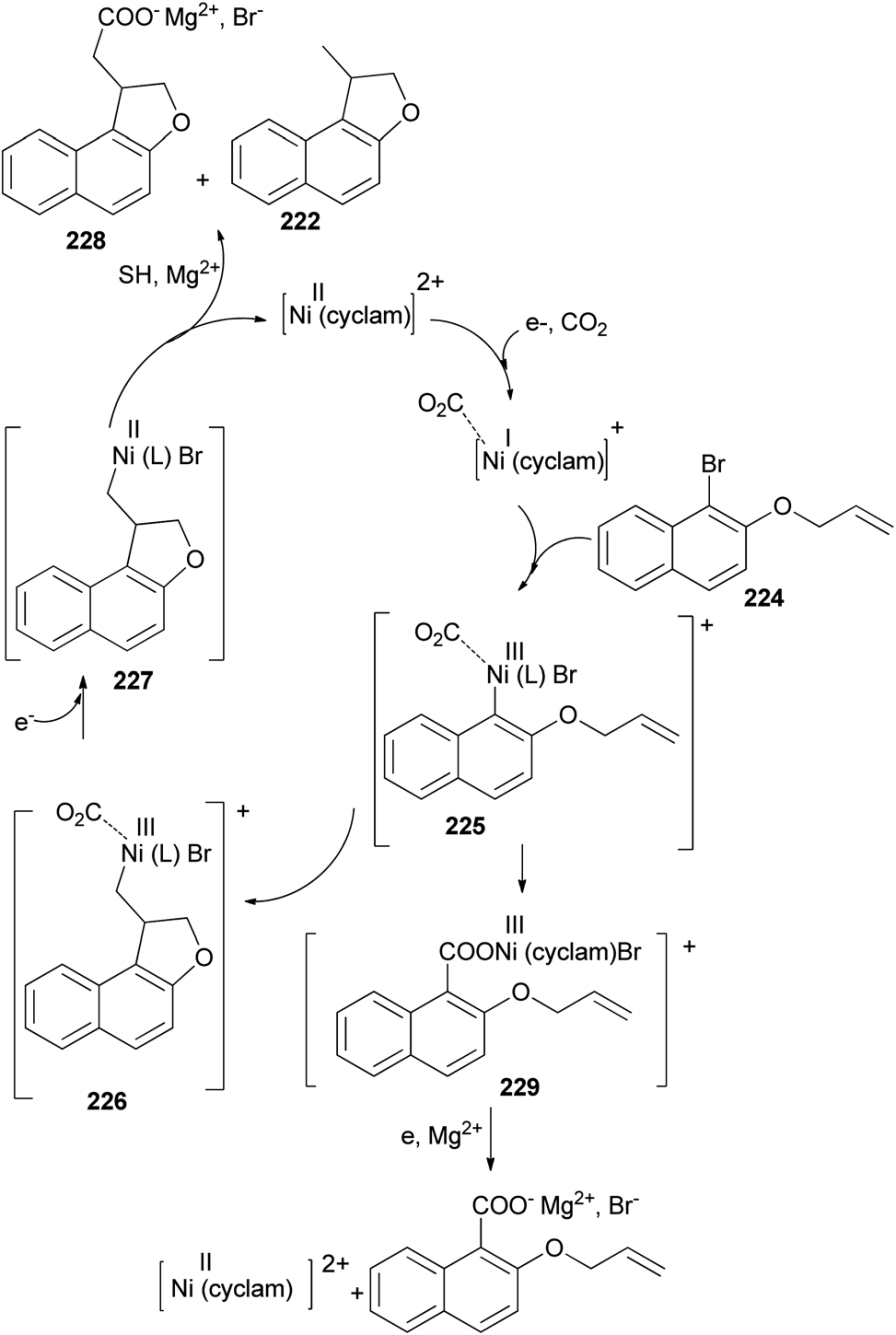
Proposed mechanism for the synthesis of 221–223.

The electrochemical reduction of 2-(allyloxy)-1-bromonaphthalene under CO_2_ pressure (1 atm) allows the synthesis of dihydronaphthofurans 221 and 222 in 8 and 47% yields, respectively. This novel intramolecular cyclization–carboxylation reaction is carried out in single-compartment cells (Mg/C as the electrodes) at 20 °C and is catalyzed by[Ni(cyclam)Br_2_ 2BF_4_^−^]. In this method, three types of products 221–223 were obtained as indicated in Scheme 103.^81^ A catalytic cycle is proposed in Scheme 104. The electrochemical reduction of Ni(cyclam)^2+^ affords Ni^I^ species. Oxidative addition of the Ni^I^ species (coordinated to CO_2_) to the C-(aryl)-halogen bond of 224. After oxidative addition, the formation of Ni^III^ intermediate species 225 is proposed. This Ni^III^ intermediate induces a radical character to the aryl moiety, resulting in a rapid intramolecular cyclization on the double bond in the side chain, leading to 226. Further one-electron reduction of Ni^III^ intermediate species 226 should form an alkylnickel(ii) species 227, able to undergo CO_2_ uptake, forming a nickel(ii) carboxylate. Competitive protonation of 227 by the electrolytic medium (solvent) affords cyclized, noncarboxylated 222. In the one-compartment cell procedure with a magnesium anode, the presence of the magnesium ions issued from the anodic oxidation process, enables the formation of a magnesium carboxylate 228 liberating the nickel species for further recycling. Magnesium carboxylate 228 is stable under the reaction conditions, it accumulates during electrolysis and it is simply hydrolyzed to 221 at the end of the reaction. In a competitive reaction, intermediate 225 can also be directly reduced and carboxylated to afford the corresponding carboxylic acid derivatives 223*via* intermediate 229.^81^

### 2.10. Synthesis by radical methods

**Scheme 105:**
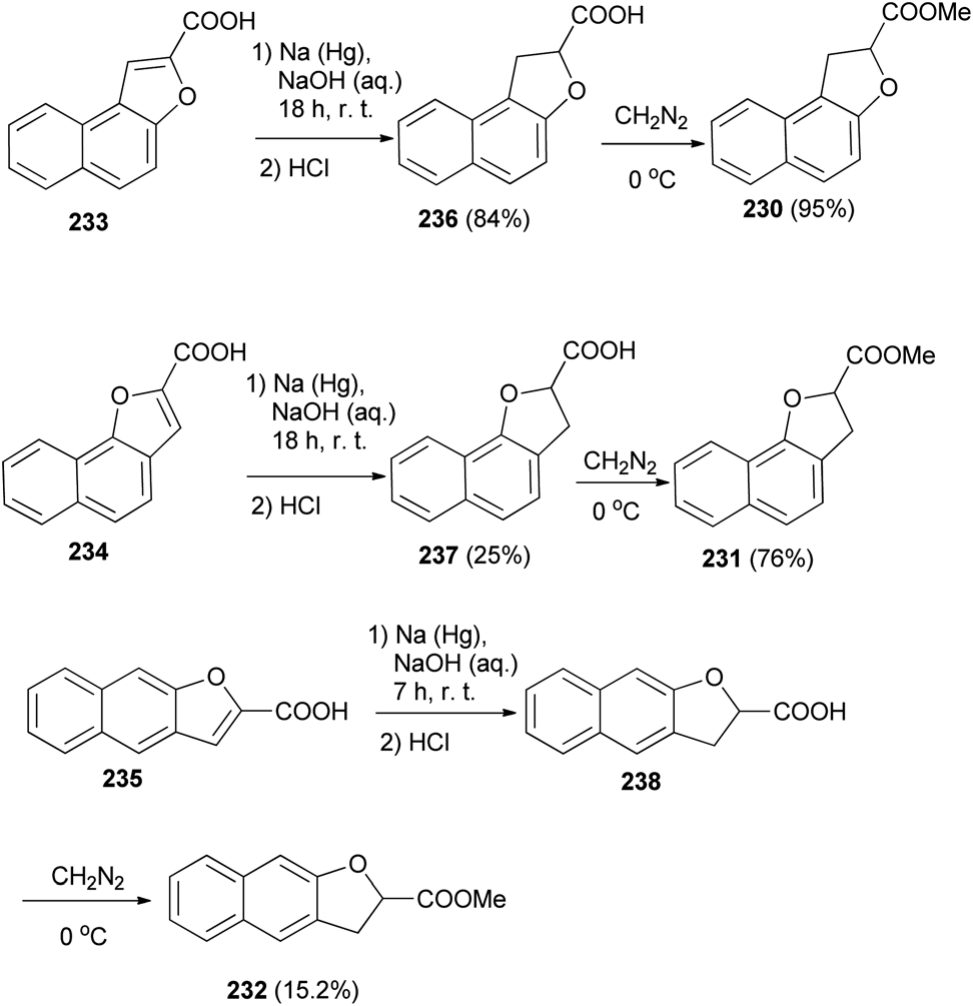
Synthesis of dihydronaphthofuran 230–232 and 236–238.

The work of Hayashi and Lawson^82^ demonstrated that various dihydronaphthofurans 230–232 were prepared by reaction of naphthofuran carboxylic acids 233–235 with sodium amalgam in alkali solution of NaOH at room temperature for 7 and 18 h led to the formation of dihydronaphthofuran carboxylic acids 236–238. Then, dihydronaphthofuran carboxylic acids were treated with an excess of ethereal diazomethane at 0 °C to give 230–232 in 95, 76 and 15.2% yields, respectively (Scheme 105).

**Scheme 106:**
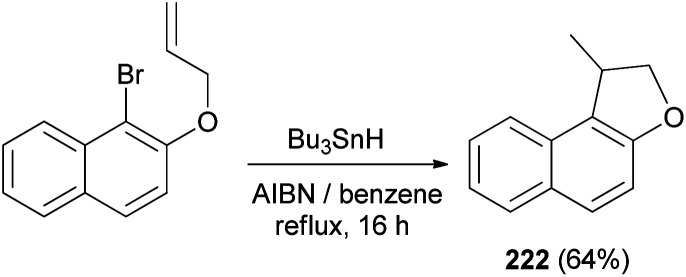
Synthesis of dihydronaphthofuran 222.

**Scheme 107:**
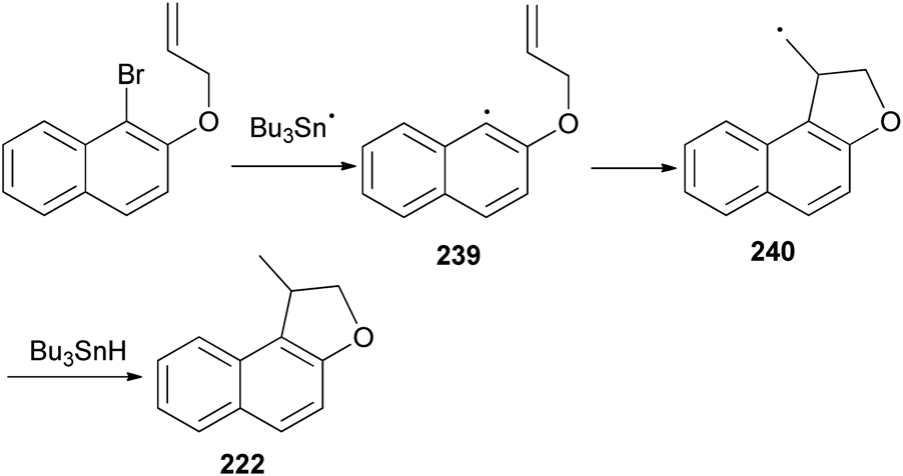
Plausible mechanism for the formation of 222.

Cyclization of 1-bromo-2-(prop-2-enyloxy)naphthalene was employed by Beckwith *et al.* to the synthesis of 1-methyl-1,2-dihydronaphtho[2,1-*b*]furan (222) in 64% yield. The reaction was carried out in the presence of Bu_3_SnH and AIBN as initiator in dry benzene under reflux condition for 16 h (Scheme 106). Presumably the mechanism involves generation of the radical 239 from 1-bromo-2-(prop-2-enyloxy)naphthalene followed by cyclization of radical 239 to 240 and transfer of a hydrogen atom from Bu_3_SnH to the primary radical 240 afforded the final product 222 according to the pathway given in Scheme 107.^83^

**Scheme 108:**
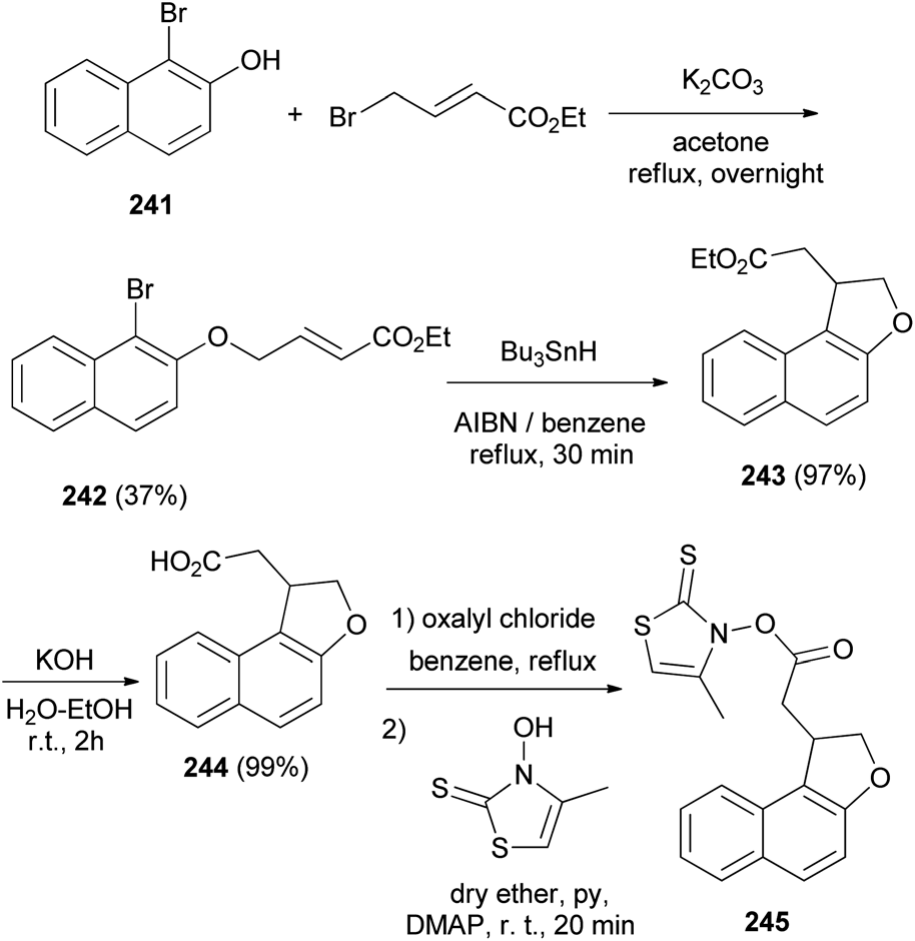
Preparation of dihydronaphthofurans 243–245.

Similarly, it was noted that reaction of ethyl 1-bromo-2-hydroxynaphthalene (241) with ethyl-4-bromocrotonate and K_2_CO_3_ in dry acetone under reflux for overnight to afford ethyl 4-(l-bromo-2-naphthyl)but-2-enoate (242) in 37% yield. The reaction of compound 242 with Bu_3_SnH and AIBN in refluxing dry benzene for 30 min gave ethyl 1,2-dihydronaphtho[2,l-*b*]furan-*l*-acetate (243) in 97% yield. Treatment of 243 with KOH in water–ethanol (1 : 1 v/v) at room temperature for 2 h resulted l,2-dihydronaphtho[2,1-*b*]furan-*l*-acetic acid (244) in 99% yield. The conversion of 244 into thiohydroxamate 245 can be efficiently performed in refluxing benzene using oxalyl chloride and then in dry ether using 3-hydroxy-4-methylthiazole-2(3*H*)-thione, pyridine and DMAP at room temperature for 20 min (Scheme 108).^83^

**Scheme 109:**
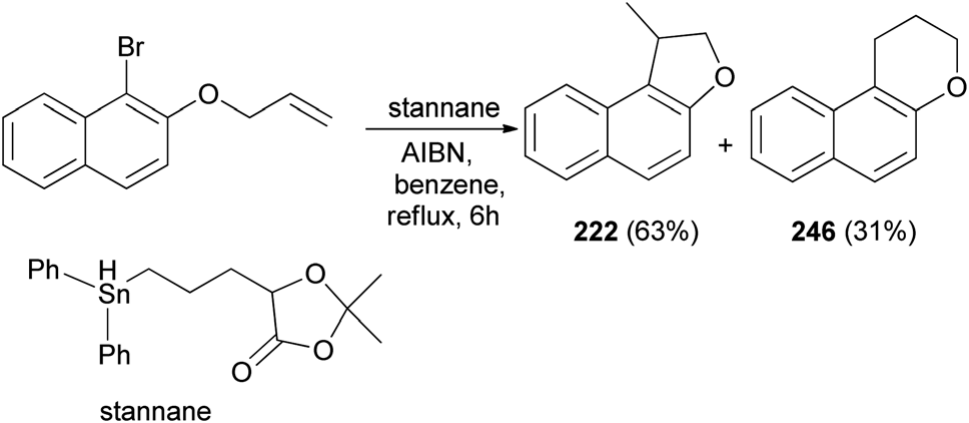
Synthesis of dihydronaphthofuran 222.

Clive and Wang^84^ demonstrated that 1-methyl-1,2-dihydronaphtho[2,1-*b*]furan (222) in 63% yield and 2,3-dihydro-1*H*-naphtho[2,1-*b*]pyran (246) in 31% yield were synthesized by radical cyclization of 2-(allyloxy)-1-bromonaphthalene in the presence of stannane and AIBN in refluxing benzene for 6 h (Scheme 109).

**Scheme 110:**
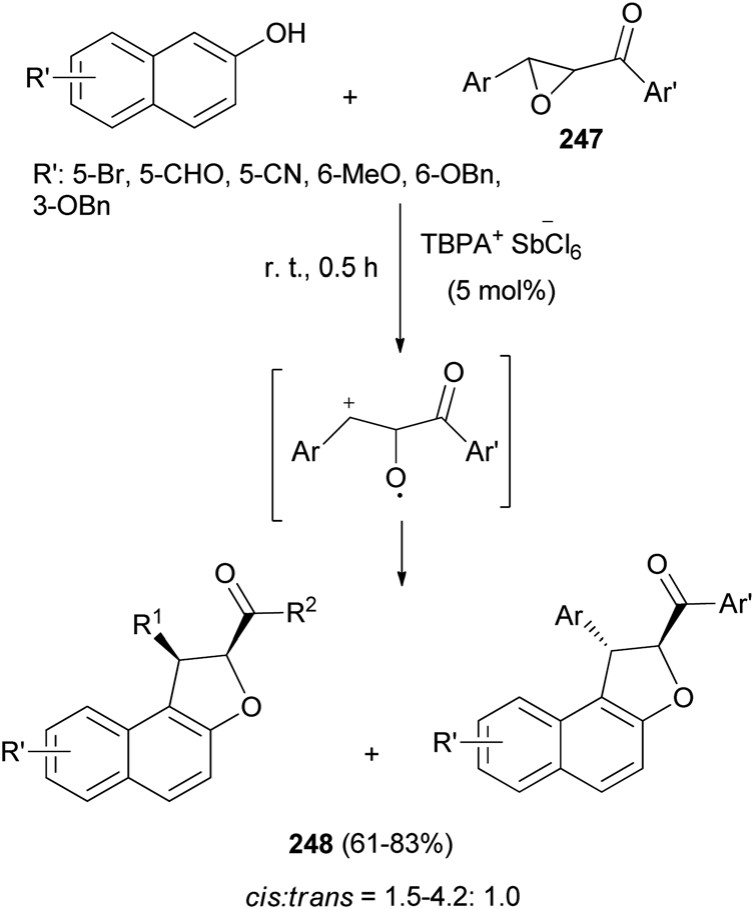
TBPA^+^·SbCl_6_^−^-catalyzed synthesis of 1,2-dihydronaphtho[2,1-*b*]furans 248.

Triarylaminium salt was disclosed as an efficient initiator for the novel Friedel–Crafts alkylation/annulation cascade reaction between chalcone epoxides 247 and 2-naphthols in CHCl_3_ at room temperature for 0.5 h to construct diastereomer polysubstituted 1,2-dihydronaphtho[2,1-*b*]furans 248 in 61–83% yields (Scheme 110). In this process, chalcone epoxides 247 acted as valuable halogen-free pre-electrophiles.^85^

**Scheme 111:**
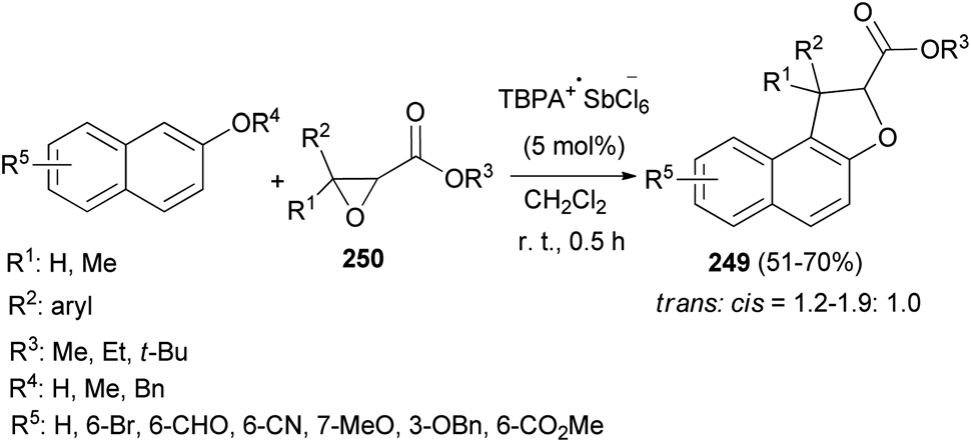
TBPA^+^·SbCl_6_^−^-catalyzed synthesis of *trans*/*cis*-dihydronaphthofurans 249.

**Scheme 112:**
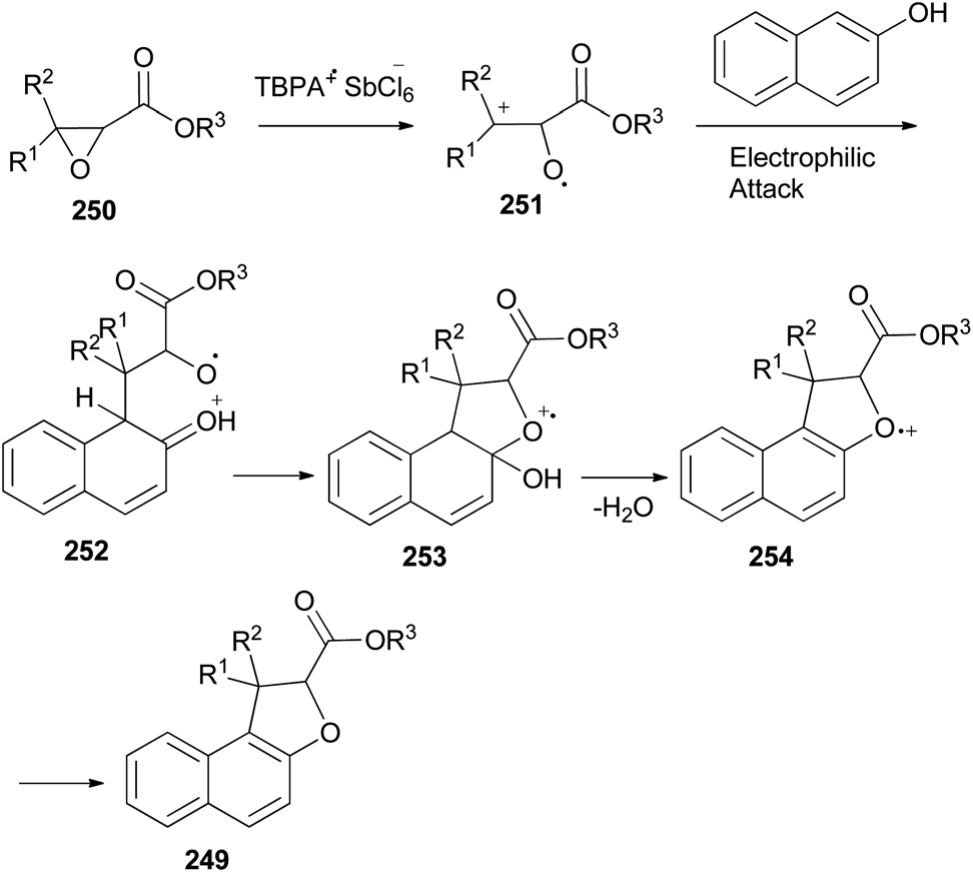
Proposed mechanism for the synthesis of 249.

An efficient chemoselective tandem Friedel–Crafts alkylation/cyclization reaction of glycidic esters with 2-naphthol derivatives in CH_2_Cl_2_ at ambient temperature for 0.5 h initiated by stable radical cation triarylaminium salt [tris(4-bromophenyl)aminium hexachloroantimonate, TBPA^+^·SbCl_6_^−^] (0.5 mol%) led to the formation of the mixture of *trans*/*cis*-dihydronaphthofurans 249 in 51–70% yields (Scheme 111). A plausible mechanism for formation of 249 is depicted in Scheme 112. The glycidic ester (250) was first activated by single electron oxidation to give distonic radical cation intermediate 251. 2-Naphthol (as an example) was then attacked by the benzylic cation of intermediate 251 to form the dihydrofurylium distonic radical cation intermediate 252. Then oxygen radical combined with the carbocation to construct radical cation intermediate 253. And then the radical cation intermediate 254 was formed by eliminating a molecule of water from intermediate 253. Intermediate 254 would then undergo single electron transfer from substrate 250 to produce the major product 249 and regenerate intermediate 251 at the same time. Chain propagation continued until all substrates were converted into the products.^86^

**Scheme 113:**
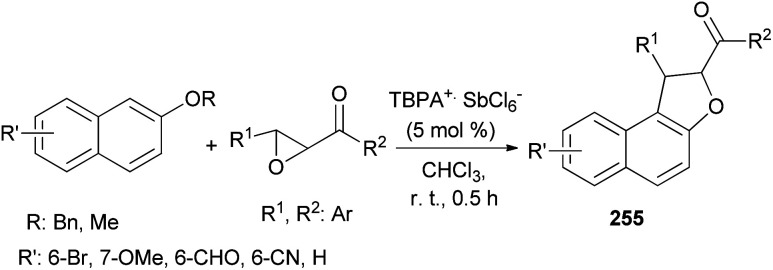
TBPA^+^·SbCl_6_^−^-catalyzed synthesis of dihydronaphthofurans 255.

Wang *et al.*^87^ concluded that tandem reaction between chalcone epoxides and 2-naphthyl ethers in the presence of stable triarylaminium salt (5 mol%) in CHCl_3_ at room temperature for 0.5 h afforded dihydronaphthofurans 255 (Scheme 113). And after subsequent aerobic oxidative aromatization in one pot, a series of polysubstituted naphtho[2,1-*b*]furans were delivered. It should be noted that compounds 255 have not isolated. The reaction mechanism similar to the proposed mechanism for the synthesis of 249.

### 2.11. Other synthetic methods

**Scheme 114:**
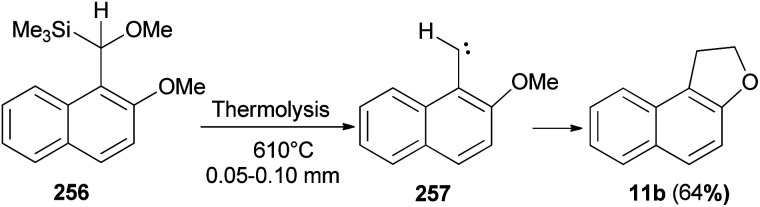
Thermolysis of compound 256 to 1,2-dihydro-naphtho[2,l-*b*]furan (11b).

Thermolysis of [methoxy(2-methoxy-*l*-naphthyl)methyl]trimethylsilane (256) at 610 °C/0.05–0.10 mm yields 1,2-dihydronaphtho[2,l-*b*]furan (11b) in 64% yield presumably by insertion of 2-methoxy-1-naphthylmethylene (257) into a C–H bond of its *o*-methoxy group (Scheme 114).^88^

**Scheme 115:**
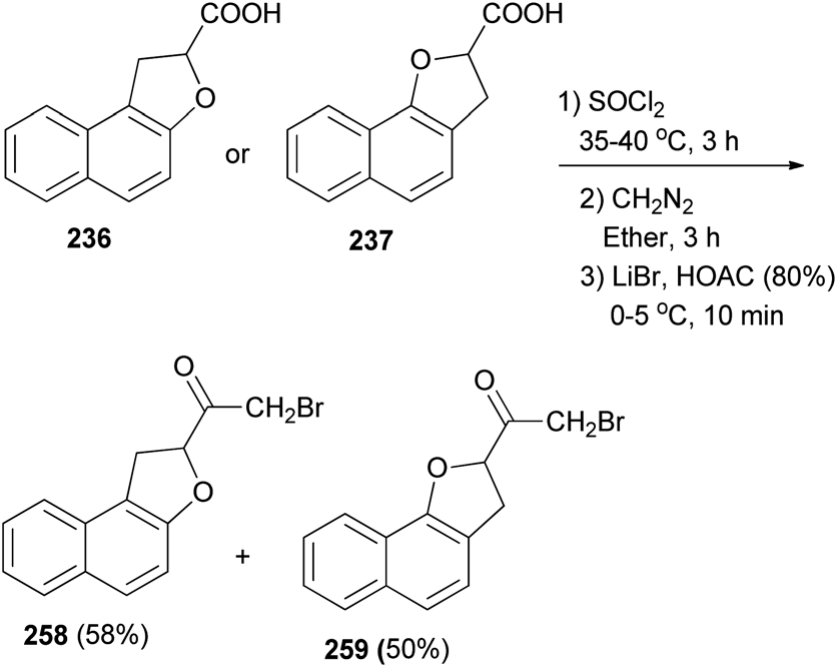
Synthesis of dihydronaphthofurans 258 and 259.

2-Bromo-1-(1,2-dihydronaphtho[2,1-*b*]furan-2-yl)ethanone (258) in 58% yield and 2-bromo-1-(2,3-dihydronaphtho[1,2-*b*]furan-2-yl)ethanone (259) in 50% yield have been synthesized from compounds 236 and 237 with SOCl_2_ at 35–40 °C for 3 h. Subsequently, the crude acid chlorides in diethyl ether were treated with diazomethane for 3 h and after that with LiBr in HOAC (80%) at 0–5 °C for 10 min (Scheme 115).^13^

**Scheme 116:**
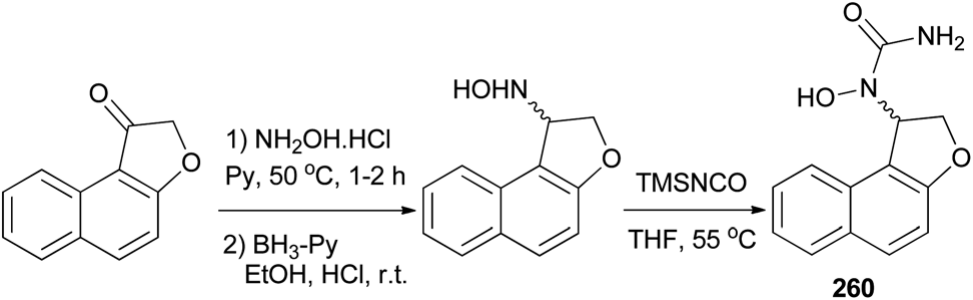
Synthesis of *N*-hydroxyurea of dihydronaphthofuran 260.

Admas *et al.*^7^ demonstrated that *N*-hydroxyurea derivative of dihydronaphthofuran 260 has been prepared in 12% yield from the corresponding ketone in three steps, oximation, reduction, and hydroxyurea formation (Scheme 116).

**Scheme 117:**
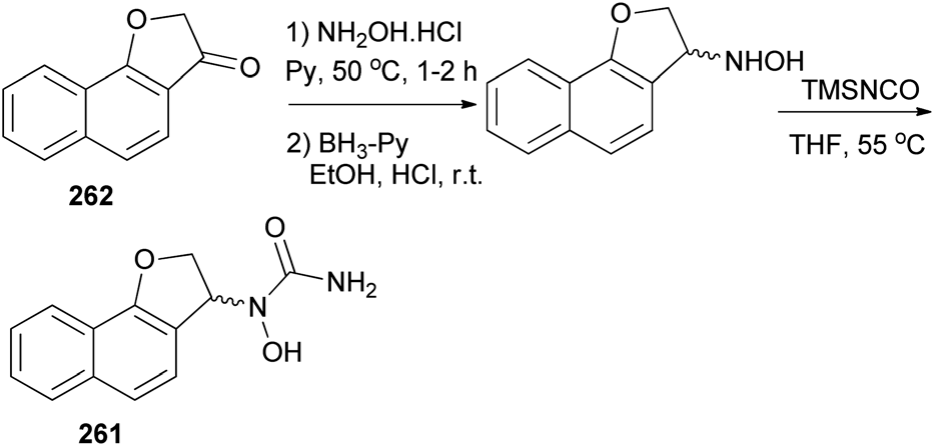
Synthesis of *N*-hydroxyurea derivative of dihydronaphthofuran 261.

In a similar fashion, *N*-hydroxyurea derivative of dihydronaphthofuran 261 could be prepared in 18% yield from the corresponding ketone 262 in three steps, oximation, reduction, and hydroxyurea formation (Scheme 117).

**Scheme 118:**
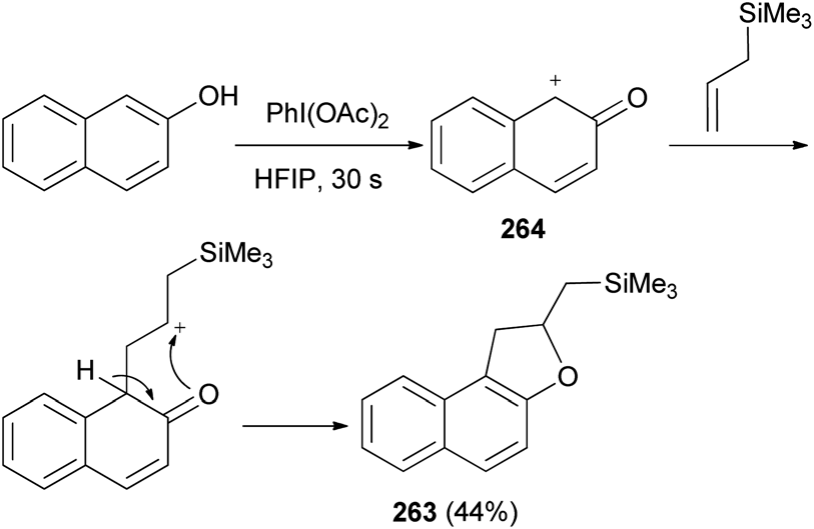
Iodobenzene diacetate catalyzed synthesis of 1,2-dihydronaphtho [2,1-*b*]furan 263.

Canesi *et al.*^89^ demonstrated that treatment of 2-naphthol with allylsilane in hexafluoroisopropanol (HFIP) for 30 s in the presence of iodobenzene diacetate gave ((1,2-dihydronaphtho[2,1-*b*]furan-2-yl)methyl)trimethylsilane (263) in 44% yield. This reaction proceed *via* formal [2 + 3] cycloaddition is based on a common electrophilic intermediate 264 generated by an environmentally benign hypervalent iodine reagent in perfluorinated protic solvents. This intermediate can react with different sufficiently reactive allylsilane to lead in one step to different heterocyclic rings such as dihydronaphthofuran 263 (Scheme 118).

**Scheme 119:**
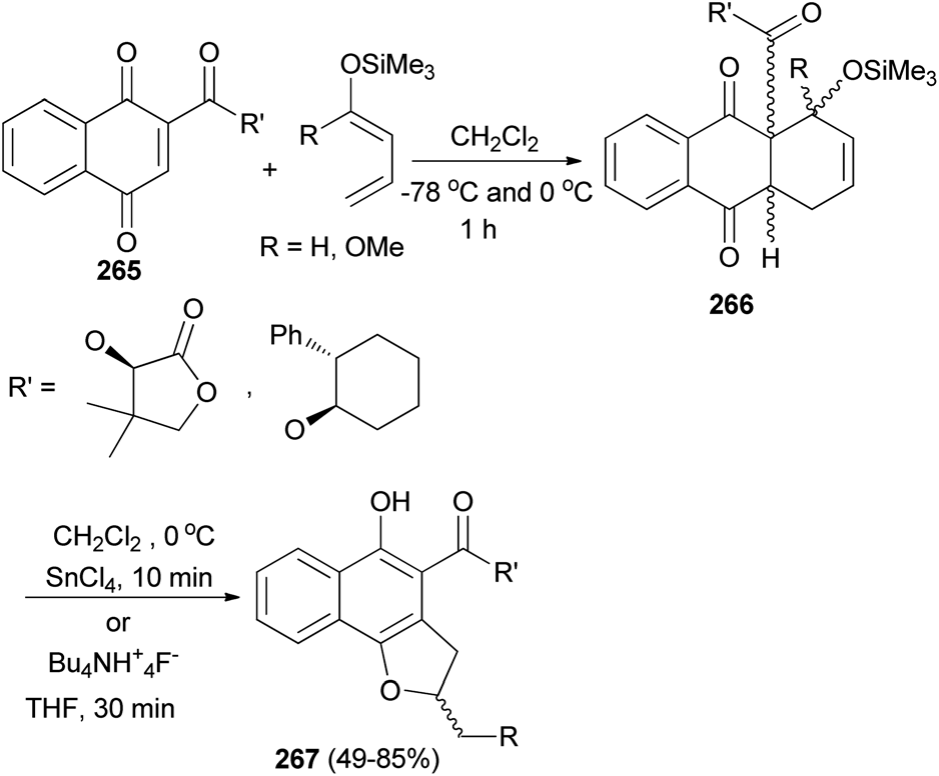
Preparation of 2,3-dihydronaphtho[1,2-*b*]furans 267.

The Diels–Alder addition of naphthoquinone 265 to 1-trimethylsilyloxybuta-1,3-diene in CH_2_Cl_2_ at −78 °C and 0 °C for 1 h afforded Diels–Alder adducts 266. Individual treatment of adducts 266 with tin(iv) chloride in CH_2_Cl_2_ at 0 °C for 10 min or with tetrabutyl ammonium fluoride in THF for 30 min afforded 2,3-dihydronaphtho[1,2-*b*]furan 267 in 49–85% yields (Scheme 119).^90^

**Scheme 120:**
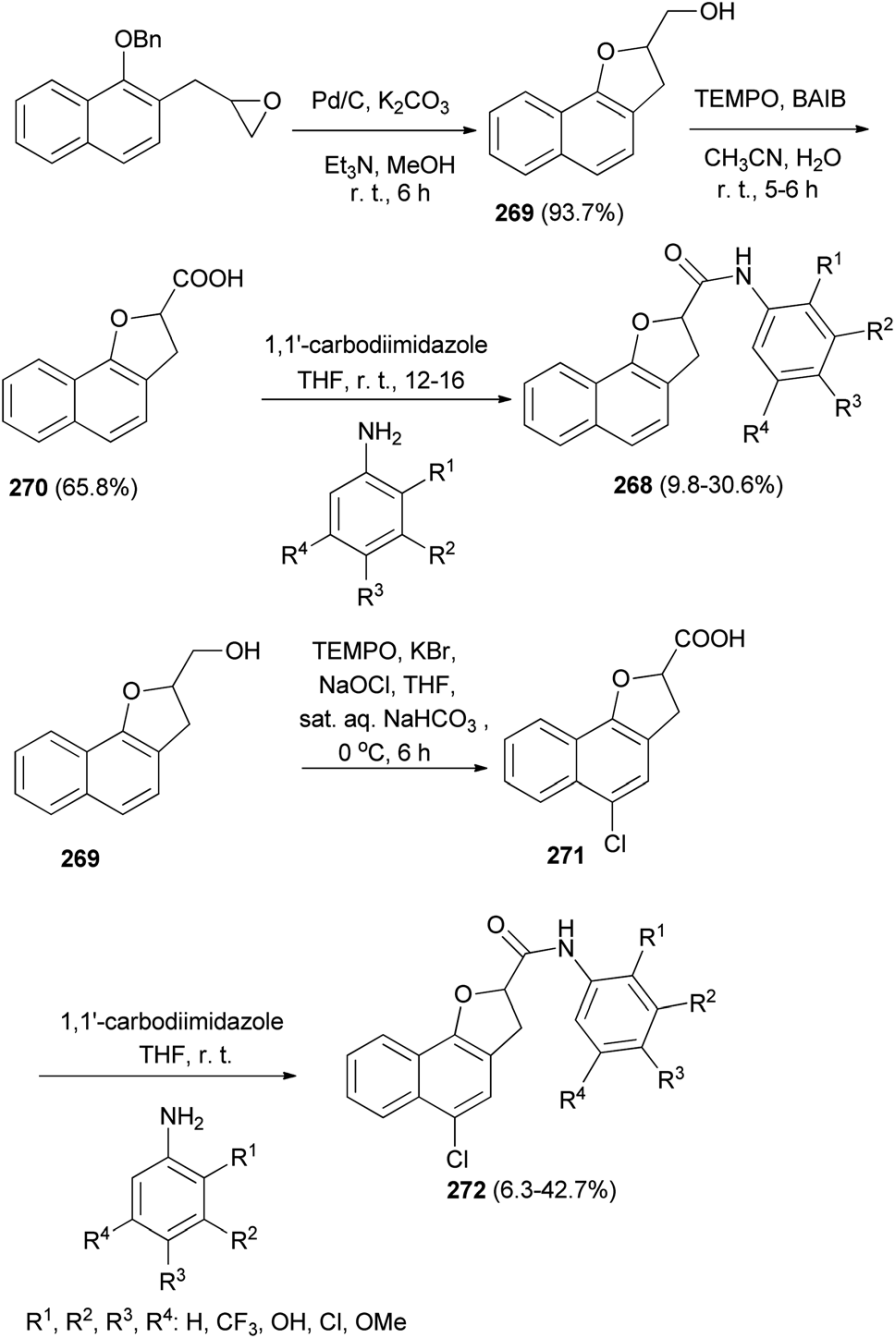
Synthesis of 2,3-dihydro- and 5-chloro-2,3-dihydro-naphtho-[1,2-*b*]furan-2-carboxylic acid *N*-(substituted phenyl)amide analogs 268 and 272.

A series of 2,3-dihydronaphtho[1,2-*b*]furan-2-carboxylic acid *N*-substituted phenyl amide analogs 268 were synthesized by Kwak *et al.*^11^ A mixture of 2-((1-(benzyloxy)naphthalen-2-yl)methyl)oxirane, 10% Pd/C, K_2_CO_3_, Et_3_N (catalytic amount) and MeOH was stirred for 8 h at room temperature afforded (2,3-dihydronaphtho[1,2-*b*]furan-2-yl)methanol (269) in 93.7% yield. Treatment of 269 with TEMPO and BAIB in CH_3_CN/H_2_O at room temperature for 5–6 h gave 2,3-dihydronaphtho[1,2-*b*]furan-2-carboxylic acid (270) in 65.8% yield. Stirring a mixture of 270 and 1,1′-carbodiimidazole in anhydrous THF for 1 h followed by addition of substituted aniline to the reaction mixture at room temperature and stirring for 12–16 h afforded 2,3-dihydronaphtho[1,2-*b*]furan-2-carboxylic acid *N*-(substituted phenyl)amide analogs 268 in 9.8–30.6% yields. Also, treatment of 269 with TEMPO, KBr, NaOCl, THF and sat. aq. NaHCO_3_ at 0 °C for 6 h gave 5-chloro-2,3-dihydronaphtho-[1,2-*b*]furan-2-carboxylic acid (271); then addition of 1,1′-carbodiimidazole in anhydrous THF and substituted aniline at room temperature to the reaction mixture led to the formation of 5-chloro-2,3-dihydronaphtho-[1,2-*b*]furan-2-carboxylic acid *N*-(substituted phenyl)amide derivatives 272 in 6.3–42.7% yields (Scheme 120).

**Scheme 121:**
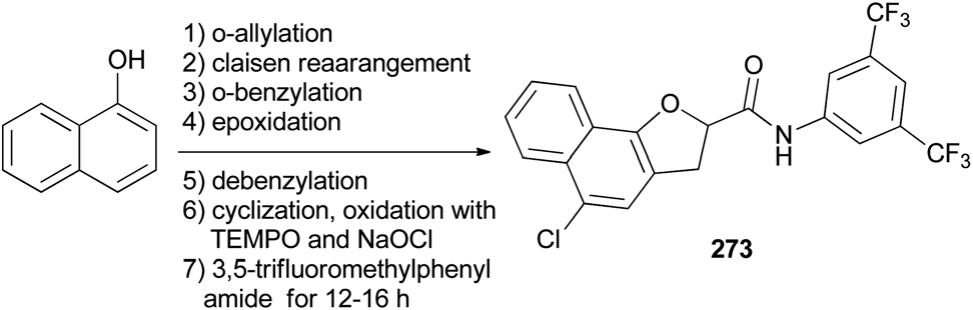
Synthesis of *N*-(3,5-bis(trifluoromethyl)phenyl)-5-chloro-2,3-dihydronaphtho[1,2-*b*]furan-2-carboxamide (NHDC) (273).

Hong and coworkers reported that synthesis of *N*-(3,5-bis(trifluoromethyl)phenyl)-5-chloro-2,3-dihydronaphtho[1,2-*b*]furan-2-carboxamide (NHDC) (273) from 1-naphthol through a series of reactions including *o*-allylation, Claisen rearrangement, *o*-benzylation, epoxidation of allyl compound, debenzylation followed by cyclization, oxidation with TEMPO and NaOCl, and amide coupling with 3,5-trifluoromethylphenylamide for 12–16 h (Scheme 121).^14^

**Scheme 122:**
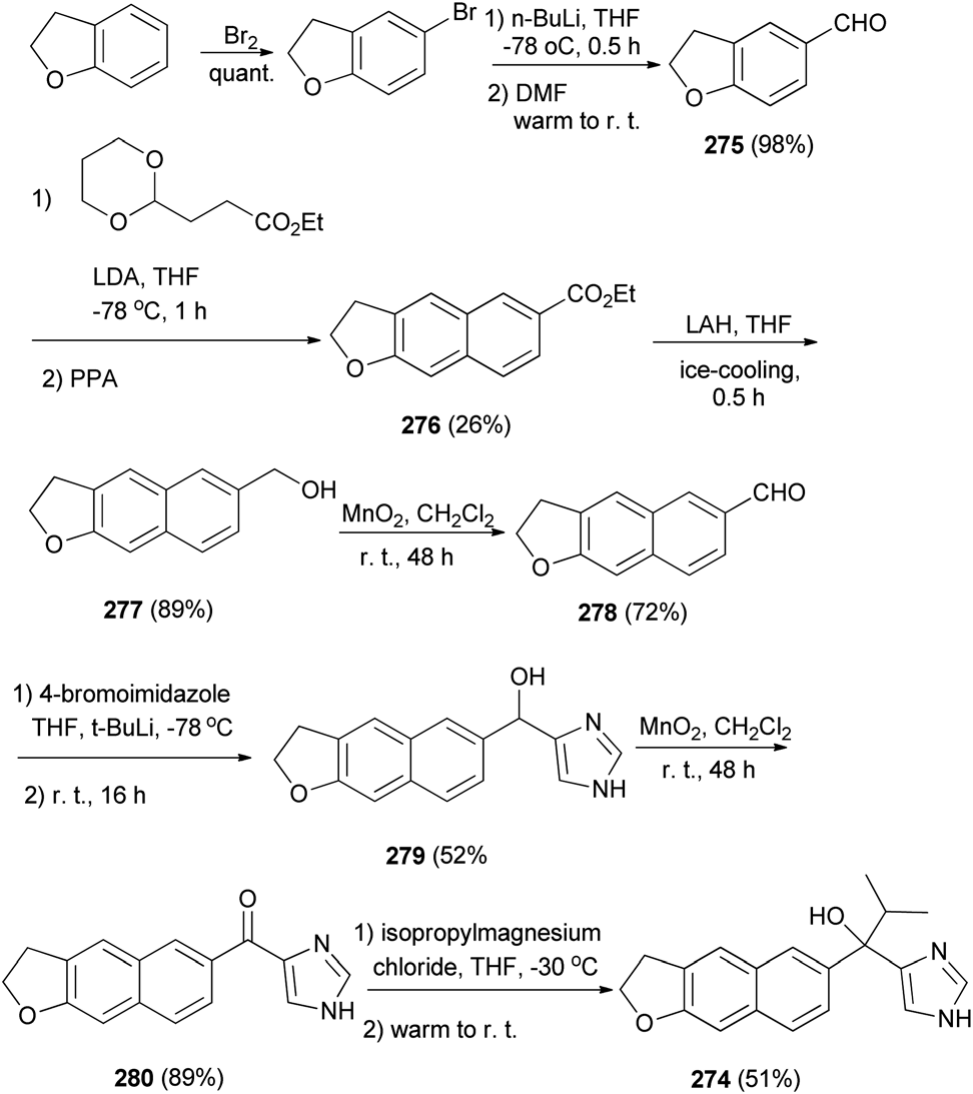
Synthesis of 2,3-dihydronaphtho[2,3-*b*]furans 274 and 276–280.

2,3-Dihydronaphtho[2,3-*b*]furan derivative 274 was synthesized as shown in Scheme 122. Bromination of 2,3-dihydro-1-benzofuran followed by formylation gave aldehyde 275, which was converted to 276 by treatment with acetal ester. Reduction of 276 by LAH afforded 2,3-dihydronaphtho[2,3-*b*]furan derivative 277 and then oxidation with MnO_2_ led to the formation of aldehyde 278. Since Katritzky's imidazole introduction into 278 afforded alcohol 279, followed by oxidation of alcohol to the ketone group with MnO_2_ leading to the formation of product 280. A Grignard reaction of 280 with i-PrMgCl provided 274 in 51% yield.^8^

**Scheme 123:**
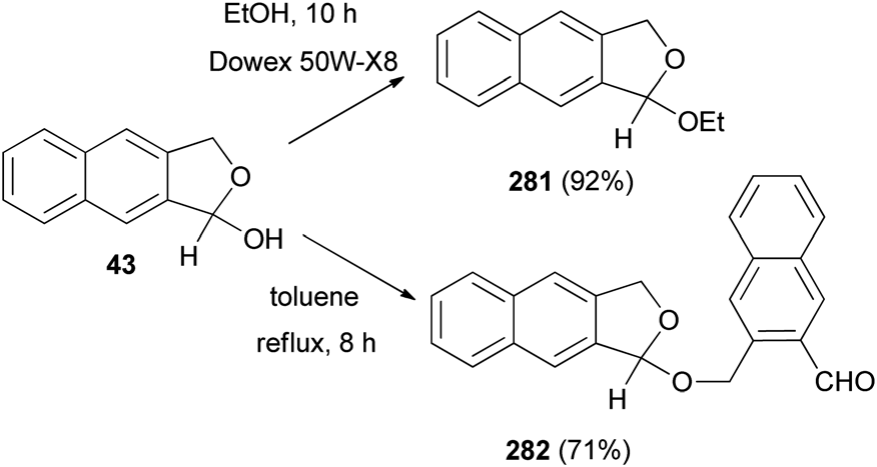
Preparation of dihydronaphthofurans 281 and 282.

Interaction of 43 with absolute ethanol in the presence of Dowex 50W-X8 resin under reflux condition for 10 h gave 1-ethoxy-1,3-dihydronaphtho[2,3-*c*]furan (281) in 92% yield. Also, treatment of 43 in a flask equipped with a Dean–Stark trap in refluxing toluene for 8 h afforded 1-[(3-formyl-2-naphthalenyl)methoxy]-1,3-dihydronaphtho[2,3-*c*]furan (282) in 71% yield (Scheme 123).^32^

## 3. Dihydronaphthofuran applications

### 3.1. Biological activities

Dihydronaphthofuran derivatives have received considerable attention in view of the interesting biological and pharmacological properties associated with this heterocyclic scaffold. For example, *N*-hydroxyurea derivatives of dihydronaphthofurans 260 and 261, which were weaker inhibitors of 5-lipoxygenase, had IC_50_ values in excess of 10 μM in human whole blood.^7^ A series of dihydronaphthofuran derivatives such as 53 and 274 evaluated as C_17,20_-lyase inhibitors. 1,2-Dihydronaphtho[2,1-*b*]furan 53 showed equipotent C_17,20_-lyase inhibition activities on rate and human 26 and 25, respectively, and 11β-hydroxylase activity on rate 77. Also, 2,3-dihydronaphtho[2,3-*b*]furan 261 showed equipotent C_17,20_-lyase inhibition activities on rate and human 21 and 23, respectively, and 11β-hydroxylase activity on rate 350.^8^ Many functionalized dihydronaphthofurans 81 and 82 are widely employed as antitubercular activity against *Mycobacterium tuberculosis* H_37_Rv. 2-Azidomethyldihydronaphthofurans 81 proved to be potent antitubercular with MIC 3.12 mg ml^−1^ comparable to the standard drug ethambutol. Among 1-(2,3-dihydronaphthofuran-2-yl-methyl)[1,2,3]triazoles only *n*-pentyl substituent on compound 82 exhibited mild antitubercular activity with MIC 12.5 mg ml^−1^.^9^ Furthermore, dihydronaphthofuran 258 exhibit a powerful inhibitor for α-chimotrypsine. This compound inactivate the enzyme rapidly and its action was stereo specific, confirming that the binding site of α-chimotrypsine is planar, elongated and curve.^13^ Evaluating of biological properties for the synthesized 5-hydroxy-4-acetyl-2,3-dihydronaphtho[1,2-*b*]furans 99 indicated that they have anti-tyrosinase, antioxidant, and antibacterial activities.^10^ The synthesized furomollugin analogs 100 were evaluated for direct scavenging activity against a variety of reactive oxygen and nitrogen species, such as 1,1-diphenyl-2-picrylhyrazyl (DPPH), nitric oxide (NO), and hydrogen peroxide (H_2_O_2_) assays. The results showed that they had effective antioxidant power. Moreover, the antibacterial activities of those compounds were also evaluated and the highly active compounds were selected for further determination of minimal inhibitory concentrations (MICs). It was found that they have highly active against the Gram negative bacteria *Escherichia coli* (KCTC-1924) and inhibited Gram-positive bacteria *Staphylococcus aureus* (KCTC-1916) growth.^50^ A series of 2,3-dihydronaphtho [1,2-*b*]furan-2-carboxylic acid *N*-substituted phenyl amide analogs such as 2,3-dihydro-and 5-chloro-2,3-dihydro-naphtho-[1,2-*b*]furan-2-carboxylic acid *N*-(substituted phenyl)amide 268 and 272 showed biological activities such as inhibitors of NF-_k_B activity and anticancer agents. In addition, these compounds exhibited potent cytotoxicity at low concentrations against HCT-116, NCI-H23, and PC-3 cell lines.^11^*N*-(3,5-bis(trifluoromethyl)phenyl)-5-chloro-2,3-dihydronaphtho[1,2-*b*]furan-2-carboxamide (NHDC) (273) inhibits liver tumor growth through activation of HNF 4α.^14^ Treatment with different concentrations (1–10.8 μM) of NHDC for various periods (0–72 h) inhibited liver cancer cells (HepG2, Hep3B) growth as well as colony formation followed by induction of apoptosis in a concentration dependent manner. NHDC also induced expression of the apoptosis regulating genes as well as inhibiting the action of STAT3. These inhibitory effects were associated with enhancement of expression and DNA binding activity of HNF 4α.^14^ A series of 1,2-dihydronaphtho[2,1-*b*]furan derivatives such as 154 and 155 was synthesized as analogues of known natural α-glucosidase inhibitors. They have demonstrated significant potency with IC_50_ values ranging from 6.50 to 722.2 μM, as well as hypoglycemic activity exceeding the reference drug acarbose.^12^

### 3.2. Photochromic properties

**Scheme 124:**
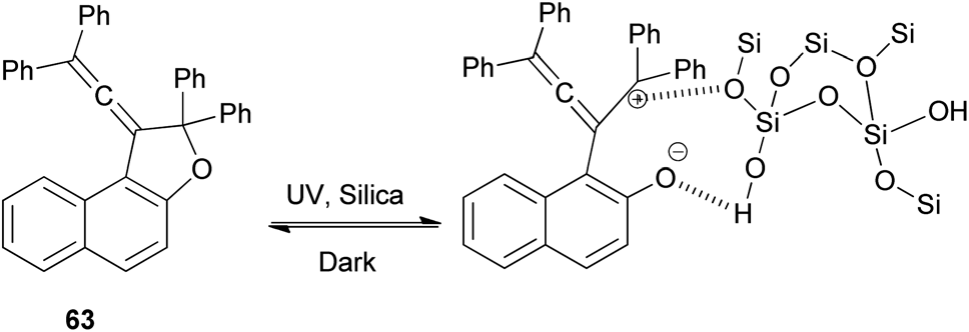
Photochromic equilibrium for 1-vinylidenenaphtho[2,1-*b*]furan 63.

**Scheme 125:**
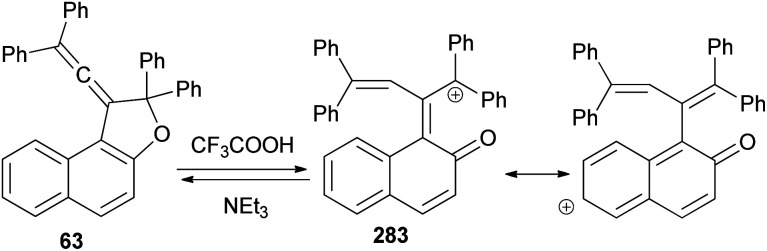
Acidochromism of 1-vinylidenenaphtho[2,1-*b*]furan 63.

Vinylidene-naphthofurans are a new class of photochromic molecules, with a unique structure combining an allene group linked to a dihydrofuran ring. These uncoloured molecules show acidochromism in solution and photochromic properties when adsorbed in silica gel or dissolved in acidified alcoholic solutions but not in common solvents or in the solid state. Mechanism of the photochromic behaviour for those compounds is thermally reversible. For example, vinylidene-naphthofuran 63 exhibit photochromism at room temperature when adsorbed in silica gel. UV or sunlight irradiation leads, in a few seconds, to the formation of intense pink/violet to green colors that bleach completely in a few minutes in the dark (Scheme 124). Also, compound 63 show acidochromic properties: addition of TFA to an uncolored solution of compound 63 leads to the immediate development of an intense violet coloration 283 that bleaches immediately when a weak base (NEt_3_) is added (Scheme 125).^15^

**Scheme 126:**
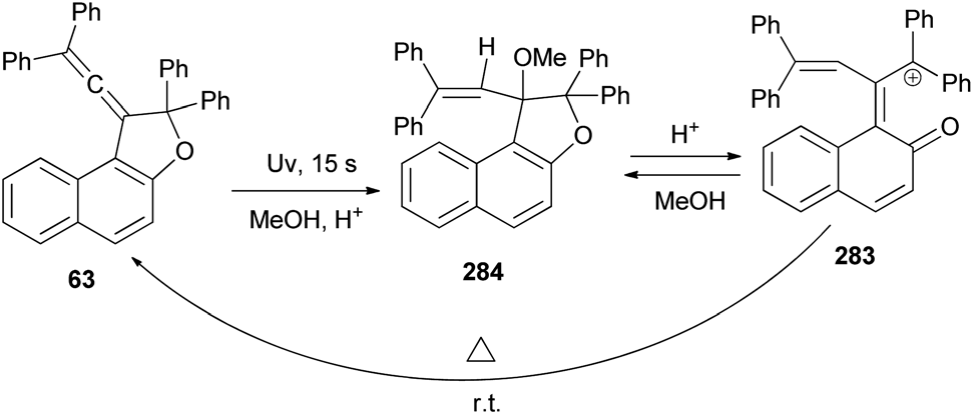
Mechanism of photochromism for 1-vinylidene-naphthofuran 63.

Vinylidene-naphthofurans 63 exhibit acidochromic properties in TFA solutions. They are converted into stable cationic species in strong acidic medium and bleach back to the uncoloured closed form upon neutralisation with Et_3_N. A mechanism for their thermally reversible photochromic behaviour is proposed based on NMR analysis of UV-irradiated CH_3_OD + THF-d_8_ acidified solutions: the UV light promotes the addition of methanol to the naphthofuran affording non-coloured photoproduct 284. In the presence of acid, the later is quickly converted into a cationic violet dye 283 that returns thermally to the initial closed naphthofuran in the dark. This photochromic system switches between the uncoloured and violet state after UV or sunlight exposure (15 s) and returns thermally to the initial uncoloured state in 2–8 min, in the dark, at room temperature (Scheme 126).^16^

**Scheme 127:**
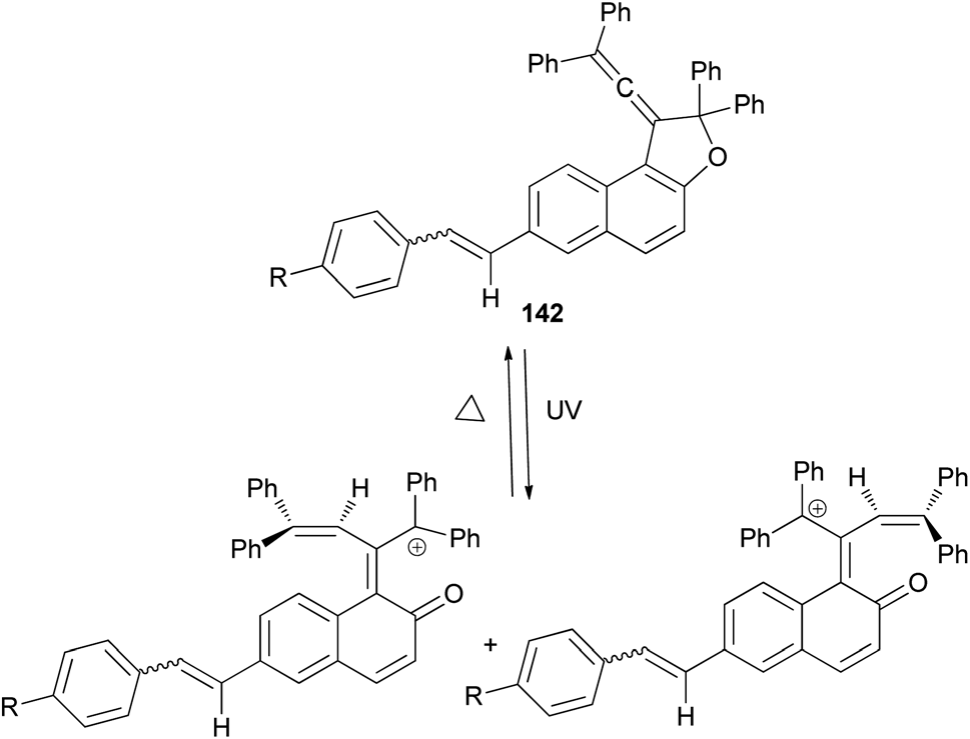
Photochromic equilibrium for 7-styryl-1-vinylidenenaphthofurans 142.

The introduction of a styryl chain in the structure of the vinylidene-naphthofurans such as 140–142 leads to a new set of uncoloured photochromic compounds that afford grey/brown colourations upon exposure to the UV or sunlight, at room temperature and returning to the uncoloured state, in the dark, in several minutes. The photochromic properties of these smart dyes are very sensitive to the chemical environment, specially their acidity being faster in THF/HCOOH solution than in silica gel. The amount of formic acid influences the kinetics of the fading reaction as a low concentration leads to a faster system but with a lower colourability. A good compromise was obtained in THF/HCOOH (2/0.5) solutions. The substituents in the styryl group influences the kinetic of the fading process: electron-withdrawing groups like Br or CF_3_ increases the fading rate leading to fast switching compounds but confers an initial yellowish colour to the solution before irradiation (Scheme 127).^17^

**Fig. 4:**
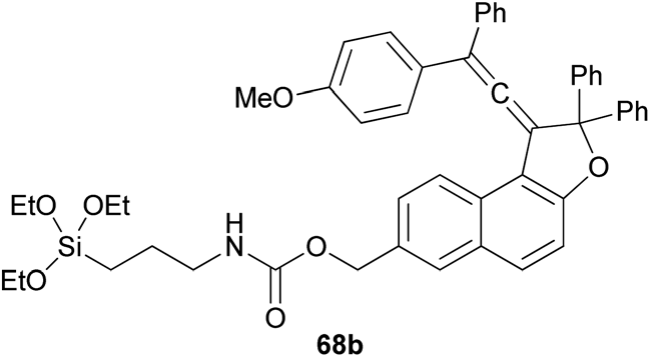
Structure of vinylidene-naphthofuran 68b.

The photochromic properties of the ormosil materials 68 showed a strong dependence on the nature of the silanes, spacer, acid and curing conditions. The functionalization of the vinylidene-naphthofurans with a reactive siloxane group was essential to avoid their precipitation after curing. The best results were obtain using a mixture of TEOS, MTES, PTES, 1,2-ethanodiol, water, chloroacetic acid and vinylidene-naphthofurans (68: R = OMe) cured at 50 °C for 7 days. This uncoloured material is transparent and develops an intense green colouration, characterized by two absorption bands at 460 and 640 nm, after 2 min under UV light. When the light source is removed a mono-exponential colour decay occurs due to the spontaneously ring closure reaction that afford the initial molecule. These hybrids showed a fast thermal bleaching kinetics, losing almost their total colouration in 20 min in the dark (Fig. 4).^19^

**Fig. 5:**
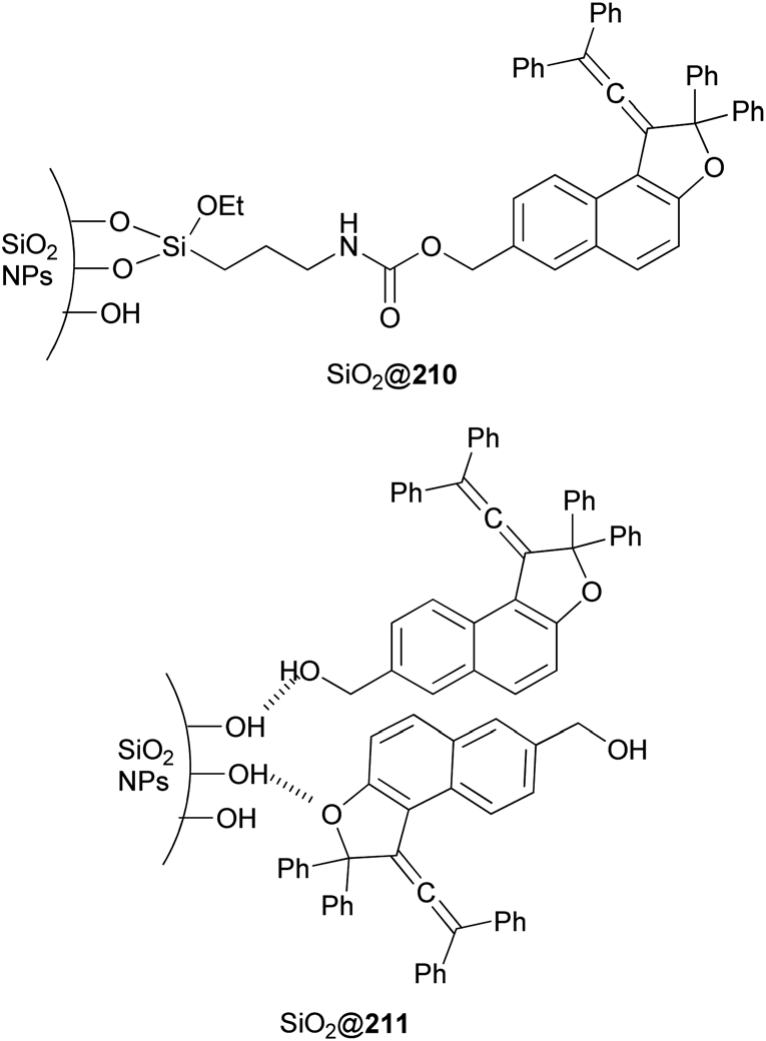
Schematic representation of the vinylidene-naphthofurans 210 and 211 immobilization onto SiO_2_ NPs.

A series of photochromic vinylidene-naphthofurans 88 and 89 with extended conjugation, embedded in ormosil matrices affording solid and transparent materials that acquire different colourations (violet, green, bluish), reversibly, when exposed to the UV (sun) light, for 2 min, at room temperature. The presence of an extra phenyl ring in some positions affects both the *λ*_max_ of absorption of the photochromic compounds in the uncoloured closed and open coloured form. After removal of the light source the materials lose progressively their colouration returning to the initial uncoloured state in less than 15 min at room temperature. Photochromic 1-vinylidene-naphtho[2,1-*b*]furan derivatives 210 and 211 were successfully anchored onto silica nanoparticles (SiO_2_ NPs) through direct adsorption (SiO_2_@211) and covalent post-grafting (SiO_2_@210) (Fig. 5).

**Scheme 128:**
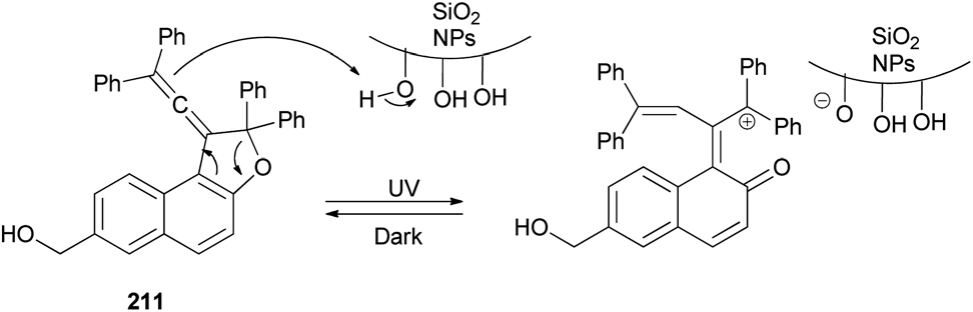
Proposal mechanism.

SiO_2_ NPs with different size and surface chemistry (pH_pzc_ in the range of 5–9) were used, offering a wide range of possibilities to fabricate tailor-made photochromic materials. The photochromic behavior of these new nanoparticles indicates that silica surface acidity and the type of vinylidene-naphthofuran immobilization strategy (adsorption *vs.* covalent grafting) were crucial factors for the occurrence of photochromism in the vinylidene-naphthofuran-based SiO_2_ NPs. Upon direct UV (*λ* = 365 nm) or sunlight exposure during 1 min, only the SiO_2_@211 nanomaterials prepared by direct vinylidene-naphthofuran adsorption onto SiO_2_ NPs with pH_pzc_ ≈ 6.0 showed direct and reversible photochromic properties, developing fast (in seconds) and intense salmon and violet coloration, with high values of total color difference and optical densities; in contrast, all nanomaterials prepared by covalent grafting of 210 onto SiO_2_ NPs (SiO_2_@210) did not exhibit photochromism. In the case of the photochromic SiO_2_@211 NPs, the decoloration process followed a bi-exponential decay with fast rate constants, which were responsible for the loss of coloration in less than 10 min. Furthermore, they presented very good resistance to fatigue, showing reversibility between the colored/uncolored states without significant loss of their performance for at least 8 successive UV/dark cycles. Scheme 128 has been represented of the proposed mechanism responsible for the photochromic behavior of 211 onto SiO_2_ NPs.^18^

## 4. Conclusions

In this review, wide range of synthetic strategies of dihydronaphthofurans (DHNs) as an important class of arene ring-fused furans has been discussed. We have started with chemical, photochemical and electrochemical methods for the synthesis of DHNs, followed by presenting of their diverse biological, pharmacological activities and photochromic properties. In general, naphthol derivatives play an important role and work well in construction of DHNs. Moreover, different types of reactions such as annulation of naphthols and naphthoquinones, [3 + 2] and [4 + 1] cycloaddition, Friedel–Crafts and Diels–Alder reactions, Claisen and neophyl rearrangement, cyclization of allyl naphthols and *etc.* were demonstrated for synthesis of DHNs. We believed that the reported methods could be of interest in material science, medicinal, photochromic compounds and natural products synthesis and their use has been growing rapidly.

## Conflicts of interest

There are no conflicts to declare.

## Supplementary Material
